# Systematic Profiling of mRNA Splicing Reveals the Prognostic Predictor and Potential Therapeutic Target for Glioblastoma Multiforme

**DOI:** 10.1155/2021/4664955

**Published:** 2021-07-05

**Authors:** Botao Zhang, Quanyou Wu, Shujun Cheng, Wenbin Li

**Affiliations:** ^1^Department of Neuro-oncology, Cancer Center, Beijing Tiantan Hospital, Capital Medical University, Beijing 100070, China; ^2^State Key Laboratory of Molecular Oncology, Department of Etiology and Carcinogenesis, National Cancer Center/National Clinical Research Center for Cancer/Cancer Hospital, Chinese Academy of Medical Sciences and Peking Union Medical College, Beijing 100021, China

## Abstract

Despite many changes in alternative splicing events (ASEs) are frequently involved in various cancers, prognosis-related ASEs and drug treatment targets in glioblastoma multiforme (GBM) have not been well explored. ASEs participate in many biological behaviors in the initiation and progression of tumors, the aberrant ASE has been considered another hallmark of cancer, and the systematic study of alternative splicing may provide potential biomarkers for malignancies. In this study, we carried out a systematic analysis to characterize the ASE signatures in GBM cohort. Through comparing GBM tissues and nontumor tissues, a total of 48,191 differently expressed ASEs from 10,727 genes were obtained, and these aberrant ASEs play an important role in the oncogenic process. Then, we identified 514 ASEs independently associated with patient survival in GBM by univariate and multivariate Cox regression, including exon skip in CD3D, alternate acceptor site in POLD2, and exon skip in DCN. Those prognostic models built on ASEs of each splice type can accurately predict the outcome of GBM patients, and values for the area under curve were 0.97 in the predictive model based on alternate acceptor site. In addition, the splicing-regulatory network revealed an interesting correlation between survival-associated splicing factors and prognostic ASE corresponding genes. Moreover, these three hub splicing factors in splicing regulation network are the potential targets of some drugs. In conclusion, a systematic analysis of ASE signatures in GBM could serve as an indicator for identifying novel prognostic biomarkers and guiding clinical treatment.

## 1. Introduction

Glioblastoma multiforme (GBM) is the most aggressive cancers in the central nervous system, and the 5-year overall survival rate of this disease is only 0.05% to 4.7% [1–3]. For GBM patients in China, the 1‐ and 5‐year overall survival (OS) rates are 61% and 9%, respectively [4]. Until now, standard therapy for GBM consists of surgical resection to a safe and feasible extent, followed by radiotherapy and adjuvant temozolomide, an alkylating agent [5]. Temozolomide is the only first-line drug in the treatment of recurrent glioma, and the levels of DNA repair gene O6-methylguanine DNA methyltransferase (MGMT) indicated sensitivity to the drug [6]. Median survival time for patients with methylated and unmethylated MGMT is 23 and 13 months, and the ratios of patients who survive more than 5 years in two groups are 14% and 8% separately [7]. Once to be a GBM patient, there are currently no effective curative options. Such limited treatment and poor outcome enable biomarkers of GBM, an ongoing research area, and need more exploration from scientists and oncologists.

Protein is the executor of life activities, and protein diversity is essential for the significant regulation and functional complexity of eukaryotic cells. Alternative splicing is a biological process leading to structural transcript changing and providing the possibility of diversity at the protein level [8, 9], so alternative splicing is a crucial system, and variation in splicing patterns is tightly associated with the function of proteins. Many alternative splicing events (ASEs) are closely related to biological activity, not only to physiological functions, such as cell development and differentiation [10], but also to pathological processes, including cancer-associated phenotype [11]. Alternative splicing is extensively perturbed in cancer, which produces transcripts with and without biological functions. Also, tumor progression is partially led by the cancer-specific ASE [9].

In recent decades, numerous genomic and functional studies have found that splicing defects and the production of specific isoforms are the drivers of cancer [12]. There is growing evidences proved that ASE plays an important role in oncogenic processes, such as cell proliferation, antiapoptosis [13], epithelial-mesenchymal transition (EMT) [14], metabolism, angiogenesis, immune escape, and metastasis [15–17]. In addition, it is proved that splicing factors (SFs) influence the site selection of splicing regulatory by combining pre-mRNA on the exon splicing enhancer or silencers [18] and changes in SFs are also associated with the initiation and progression of cancer [19]. Hence, it is also necessary to explore potential regulatory interactions between SFs and ASEs and further to seek the effect of abnormal ASEs on potential target drug therapeutic response.

Research studies on cancer-related ASEs have received more and more attention from researchers. With the development of high-throughput technology, RNA-seq data of clinical samples accumulate rapidly, and it is possible to study the alternative splicing to identified “cancer-specific” ASE and to explore the protein regulation network that ASE involved in. Recently, research studies on ASE in GBM are continually emerging [20, 21], and ASEs play an important role in GBM processes. For example, manipulation of MKNK2 alternative splicing by splice switching oligonucleotides is a novel approach to inhibit GBM cell proliferation and to enhance activity with chemotherapeutic drugs, which suggested a novel treatment strategy for clinical practice [22]. circSMARCA5 is an upstream regulator of pro- to antiangiogenic VEGFA alternative splicing isoform ratio within GBM cells, and a highly promising GBM prognostic and prospective antiangiogenic molecule could be a prognostic biomarker and a therapeutic target [23]. However, systematic analyses of alternative splicing in drugs therapeutic response in GBM have been lacking.

In our study, we systematically analyzed the ASE in GBM cohort. From The Cancer Genome Atlas (TCGA), exon, splice, and transcript isoform expression level datasets are available [24]. By comprehensive analysis, we identified a number of survival-related ASEs in GBM. Then, we constructed networks between prognosis-related ASEs and SFs and uncovered interesting splicing networks which could be underlying mechanisms. More importantly, we revealed that hub SFs were associated with drug therapeutic response. Through this method, we hope to find target markers to predict the prognosis and the therapeutic targets of GBM patients and further to guide clinical individualized treatment.

## 2. Materials and Methods

### 2.1. Data Curation from TCGA GBM

RNA-seq data, 450K methylation data, and copy number variation (CNV) data of TCGA GBM cohorts were downloaded from TCGA data portal (https://tcga-data.nci.nih.gov/tcga/). We used SpliceSeq tool [25], a Java program application, to analyze the splicing patterns of GBM samples from RNA-seq data. The percent-spliced-in (PSI) value, a common, intuitive ration for quantifying splicing events from zero to one [26], was computed for seven ASEs in each sample: alternate acceptor site (AA), alternate donor site (AD), alternate promoter (AP), alternate terminator (AT), exon skip (ES), mutually exclusive exons (MEs), and retained intron (RI). Annotation from GENCODE version 27 was used as the transcript model reference to guide the assembly process [27].

### 2.2. Identification and Enrichment Analysis of Different ASEs

To detect different ASEs, we used the Wilcoxon test to compare the PSI value distributions between GBM samples and adjacent normal samples. Multiple testing was corrected by using the Benjamini–Hochberg method to obtain the corrected *P* values. ASEs with adjusted *P* value < 0.05 and PSI |fold change| > 1.5 were identified as significant differences. To investigate the intersections between seven types of different ASEs, we applied UpSetR to visualize intersection sets and their aggregates, which is more scalable alternative to the traditional Venn diagram when addressing more than five sets [28].

In order to observe the biological functions involved in different ASEs and their roles in tumorigenesis, we performed the Gene Ontology (GO) enrichment analysis using R package “clusterProfiler.”

### 2.3. Survival Analysis

To analyze the association between ASE and OS of patients, we divided patients into two groups by the median PSI value of each splicing event and performed univariate Cox regression. Multivariate Cox regression was further conducted to determine splicing events that were independent prognostic factors and to build predictive models. The efficacy of predictive models which distinguish patients with various survival times was shown by Kaplan–Meier curves. The receiver operator characteristic (ROC) curves draw by R package “survivalROC” was further employed to evaluate the predictive model.

### 2.4. Construction of Network between Survival-Associated ASEs and SFs

To explore the regulation of SFs on prognostic ASEs, we collected 71 SFs from previous reports [29]. First, we curated TCGA level 3 mRNA-seq data of these SFs and determined the survival-associated SFs. The expression level of survival-associated SFs in GBM tissues and adjacent normal tissues was also compared. To assess the association between SFs and ASEs, we performed Matrix_eQTL_engine function in R package “MatrixEQTL,” and *P* value <  0.05 was considered to be significantly correlated. The regulation networks were plotted using Cytoscape (version 3.4.0).

### 2.5. Determination of Hub SFs Associated with Drug Therapeutic Response

The Genomics of Drug Sensitivity in Cancer (GDSC) project measured the responses of 1000 human cancer cell lines to a host of chemical drugs by the IC_50_ [30]. The value of IC50 greater than *μ* + standard deviation (SD) means that the cell line resists the drug, while this value less than *μ* − standard deviation (SD) indicates the cell line is sensitive to the drug. However, if this value is within *μ* ± SD, we thought the cell line to be intermediate and excluded it in further analysis. In the following analysis, we only considered drugs that had at least 3 resistant or 3 sensitive cell lines.

For each drug, Student's *t*-test was applied to identify the genes that differently expressed between resistant and sensitive cell lines. *P* value < 0.05 indicates that the expression of this gene was related to chemoresponse of the corresponding drugs.

### 2.6. Statistical Analysis

All statistical analysis and figure plotting in our study were performed using R software (http://www.r-project.org). Heatmaps and Circos plots were generated by the R packages “pheatmap” and “OmicCircos,” respectively. Additionally, statistical tests were two-sided, and a *P* value < 0.05 was considered statistically significant, unless indicated otherwise.

## 3. Results

### 3.1. The ASE Profile Landscape in GBM

**Figure 1:**
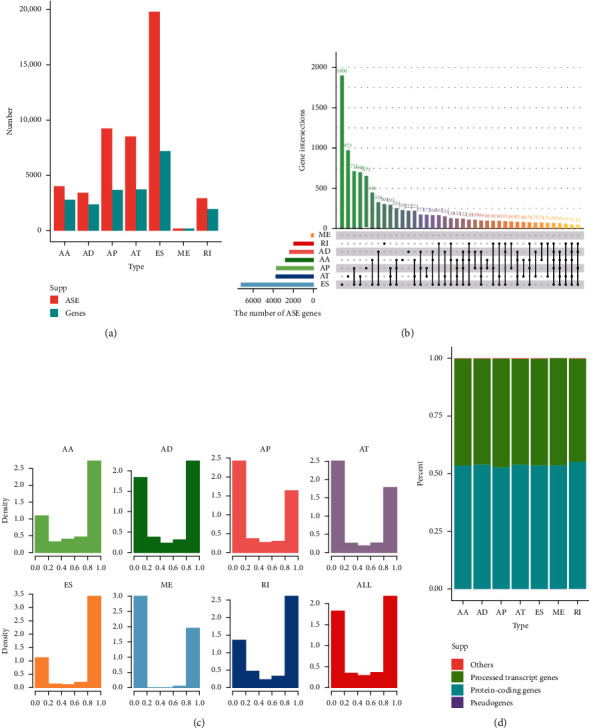
Overview of ASE profiling in GBM. (a) The number of ASEs and involved genes from the GBM patients was depicted according to the AS types. Salmon and cyan bars represent the preliminarily detected ASEs and involved genes, respectively. (b) UpSet plot of interactions between the seven types of detected ASEs in GBM. One gene may have up to five types of alternative splicing. (c) Bar plots demonstrate the fraction of every event of distinct PSI levels in different frequency ranges. AA, AD, AP, AT, ES, ME, RI, and ALL represent alternate acceptor site, alternate donor site, alternate promoter, alternate terminator, exon skip, mutually exclusive exons, retained intron, and all splicing types, respectively. (d) Bar plot of the proportion of four types of transcripts in different alternative splicing types.

To systematically characterize the human GBM ASE profiles, we collected 153 GBM samples and 5 adjacent normal samples from the TCGA. The GBM patients included 99 (64.7%) male and 54 (35.3%) female patients, among which 151 patients (98.7%) were untreated primary tumors. The median age of these patients was 60 (range, 21–89 years), and the median follow-up period after surgical resection was 357 days (range, 5–2681 days). The detailed characteristics of these patients are summarized in Table S1. In GBM cohort, we identified a total of 48,191 ASEs from 10,727 genes. According to their splicing pattern, these ASEs can be roughly divided into seven types, including 4029 AA events in 2796 genes, 3441 AD events in 2376 genes, 9248 AP events in 3693 genes, 8530 AT events in 3730 genes, 19,809 ES events in 7201 genes, 201 ME events in 196 genes, and 2933 RI events in 1953 genes, which are illustrated in Figure 1(a). We found that ES was the predominant type, since 41.1% of the ASEs were ES events and noticed that one gene might have two or more splicing types; as shown in Figure 1(b), the UpSet plot demonstrated that one gene might have up to five types of ASEs. For example, 50 genes had AA, AD, AT, ES, and RI events and 69 genes had AA, AD, AP, ES, and RI events, simultaneously.

We further explored the detective frequency of ASEs with distinct PSI levels in all samples. As a result, splicing events with low PSI levels (PSI ≤ 0.2) and high PSI levels (PSI > 0.8) constituted the majority of all types of ASEs (Figure 1(c)). In addition, according to GENCODE (v27), transcripts were categorized into four types of genes (Figure S1), including protein-coding genes, processed transcript genes, pseudogene, and others. In our GBM ASE, about half of the transcripts in every splicing events are classified as protein-coding genes (Figure 1(d)), indicating that not all transcripts could be translated into proteins, but nearly half of them may inevitably affect protein translation, modification, and regulatory functions.

### 3.2. The Biological Function of Aberrant ASE

**Figure 2:**
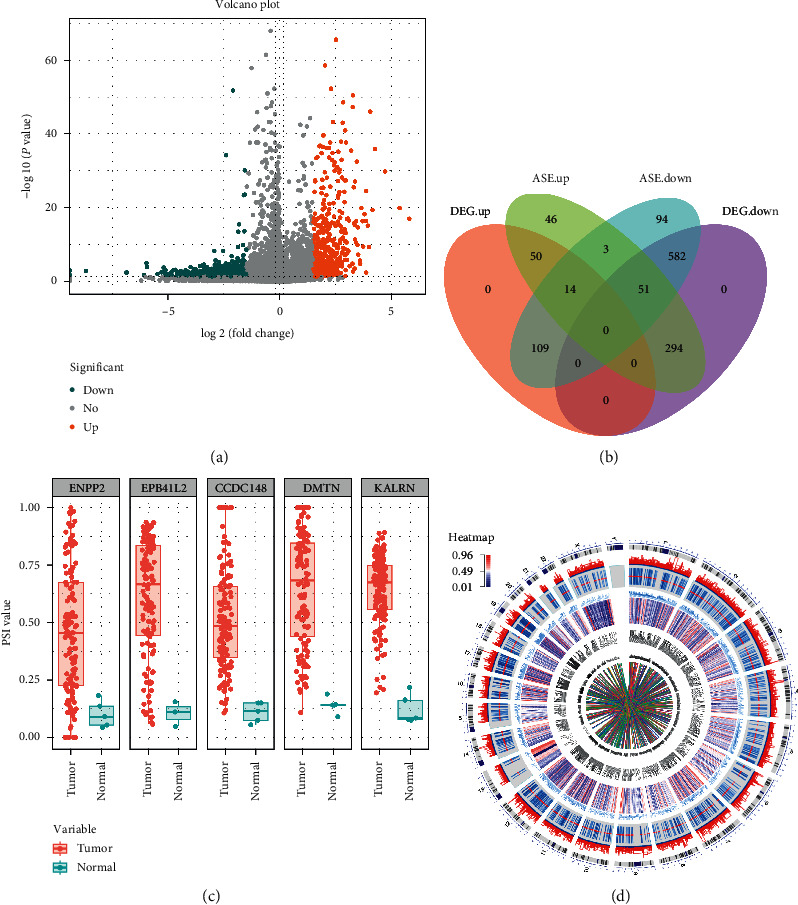
Identification of GBM-related aberrant ASEs. (a) Volcano plot visualized the aberrant ASE identified in GBM. The salmon and cyan points in the plot represent the differentially expressed alternative splicing with statistical significance, salmon represents upregulated ASE, and cyan represents downregulated ASE. (b) Venn diagram demonstrated the intersection set of aberrant ASE and DEG. (c) Scatter plot is overlaid with the boxplot about 5 representative ASE-related genes. (d) Circos plot displaying the distribution of aberrant ASE in gene expression, CNV, DNA methylation, and interactions between genes on chromosomes.

To fully describe the abnormal ASE that occurs in GBM, we identified different ASEs between tumor and adjacent normal samples. Through the Wilcoxon test, we identified 1555 ASEs in 1243 genes with the threshold of |log 2FC| > 1.5 and adjusted *P* value < 0.05, among which there are 29 AA events in 27 genes, 17 AD events in 16 genes, 333 AP events in 308 genes, 505 AT events in 475 genes, 639 ES events in 505 genes, 10 ME events in 10 genes, and 22 RI events in 21 genes. These different ASEs are shown in the volcano plot (Figure 2(a)) and Table S2, and all downregulated and upregulated ASEs are exhibited in the heatmap, respectively (Figures S2(a) and S2(b)). The detailed information of the top 10 upregulated and top 10 downregulated ASEs is listed in Table 1. In addition, we also noticed that one gene might have two or more events which were significantly different (Figure S2(c)) and the proportion of ASE between aberrant ASE and the entire ASE was consistent, and ES event was the predominant type.

The aberrant ASE may directly affect the expression of its corresponding genes, and in order to investigate the relationship between aberrant ASE and differently expressed genes (DEGs), we analyzed the aberrant ASE that occurred in DEG. A Venn diagram summarized the results (Figure 2(b)). The unique number of aberrant ASE in its corresponding DEG was 1243, and the number of DEG was 1100; as we expected, all differently expression genes were occurred aberrant ASE. Furthermore, for intuitively showing the difference of these ASEs, we generate graphs in which the scatter plot is overlaid with the boxplot about 5 representative ASE-related genes, for example, AP in ENPP2, ES in EPB41L2, AT in CCDC148, AP in DMTN, and AT in KALRN (Figure 2(c)). Considering all of these evidences, it suggested that, like GBM-related genes, GBM-related ASEs play a vital role in GBM biological and further research is needed.

To further explore the changes of abnormal ASEs in other omics, we examined the aberrant ASE and corresponding genes via epigenetic and CNV mechanisms (Figure 2(d)). Since there was only one normal sample in the GBM methylation data and no normal sample in the CNV data, we roughly observed the changes of the different ASEs in the two omics and concluded that the methylation and CNV level are also different, and the multiomics regulation requires further exploration.

**Figure 3:**
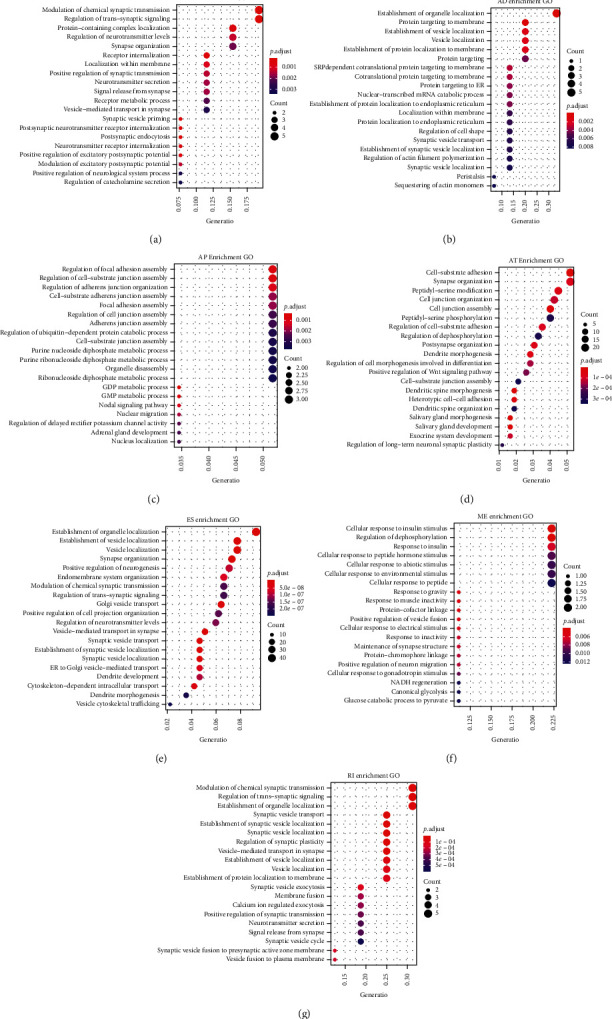
GO enrichment analyses on corresponding genes from 7 aberrant splicing types in GBM. (a–g) Top 20 pathways of GO term in biological process analyses of genes from aberrant AA events, AD events, AP events, AT events, ES events, ME events, and RI events, respectively. The dot size represents the enriched gene number, and FDR values are indicated by color scale by the side.

There was evidence that ASEs could directly affect the protein diversity and function through several mechanisms. Thus, we can shed light on the potential influence of the aberrant ASE by analyzing its corresponding protein. As shown in Figure 3, different splicing events have different functions. For example, the main function of different AP event corresponding genes was regulation of focal adhesion assembly; however, the different ES event corresponding genes were establishment of organelle localization. Taken together, above results indicated that the corresponding genes of aberrant ASEs play an important role in regulating the GBM-related biological process.

### 3.3. The Prognostic Predictor of Aberrant ASE in GBM

In order to investigate the relationship between aberrant ASE and GBM patient prognosis, we performed univariate Cox regression. In the result, we detected a total of 2512 survival-associated ASEs in GBM. The top 20 most significant survival-associated genes in seven types of ASEs are presented in Table 2. Among these prognostic ASEs, genes such as AA of B7H3 and ES of MAPKAP1 were included. In addition, we found that one gene might have two or more events which were significantly associated with patient survival; for example, ES, AP, and AA events in gene COPS3 were significantly associated with OS in GBM cohort.

**Figure 4:**
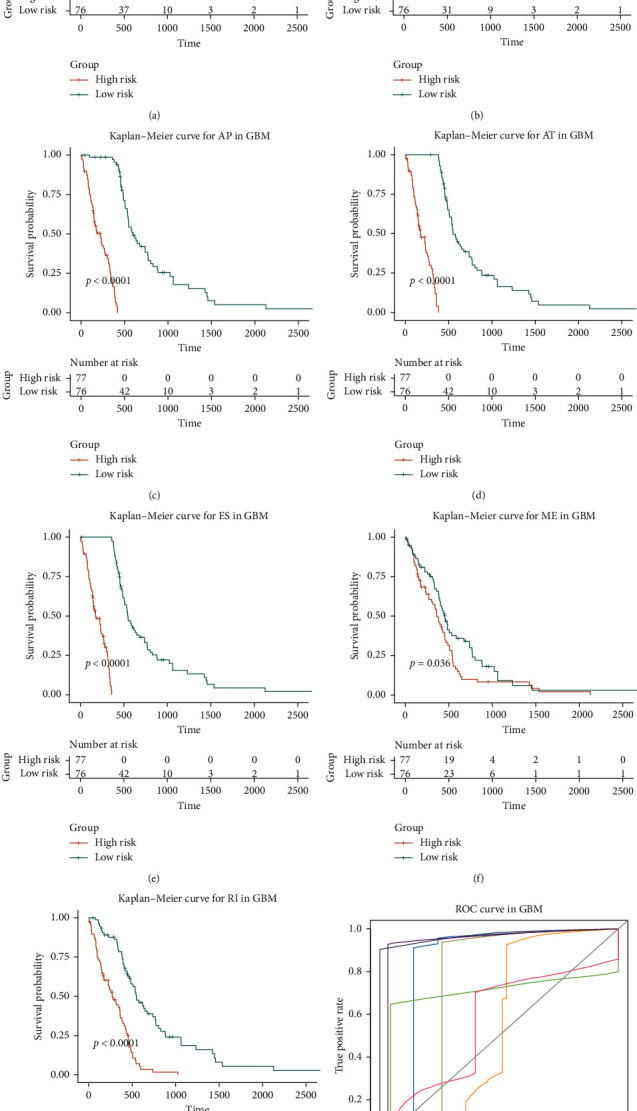
Kaplan–Meier plots and ROC curves of predictive factors in GBM patients. (a–g) Kaplan–Meier curves of prognostic models built with ASEs of AA, AD, AP, AT, ES, RI, and ME for patients with GBM. The salmon line indicates the high-risk group, whereas the cyan line indicates the low-risk group. (h) The ROC curves of predictive models for each splice type in GBM.

To choose independent prognostic factors, multivariate Cox regression was conducted to all of survival-associated ASEs to identify any events that might be an independent factor in GBM. In total, we identified 32 AA events, 13 AD events, 127 AP events, 161 AD events, 156 ES events, 5 ME events, and 20 RI events, and Kaplan–Meier curves show that every splice type performed reasonably well in distinguishing outcomes of patients with GBM (Figures 4(a)–4(g)). To further evaluate the efficiency of these splicing types, ROC curves were applied to each type. The area under curve (AUC) was obviously different among different splice type models, with the AUC values of 0.97 in the GBM AA type predictive model (Figure 4(h)), indicating that the AA splice type performed best in GBM in predicting patient survival.

### 3.4. Survival-Associated ASE Correlation Network of SFs

**Figure 5:**
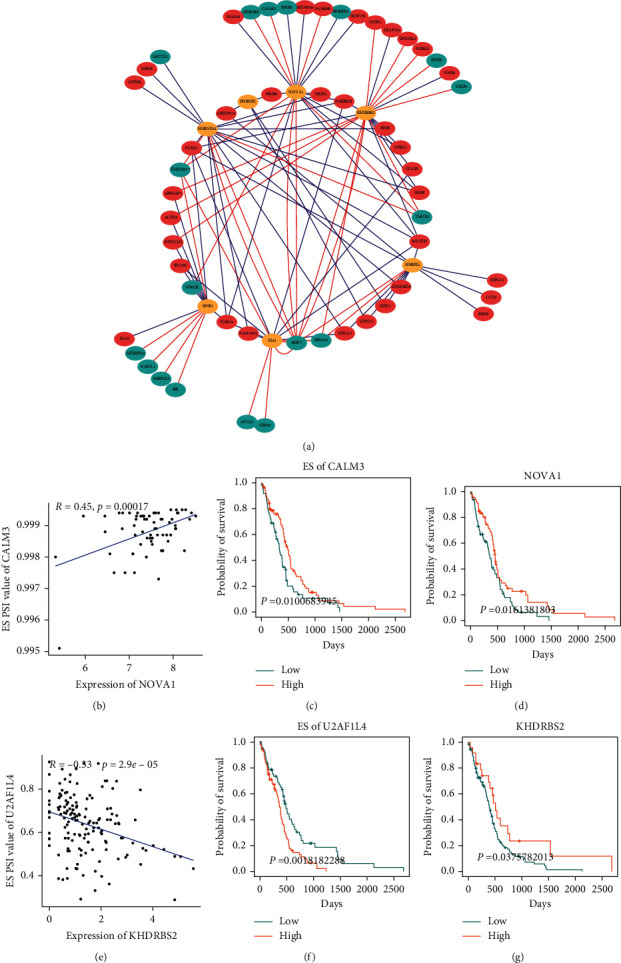
Construction of network between survival-associated ASEs and SFs. (a) Splicing correlation network in GBM constructed by Cytoscape. ASEs whose PSI values were positively/negatively correlated with survival times were represented with salmon/cyan dots. Yellow dots were survival-associated SFs. The positive/negative correlation between expressions of SFs and PSI values of alternative splicing is represented with salmon/cyan lines. (b) Dot plot of positive correlation between expression of NOVA1 and ES PSI values of CALM3. (c) Splicing type of ES corresponding gene CALM3 higher expressed indicated good survival. (d) High expression (salmon line) of splicing factor NOVA1 was significantly associated with good overall survival in GBM. (e) Dot plot of negative correlation between expression of KHDRBS2 and ES PSI values of U2AF1L4. (f) Splicing type of ES corresponding gene U2AF1L4 higher expressed indicated worse survival. (g) High expression (salmon line) of splicing factor KHDRBS2 was significantly associated with good overall survival in GBM.

Splicing factors are RNA-binding proteins that mediated pre-mRNA splicing with cis-sequence element and core spliceosome [31]. SFs are closely related to many genes that play an important role in tumorigenesis [9]. Thus, we further explored the network of interactions between independent prognostic-associated ASE corresponding genes and SFs. First, using level 3 mRNA expression levels of SFs from TCGA GBM cohort, we identified 9 SFs whose expression levels were significantly associated with patient survival, and all survival-associated SFs predicted a good prognosis (HR < 1). We also compared the expression level of these SFs in GBM tumor tissues and adjacent normal tissues and found that 7 factors were significantly dysregulated, including HNRNPA1, HNRNPC, HNRPLL, NOVA1, SF3B1, KHDRBS2, and TIA1. Then, we used Matrix eQTL engine function to evaluate the correlations between 7 SFs and independent survival-associated ASE and the correlation network was constructed using Cytoscape. In the correlation analysis, a total of 101 significant relational pairs and 54 significantly associated ASEs were identified, with 37 positive (red lines) and 64 negative (blue lines) correlations. The majority of favorable prognosis of these ASEs (cyan dots) was positively correlated (red lines) with expression of SFs (orange dots), where most adverse prognosis ASEs (salmon dots) were negatively correlated (blue lines) with expression of SFs (orange dots) (Figure 5(a)). This network suggested that one binding site can be targeted by different SFs, which partly explained why one gene can produce more than one transcript. Relationship between SFs and the specific ASE was exhibited in the dot plots. For example, splicing factor NOVA1 and ES of CALM3 were good predictors for GBM patients, and the expression of NOVA1 was positively correlated with ES of CALM3 (Figures 5(b)–5(d)); splicing factor KHDRBS2 was a good predictor for GBM patients, while ES of U2AF1L4 was a poor predictor, and the expression of KHDRBS2 was negatively correlated with ES of U2AF1L4 (Figures 5(e)–5(g)).

### 3.5. Identification of Hub SF-Associated Drug Response

**Figure 6:**
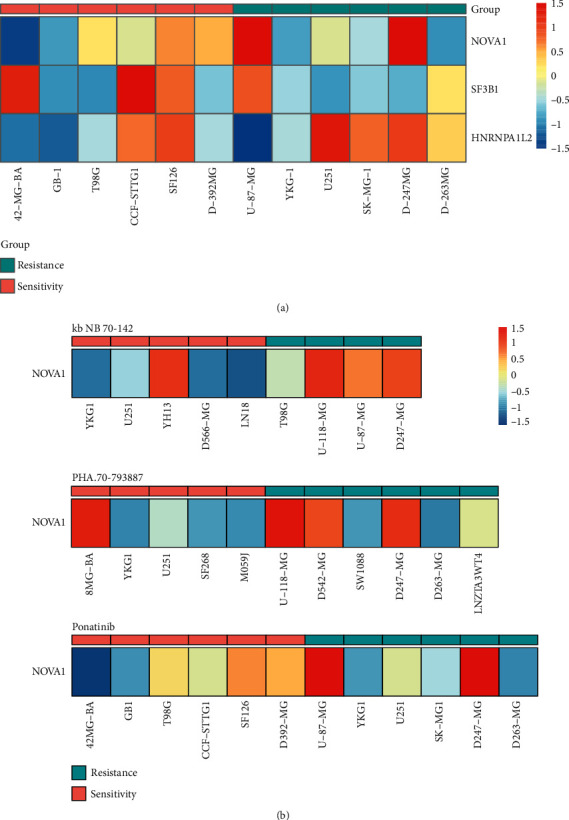
Hub SFs associated with drugs' chemoresponse. (a) The three SFs' expression in Ponatinib resistance and sensitivity cell lines. (b) The example of NOVA1 expression level in sensitive and resistant cell lines for three drugs, including Kb NB 142-70, PHA-793887, and ponatinib.

In the network of ASE and SFs, there are 7 SFs and 54 ASEs of 38 genes. Through calculating the degree of the SFs and genes, we found that splicing factor NOVA1 had the highest degree, followed by SFs SF3B1 and HNRNPA1. In order to evaluate the effect of the three SFs on drugs, we used GDSC data to evaluate whether these SFs' expression was related to chemoresponse. The three SFs associated with the significantly chemoresponse of drugs are shown in Table 3. In Figure 6(a), we showed the expression levels of three SFs in ponatinib-associated resistance and sensitivity cell lines. To illustrate, the expression level of NOVA1 was associated with three drugs' chemoresponse, including Kb NB 142-70, PHA-793887, and ponatinib (Figure 6(b)). In addition, some genes were associated with the same drug's chemoresponse, which might be potential drug targets for brain tumors, and these drugs may be expected to be used to treat brain tumors.

## 4. Discussion

We have systematically analyzed the interaction relationship between ASE and the prognosis of GBM patients. The method we applied to deeply analyze big datasets allowed us not only to identity prognosis-related ASEs but also to devise strategies for predicting the effectiveness of drug therapy in GBM.

Increasing evidence suggested that the ASEs were a posttranscriptional biological process, a predominant mechanism for RNA and protein diversity [32]. The specific dysregulation of splicing played critical roles in producing isoform to boost proliferation, cancer cell survival, drug resistance, and metastasis [12, 33, 34]. For instance, ES in FLNB has been reported to promote EMT in breast cancer by releasing the FOXC1 transcription factor and reducing FLNB nuclear localization [35]. Notably, scientists have found that high ECT2 splice variant including exon 5 (ECT2-Ex5+) levels was negatively related to prognosis in breast cancer treated with doxorubicin [36]. In short, aberrant ASEs play an important role in many biological processes, and these aberrant ASEs could serve as cancer hallmarkers or therapeutic targets in cancer treatment.

In this study, using the RNA-seq, methylation, and CNV datasets of TCGA GBM, we obtained a total of 48,191 ASEs from 10,727 genes, and only about half of the transcripts were the protein-coding genes, indicating that some transcripts do not encode proteins, but involved in the regulation of protein functions. We first systematically analyzed the different ASEs between GBM tissues and nontumor tissues and identified 1555 ASEs in 1243 genes, among which there are 579 upregulated ASEs in 459 genes and 976 downregulated ASEs in 850 genes. During which, ES was the predominant differentially spliced type, and we also found that one gene might have up to five types of ASEs. These results are similar to many previous studies about other cancers, such as lung cancer [37], ovarian cancer [38], and esophageal cancer [39].

In order to investigate the potential mechanism of abnormal ASEs, we performed GO enrichment analysis. For instance, the most important function of AA, AD, AP, AT, ES, ME, and RI was modulation of chemical synaptic transmission, establishment of organelle localization, regulation of focal adhesion assembly, cell-substrate adhesion, establishment of organelle localization, cellular response to insulin stimulus, and modulation of chemical synaptic transmission. These results indicated that abnormal ASEs involved in many biological processes, which were necessary for tumors.

Then, we utilized univariate and multivariate Cox regression and identified a total of 2512 survival-associated ASEs in patients with GBM, among which 514 ASEs were the independent prognosis factors. The predictive model of each splice type made a good distinction between patients with GBM. The AUC of predictive models constructed on each splice type was distinct, and the best one is the AA predictive model with an AUC value about 0.97. Such prognostic models could accurately stratify patients with different outcomes and thus promote precision medicine. To sum up, our survival analysis on ASE features expanded the scope of biomarkers for GBM.

In addition, the relationship between SFs and ASEs enables us to reveal the underlying mechanism of alternative splicing related to patient outcomes, and we focused on the independent prognosis-associated ASEs and their splicing correlation networks. Seven SFs were significantly related to patient survival in the GBM cohorts. Among them, HNRNPA1, HNRNPC, and HNRPLL are RNA-binding proteins, which belong to the heterogeneous nuclear ribonucleoproteins (hnRNPs) protein family and ubiquitously expressed to influence pre-mRNA processing [40]. There are some studies which have confirmed that hnRNPs are associated with tumor progression and patient survival [13, 41]. By reviewing the literature, we get that the NOVA1 could regulate telomerase in most types of cancer cells [42], the SF3b1 is associated with spliceosome assembly and therapeutic targeting of its cancer dysfunction [43], KHDRBS2 revealed frequent mutations in renal cell carcinoma [44], and TIA1 could regulate expression of VEGF producing more complexity to the angiogenic pattern of colorectal cancer [45].

Furthermore, we constructed the splicing regulation network between 7 SFs and 54 independent prognosis ASEs, indicating that SFs influence oncogenic processes by regulating the ASE. It is worth noting that the high expression of SFs was associated with good OS in GBM, and many poor prognostic-related ASEs were negatively associated with the expression of SFs in GBM. The splicing regulation network between SFs and ASEs unveiled the biological mechanisms underlying development and tumorigenesis and indicated that aberrant alternative splicing was regulated by upregulation of oncogenic SFs in GBM.

Given the high incidence of splicing defects in cancer, SF regulators represent a potentially promising new treatment strategy in cancer treatment [46]. To explore potential targets of small molecule modulators, we used GDSC data to assess the effect of the potential drugs of hub SFs. We found that splicing factor NOVA1 is the potential target of Kb NB 142 70, PHA.793887 and ponatinib. Ponatinib is an orally active multityrosine kinase inhibitor and approved by the US Food and Drug Administration for patients with chronic myeloid leukemia. Due to ponatinib's unique multitargeted characteristics, further studies have demonstrated its ability in other human malignancies, such as GBM [47]. Eudocia Q Lee et al. reported that they performed a phase II trial of ponatinib in patients with bevacizumab-refractory GBM and variants [48]. In addition, tyrosine-protein kinase that acts as a cell-surface receptor for VEGFA tyrosine-protein kinase inhibitors could reduce the VEGFA expression. NOVA1 acts as a splicing factor which could regulate the expression of VEGFA. However, the splicing regulation network is complex, and the research of new drugs requires much work. Further studies are needed to validate our conclusions and to clarify the underlying mechanisms of these findings.

Of course, the alternative splicing represents only one layer of biology, and these studies need to be integrated to other “omics,” such as methylomics, genomic, and proteomic. We should combine other omics date to conduct integrated analysis but limited by normal samples. In addition, the data of drug target analyses were collected from GDSC, which were assayed in human cell lines, not in vivo, so these results need further validation in vivo.

In summary, we identified different expression ASEs and illustrated that survival-associated ASEs can be a promising indicator of GBM patients' outcomes. The correlation networks between prognostic ASEs and SFs indicated a new latent mechanism in the progression of GBM and found potential drug targets of SFs. These comprehensive and in-depth analyses may provide insights to understand ASE-related mechanism in GBM initiation and progression, and reveal novel ASE-related hallmarks, potential treatment targets, and drugs, so as to guide clinical medication and individualized treatment.

## Figures and Tables

**Table 1:** The detailed information of the top 10 upregulated and top 10 downregulated ASEs.

Symbol	AS type	Exons	MeanT	MeanN	log FC	Adj.*P*
*Upregulated*						
CREM	ES	4 : 9.2 : 10.1 : 14	0.119	0.000	5.797	<0.01
CREM	ES	9.2 : 10.1 : 14	0.129	0.001	5.369	<0.01
TRPM3	AT	10.2	0.264	0.002	4.724	<0.01
KCNIP4	AP	4	0.557	0.008	4.268	<0.01
FRY	AP	54	0.275	0.004	4.154	<0.01
STX1A	AA	9.1	0.636	0.011	4.057	<0.01
VAMP2	AP	1	0.004	0.000	4.001	<0.01
PCSK1	AP	2	0.114	0.002	3.945	<0.01
IGSF3	ES	6	0.364	0.008	3.872	<0.01
GABARAPL1	ES	2.7 : 2.8 : 2.10 : 2.11 : 2.12 : 2.13 : 2.14	0.001	0.000	3.871	<0.01

*Downregulated*						
C1QTNF1	AP	5	0.000	0.561	−8.675	0.001
PALLD	AT	10	0.000	0.061	−6.864	0.004
CCDC53	ES	5	0.000	0.071	−6.849	0.004
FEZ2	AP	2	0.000	0.213	−6.080	0.017
KIF4A	AT	29	0.002	0.884	−5.963	<0.01
ZNF283	AT	8	0.002	0.626	−5.941	<0.01
SAR1B	ES	6	0.001	0.267	−5.476	0.010
DYNC2LI1	ES	2	0.002	0.323	−5.287	0.003
HAUS1	ES	3	0.004	0.653	−5.195	<0.01
IKBKB	AT	8	0.001	0.085	−5.134	0.005

MeanT: the mean PSI value in GBM tissues; MeanN: the mean PSI value in adjacent normal tissues; log FC: log 2 fold change. The Adj.*P* was calculated by the Wilcoxon test and adjusted through BH correction.

**Table 2:** The top 20 most significant survival-associated genes in seven splicing types.

ID	AS type	Symbol	*P* value
ID_18990	ES	CD3D	0.0001
ID_30765	AP	ZNF280D	0.0001
ID_1507	AP	SPOCD1	0.0001
ID_1508	AP	SPOCD1	0.0001
ID_46847	ES	PTPRS	0.0001
ID_30767	AP	ZNF280D	0.0001
ID_40976	AP	NKIRAS2	0.0001
ID_40977	AP	NKIRAS2	0.0001
ID_13845	ES	BRSK2	0.0001
ID_79475	AA	POLD2	0.0001
ID_28104	ES	ZFYVE26	0.0001
ID_32899	AP	PIGQ	0.0001
ID_17207	ES	RPS6KB2	0.0001
ID_70200	ES	PPA2	0.0001
ID_89054	ES	GRIPAP1	0.0001
ID_55511	AA	GTDC1	0.0001
ID_76557	AT	DST	0.0002
ID_23656	ES	DCN	0.0002
ID_20539	ES	EMP1	0.0002
ID_78886	AP	HDAC9	0.0002

**Table 3:** The three genes associated with different drugs' chemoresponse.

Gene Name	Drug name	Target
NOVA1	Kb NB 142-70	Protein kinase D inhibitors
	PHA.793887	Cyclin-dependent kinase inhibitors
	Ponatinib	Tyrosine kinase inhibitors

SF3B1	EHT-1864	GTPase inhibitor
	PIK-93	PI4K (PI4KIII*β*) inhibitor

HNRNPA1	Flavopiridol	Cyclin-dependent kinase inhibitors
	THZ-2-49	Cyclin-dependent kinase 9 inhibitor

## Data Availability

The data used to support the findings of this study are available from the corresponding author upon request.

## Supplementary Materials

Supplementary Materials Table S1: the detailed characteristics of the GBM patients. Table S2: the detailed information of all different ASEs. Figure S1: pie chart showing types and numbers of genes in the assembled transcriptome. Figure S2: identification of abnormal ASEs. (a, b) The heatmap exhibited all downregulated and upregulated ASE, respectively. (c) UpSet plot of interactions between the abnormal ASE in genes in GBM.
