# Supplementary material for: Systematic Profiling of mRNA Splicing Reveals the Prognostic Predictor and Potential Therapeutic Target for Glioblastoma Multiforme
Source: J Oncol. 2021 Jul 5;2021:4664955. doi: 10.1155/2021/4664955 (PMC8277521; doi:10.1155/2021/4664955)
Supplement: Supplementary Materials — Table S1: the detailed characteristics of the GBM patients. Table S2: the detailed information of all different ASEs. Figure S1: pie chart showing types and numbers of genes in the assembled transcriptome. Figure S2: identification of abnormal ASEs. (a, b) The heatmap exhibited all downregulated and upregulated ASE, respectively. (c) UpSet plot of interactions between the abnormal ASE in genes in GBM. [file 4664955.f1.docx]

Figure S1


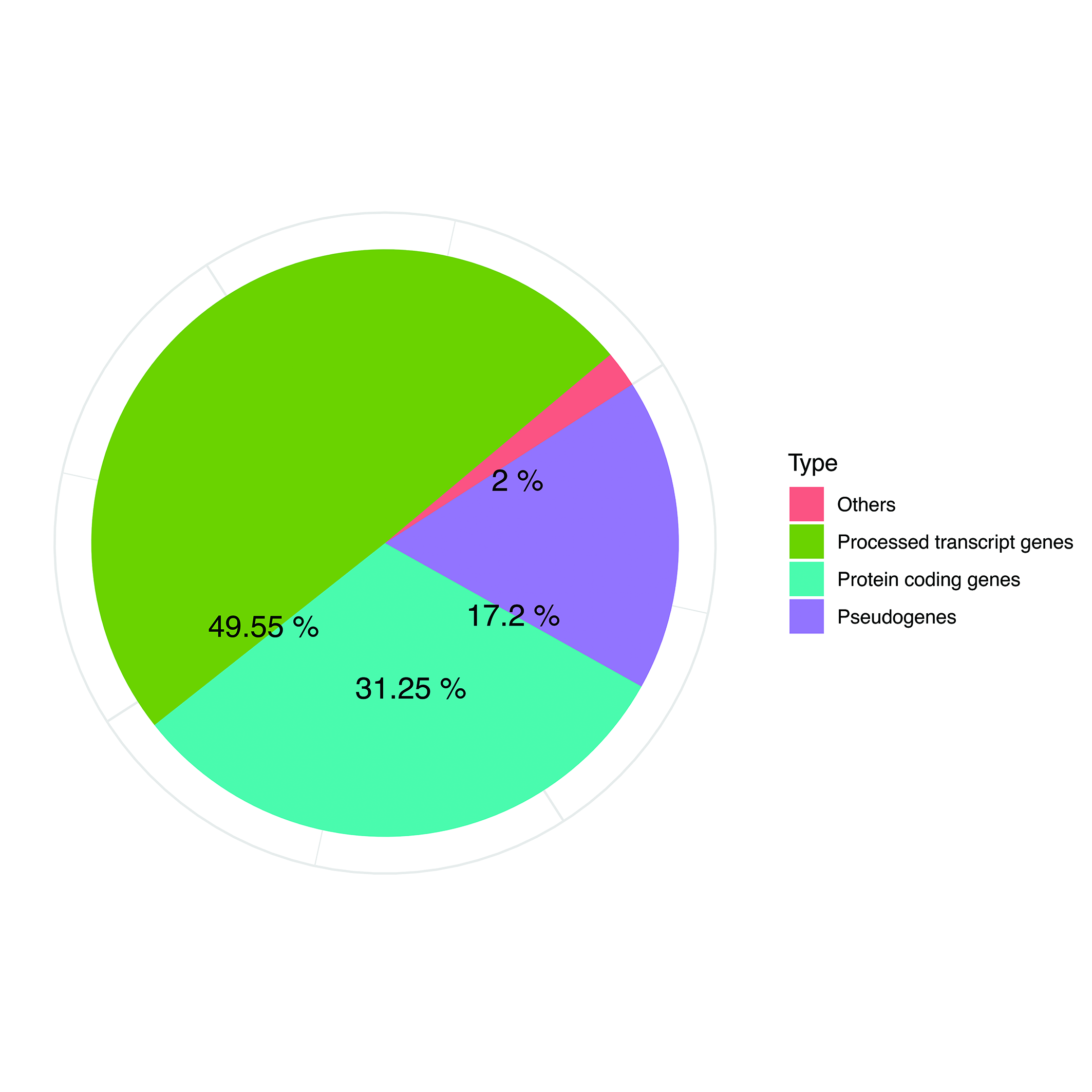


Figure S2


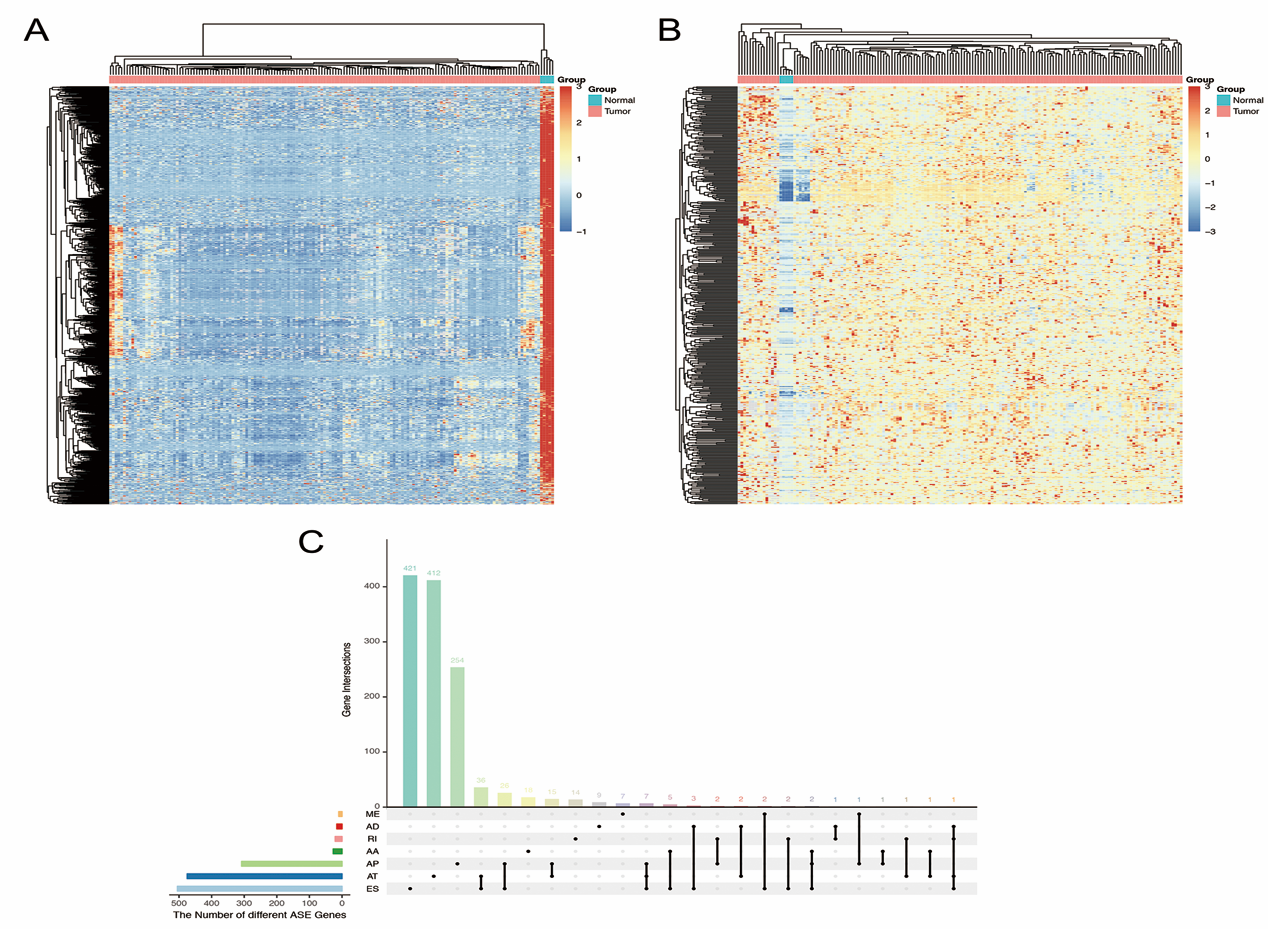


Table S1

|  | Vital_status | Time | gender | Age | radiationtherapy |
| --- | --- | --- | --- | --- | --- |
| TCGA-06-5408 | 1 | 357 | female | 54 | yes |
| TCGA-06-5410 | 1 | 108 | female | 72 | no |
| TCGA-06-5411 | 1 | 254 | male | 51 | yes |
| TCGA-06-5412 | 1 | 138 | female | 78 | yes |
| TCGA-06-5413 | 0 | 268 | male | 67 | yes |
| TCGA-06-5414 | 0 | 273 | male | 61 | yes |
| TCGA-06-5415 | 0 | 260 | male | 60 | yes |
| TCGA-06-5416 | 0 | 204 | female | 23 | yes |
| TCGA-06-5417 | 0 | 155 | female | 45 | yes |
| TCGA-06-5418 | 1 | 83 | female | 75 | no |
| TCGA-06-5856 | 1 | 114 | male | 58 | no |
| TCGA-06-5858 | 0 | 187 | female | 45 | yes |
| TCGA-06-5859 | 0 | 139 | male | 63 | yes |
| TCGA-12-1597 | 1 | 675 | female | 62 | yes |
| TCGA-12-3650 | 1 | 333 | male | 46 | yes |
| TCGA-12-3652 | 1 | 1062 | male | 60 | yes |
| TCGA-12-3653 | 1 | 442 | female | 34 | yes |
| TCGA-12-5295 | 1 | 454 | female | 60 | yes |
| TCGA-12-5299 | 1 | 98 | female | 56 | yes |
| TCGA-14-0781 | 1 | 29 | male | 49 | no |
| TCGA-14-0790 | 1 | 419 | female | 64 | yes |
| TCGA-14-1034 | 1 | 485 | female | 60 | yes |
| TCGA-14-1823 | 1 | 543 | female | 58 | yes |
| TCGA-14-1825 | 1 | 232 | male | 70 | yes |
| TCGA-14-1829 | 0 | 218 | male | 57 | yes |
| TCGA-14-2554 | 1 | 532 | female | 52 | yes |
| TCGA-16-1045 | 1 | 883 | female | 49 | yes |
| TCGA-19-1390 | 1 | 772 | female | 63 | no |
| TCGA-19-1787 | 1 | 385 | male | 48 | yes |
| TCGA-19-2619 | 0 | 294 | female | 55 | yes |
| TCGA-19-2620 | 1 | 148 | male | 70 | yes |
| TCGA-19-2624 | 1 | 5 | male | 51 | no |
| TCGA-19-2625 | 1 | 124 | female | 76 | no |
| TCGA-19-2629 | 1 | 737 | male | 60 | yes |
| TCGA-19-4065 | 0 | 214 | male | 36 | yes |
| TCGA-19-5960 | 1 | 455 | male | 56 | yes |
| TCGA-26-5132 | 0 | 286 | male | 74 | yes |
| TCGA-26-5135 | 1 | 270 | female | 72 | NA |
| TCGA-26-5136 | 1 | 577 | female | 78 | NA |
| TCGA-27-1830 | 1 | 154 | male | 57 | yes |
| TCGA-27-1831 | 1 | 505 | male | 66 | yes |
| TCGA-27-1832 | 1 | 300 | female | 59 | yes |
| TCGA-27-1834 | 1 | 1233 | male | 56 | yes |
| TCGA-27-1835 | 1 | 648 | female | 53 | yes |
| TCGA-27-1837 | 1 | 427 | male | 36 | yes |
| TCGA-27-2519 | 1 | 550 | male | 48 | yes |
| TCGA-27-2521 | 1 | 510 | male | 34 | yes |
| TCGA-27-2523 | 1 | 489 | male | 63 | yes |
| TCGA-27-2524 | 1 | 231 | male | 56 | yes |
| TCGA-27-2526 | 1 | 87 | female | 79 | no |
| TCGA-27-2528 | 1 | 480 | male | 62 | yes |
| TCGA-28-1747 | 1 | 77 | male | 44 | yes |
| TCGA-28-1753 | 0 | 37 | male | 53 | yes |
| TCGA-28-2509 | 0 | 145 | female | 77 | yes |
| TCGA-32-1970 | 1 | 468 | male | 59 | yes |
| TCGA-32-1980 | 1 | 36 | male | 72 | no |
| TCGA-32-1982 | 1 | 142 | female | 76 | NA |
| TCGA-32-2615 | 1 | 485 | male | 62 | yes |
| TCGA-32-2632 | 1 | 269 | male | 80 | NA |
| TCGA-32-2634 | 0 | 693 | male | 82 | no |
| TCGA-32-2638 | 1 | 766 | male | 67 | no |
| TCGA-32-4213 | 0 | 604 | female | 47 | yes |
| TCGA-32-5222 | 1 | 585 | male | 66 | yes |
| TCGA-41-2571 | 1 | 26 | male | 89 | no |
| TCGA-41-2572 | 1 | 406 | male | 67 | yes |
| TCGA-41-3915 | 1 | 360 | male | 48 | yes |
| TCGA-41-4097 | 1 | 6 | female | 63 | no |
| TCGA-41-5651 | 1 | 460 | female | 59 | yes |
| TCGA-76-4925 | 1 | 146 | male | 76 | yes |
| TCGA-76-4926 | 1 | 138 | male | 68 | yes |
| TCGA-76-4927 | 1 | 535 | male | 58 | NA |
| TCGA-76-4928 | 1 | 94 | female | 85 | yes |
| TCGA-76-4929 | 1 | 111 | female | 76 | NA |
| TCGA-76-4931 | 1 | 279 | female | 70 | yes |
| TCGA-76-4932 | 1 | 1458 | female | 50 | yes |
| TCGA-02-0047 | 1 | 448 | male | 78 | yes |
| TCGA-02-0055 | 1 | 76 | female | 62 | yes |
| TCGA-02-2483 | 0 | 466 | male | 43 | yes |
| TCGA-02-2485 | 0 | 470 | male | 53 | yes |
| TCGA-02-2486 | 1 | 618 | male | 64 | yes |
| TCGA-06-0125 | 1 | 1448 | female | 63 | yes |
| TCGA-06-0129 | 1 | 1024 | male | 30 | yes |
| TCGA-06-0130 | 1 | 394 | male | 54 | yes |
| TCGA-06-0132 | 1 | 771 | male | 49 | yes |
| TCGA-06-0138 | 1 | 737 | male | 43 | yes |
| TCGA-06-0139 | 1 | 362 | male | 40 | yes |
| TCGA-06-0141 | 1 | 313 | male | 62 | yes |
| TCGA-06-0156 | 1 | 178 | male | 57 | yes |
| TCGA-06-0157 | 1 | 97 | female | 63 | yes |
| TCGA-06-0158 | 1 | 329 | male | 73 | yes |
| TCGA-06-0168 | 1 | 598 | female | 59 | yes |
| TCGA-06-0174 | 1 | 98 | male | 54 | yes |
| TCGA-06-0178 | 1 | 2681 | male | 38 | yes |
| TCGA-06-0184 | 1 | 2126 | male | 63 | yes |
| TCGA-06-0187 | 1 | 828 | male | 69 | yes |
| TCGA-06-0190 | 1 | 317 | male | 62 | yes |
| TCGA-06-0210 | 1 | 225 | female | 72 | yes |
| TCGA-06-0211 | 1 | 360 | male | 47 | yes |
| TCGA-06-0219 | 1 | 22 | male | 67 | no |
| TCGA-06-0238 | 1 | 405 | male | 46 | yes |
| TCGA-06-0644 | 1 | 384 | male | 71 | yes |
| TCGA-06-0645 | 1 | 175 | female | 55 | no |
| TCGA-06-0646 | 1 | 175 | male | 60 | yes |
| TCGA-06-0649 | 1 | 64 | female | 73 | no |
| TCGA-06-0686 | 1 | 432 | male | 53 | yes |
| TCGA-06-0743 | 1 | 803 | male | 69 | yes |
| TCGA-06-0744 | 1 | 1426 | male | 66 | yes |
| TCGA-06-0745 | 1 | 239 | male | 59 | yes |
| TCGA-06-0747 | 1 | 82 | male | 53 | yes |
| TCGA-06-0749 | 1 | 82 | male | 50 | yes |
| TCGA-06-0750 | 1 | 28 | male | 43 | no |
| TCGA-06-0878 | 0 | 218 | male | 74 | yes |
| TCGA-06-0882 | 1 | 632 | male | 30 | yes |
| TCGA-06-1804 | 1 | 414 | female | 81 | no |
| TCGA-06-2557 | 1 | 33 | male | 76 | no |
| TCGA-06-2558 | 1 | 380 | female | 75 | yes |
| TCGA-06-2559 | 1 | 150 | male | 83 | yes |
| TCGA-06-2561 | 1 | 537 | female | 53 | yes |
| TCGA-06-2562 | 1 | 382 | male | 81 | yes |
| TCGA-06-2563 | 0 | 932 | female | 72 | yes |
| TCGA-06-2564 | 0 | 181 | male | 50 | yes |
| TCGA-06-2565 | 1 | 506 | male | 59 | yes |
| TCGA-06-2567 | 1 | 133 | male | 65 | yes |
| TCGA-06-2569 | 0 | 13 | female | 24 | NA |
| TCGA-06-2570 | 0 | 958 | female | 21 | yes |
| TCGA-08-0386 | 1 | 548 | male | 74 | yes |
| TCGA-12-0616 | 1 | 448 | female | 36 | yes |
| TCGA-12-0618 | 1 | 395 | male | 49 | yes |
| TCGA-12-0619 | 1 | 1062 | male | 60 | yes |
| TCGA-12-0821 | 1 | 323 | male | 62 | yes |
| TCGA-14-0787 | 1 | 68 | male | 69 | no |
| TCGA-14-0789 | 1 | 342 | male | 54 | yes |
| TCGA-14-0817 | 1 | 164 | female | 69 | no |
| TCGA-14-0871 | 1 | 880 | female | 74 | yes |
| TCGA-15-0742 | 1 | 419 | male | 65 | yes |
| TCGA-15-1444 | 1 | 1537 | male | 21 | yes |
| TCGA-16-0846 | 1 | 119 | male | 85 | no |
| TCGA-26-1442 | 0 | 953 | male | 43 | yes |
| TCGA-26-5133 | 0 | 452 | male | 59 | yes |
| TCGA-26-5134 | 0 | 167 | male | 74 | yes |
| TCGA-26-5139 | 0 | 48 | female | 65 | no |
| TCGA-28-2513 | 0 | 222 | female | 69 | yes |
| TCGA-28-2514 | 0 | 160 | male | 45 | yes |
| TCGA-28-5204 | 1 | 454 | male | 72 | yes |
| TCGA-28-5207 | 1 | 343 | male | 71 | yes |
| TCGA-28-5208 | 1 | 544 | male | 52 | yes |
| TCGA-28-5209 | 0 | 442 | female | 66 | yes |
| TCGA-28-5213 | 0 | 951 | male | 72 | yes |
| TCGA-28-5215 | 1 | 335 | female | 62 | yes |
| TCGA-28-5216 | 0 | 415 | male | 52 | yes |
| TCGA-28-5218 | 1 | 157 | male | 63 | yes |
| TCGA-28-5220 | 1 | 388 | male | 67 | yes |
| TCGA-32-2616 | 1 | 224 | female | 48 | yes |

Table S2

Table S2. The detailed information of all different ASE.

|  | | | | | | |  |
| --- | --- | --- | --- | --- | --- | --- | --- |
| symbol | as_id | splice_type | Pvalue | adjustPvalue | meanT | meanN | logFC |
| KIF21B | 9343 | AA | 2.23E-03 | 0.029961098 | 0.078957759 | 0.66202 | -2.126382759 |
| UNC93B1 | 17268 | AA | 0.000804269 | 0.014102661 | 0.051323026 | 0.23182 | -1.507821701 |
| FXYD6 | 18935 | AA | 0.004398918 | 0.04658185 | 0.094650388 | 0.54826 | -1.756559654 |
| NAP1L1 | 23482 | AA | 2.27E-03 | 0.030244745 | 0.001318065 | 0.0074 | -1.725315615 |
| DYNC2LI1 | 53400 | AA | 0.000253892 | 0.005862764 | 0.020197419 | 0.29312 | -2.67502724 |
| FAM13A | 69912 | AA | 5.04E-05 | 0.001700768 | 0.125723664 | 0.68516 | -1.695566031 |
| SSBP2 | 72673 | AA | 0.002990179 | 0.036449065 | 0.063092857 | 0.3213 | -1.627767702 |
| CDK5RAP2 | 87386 | AA | 0.000985357 | 0.016236102 | 0.11373252 | 0.52984 | -1.538725696 |
| ACADM | 3511 | AA | 0.004931157 | 0.049447293 | 0.013252258 | 0.00238 | 1.71706747 |
| BOLA1 | 7409 | AA | 8.44E-06 | 0.000419554 | 0.17249708 | 0.02158 | 2.078613351 |
| SNCG | 12440 | AA | 1.51E-08 | 1.83E-06 | 0.031269231 | 0.00676 | 1.531611681 |
| CALY | 13547 | AA | 3.26E-12 | 6.69E-10 | 0.234099123 | 0.04628 | 1.621034725 |
| PTMS | 19966 | AA | 7.11E-08 | 7.21E-06 | 0.035895484 | 0.00652 | 1.705737114 |
| EIF4B | 21920 | AA | 2.64E-06 | 0.000163245 | 0.00956129 | 0.00172 | 1.715398398 |
| DDIT3 | 22626 | AA | 0.002491507 | 0.03229111 | 0.044163448 | 0.0072 | 1.813816459 |
| C12orf76 | 24407 | AA | 4.20E-05 | 0.001479952 | 0.129934641 | 0.02648 | 1.590641828 |
| NPTN | 31602 | AA | 4.26E-05 | 0.001484696 | 0.906126452 | 0.18282 | 1.600676805 |
| BAIAP2 | 44099 | AA | 4.41E-08 | 4.82E-06 | 0.004428571 | 0.00028 | 2.761042731 |
| CNN2 | 46364 | AA | 0.002216405 | 0.029907788 | 0.017590323 | 0.00274 | 1.859390977 |
| TMEM91 | 50047 | AA | 8.26E-14 | 2.46E-11 | 0.453872656 | 0.022875 | 2.987772055 |
| TMEM91 | 50054 | AA | 1.47E-20 | 1.21E-17 | 0.557233824 | 0.02766 | 3.002997616 |
| TMEM91 | 50059 | AA | 5.56E-13 | 1.30E-10 | 0.311758904 | 0.0273 | 2.435343444 |
| SYNJ1 | 60373 | AA | 0.000518045 | 0.010171683 | 0.872673077 | 0.1323 | 1.886488932 |
| SEC14L2 | 61739 | AA | 0.001923927 | 0.02730977 | 0.016079221 | 0.00344 | 1.542056332 |
| RIOK1 | 75274 | AA | 0.002538211 | 0.032691072 | 0.075145033 | 0.0166 | 1.510017326 |
| STX1A | 80019 | AA | 5.51E-50 | 9.04E-47 | 0.635619333 | 0.011 | 4.056704579 |
| ACHE | 81027 | AA | 7.91E-53 | 2.59E-49 | 0.233791558 | 0.0137 | 2.83703411 |
| MORF4L2 | 89770 | AA | 6.29E-19 | 4.13E-16 | 0.202671756 | 0.01452 | 2.636060694 |
| NCAM1 | 102491 | AA | 7.31E-05 | 0.002218003 | 0.007779562 | 0.00082 | 2.249950983 |
| RPLP2 | 13770 | AD | 0.000340939 | 0.007034667 | 0.156648387 | 0.7417 | -1.554941128 |
| GRIA4 | 18552 | AD | 0.004419886 | 0.037465031 | 0.097987597 | 0.505 | -1.639717521 |
| RAB3IP | 23354 | AD | 0.001918447 | 0.02192341 | 0.016824503 | 0.12208 | -1.981840213 |
| SEC31A | 69735 | AD | 0.00031763 | 0.006681817 | 0.014127742 | 0.23038 | -2.791589742 |
| PTCD2 | 72469 | AD | 0.000248268 | 0.005503903 | 0.0277656 | 0.40294 | -2.674989823 |
| TMSB15B | 89790 | AD | 0.003828224 | 0.033739884 | 0.068008387 | 0.43078 | -1.845966482 |
| DUSP23 | 8411 | AD | 7.07E-05 | 0.002239871 | 0.089428244 | 0.008 | 2.413995022 |
| C15orf57 | 29999 | AD | 2.18E-07 | 1.74E-05 | 0.058682468 | 0.0016 | 3.602137374 |
| SUGP2 | 48548 | AD | 3.23E-34 | 9.31E-31 | 0.118038065 | 0.01016 | 2.452548711 |
| RPSA | 64185 | AD | 3.08E-08 | 4.05E-06 | 0.033523226 | 0.00524 | 1.855917008 |
| IP6K2 | 64761 | AD | 0.000274908 | 0.005957029 | 0.068201935 | 0.01216 | 1.724321067 |
| DLG1 | 68291 | AD | 0.000577705 | 0.009736524 | 0.002754545 | 0.00038 | 1.980836466 |
| ZSCAN25 | 80706 | AD | 5.73E-05 | 0.001989659 | 0.080101379 | 0.00798 | 2.306354662 |
| AP3M2 | 83568 | AD | 3.45E-17 | 2.49E-14 | 0.013963121 | 0.00084 | 2.810772996 |
| PTK2 | 85306 | AD | 0.00156493 | 0.01907182 | 0.007583871 | 0.00166 | 1.519206149 |
| SYP | 89107 | AD | 3.56E-14 | 1.71E-11 | 0.030709272 | 0.00546 | 1.727115823 |
| SYP | 89108 | AD | 5.03E-11 | 1.21E-08 | 0.010858278 | 0.00116 | 2.236507747 |
| MXRA8 | 142 | AP | 0.001362629 | 0.010134375 | 0.078377419 | 0.46906 | -1.789194825 |
| NPPA | 692 | AP | 0.001491967 | 0.010794354 | 0.079559589 | 0.55306 | -1.938960206 |
| UBR4 | 872 | AP | 0.000473308 | 0.004454899 | 0.003294805 | 0.31576 | -4.562635387 |
| MAN1C1 | 1192 | AP | 0.000490152 | 0.004569501 | 0.031332258 | 0.37382 | -2.479126222 |
| TMEM59 | 3108 | AP | 0.003958664 | 0.021276799 | 0.00761871 | 0.06386 | -2.126086166 |
| TGFBR3 | 3742 | AP | 0.001006515 | 0.008026498 | 0.002674286 | 0.15718 | -4.073709323 |
| C1orf194 | 3991 | AP | 0.008780431 | 0.037671028 | 0.031730667 | 0.31518 | -2.295860288 |
| ARHGEF2 | 8141 | AP | 0.004279554 | 0.022564828 | 0.013305921 | 0.07756 | -1.762842702 |
| CASQ1 | 8432 | AP | 1.56E-26 | 3.91E-24 | 0.180834228 | 0.89782 | -1.602388858 |
| MPC2 | 8878 | AP | 0.006723392 | 0.031395876 | 0.001783226 | 0.01772 | -2.29626997 |
| CRB1 | 9289 | AP | 0.009267239 | 0.038996103 | 0.027610714 | 0.18662 | -1.910870568 |
| TMEM9 | 9349 | AP | 0.000967844 | 0.007797515 | 0.007012258 | 0.07934 | -2.426082645 |
| CAPN2 | 9924 | AP | 0.000146707 | 0.001719855 | 0.018436129 | 0.1583 | -2.150179693 |
| COG2 | 10242 | AP | 0.00282721 | 0.016898602 | 0.001856129 | 0.02272 | -2.50475244 |
| PFKFB3 | 10692 | AP | 0.005434223 | 0.02689102 | 0.053312987 | 0.3601 | -1.91020181 |
| CELF2 | 10729 | AP | 4.22E-05 | 0.000605045 | 0.111765563 | 0.5647 | -1.619891127 |
| IFITM2 | 13641 | AP | 0.00147242 | 0.010746058 | 0.020153548 | 0.18298 | -2.205996486 |
| IFITM3 | 13647 | AP | 0.003208523 | 0.018447828 | 0.003005806 | 0.07478 | -3.214004568 |
| SLC25A22 | 13759 | AP | 0.005587212 | 0.027448846 | 0.022596732 | 0.3019 | -2.592290542 |
| TRIM34 | 14067 | AP | 0.000655709 | 0.00569275 | 0.139118033 | 0.78138 | -1.725738858 |
| DKK3 | 14408 | AP | 0.000116268 | 0.001422644 | 0.078179259 | 0.44704 | -1.743643691 |
| BTBD10 | 14447 | AP | 0.003832431 | 0.020798177 | 0.012557792 | 0.21968 | -2.861830576 |
| NUCB2 | 14519 | AP | 0.002568807 | 0.015765149 | 0.005730921 | 0.2484 | -3.76916409 |
| GTF2H1 | 14601 | AP | 0.001377126 | 0.010193077 | 0.040630323 | 0.43334 | -2.36700799 |
| CRY2 | 15512 | AP | 0.006175608 | 0.029452612 | 0.001002899 | 0.0317 | -3.453422323 |
| SLC25A45 | 16821 | AP | 2.48E-05 | 0.000388936 | 0.017667333 | 0.30164 | -2.837516893 |
| SERPINH1 | 17867 | AP | 0.007350321 | 0.033407059 | 0.024910968 | 0.13002 | -1.652380107 |
| SERPINH1 | 17868 | AP | 0.002728794 | 0.016526823 | 0.021304516 | 0.1126 | -1.664922641 |
| UVRAG | 17878 | AP | 0.00019728 | 0.002194457 | 0.0699312 | 0.36598 | -1.655066786 |
| TSKU | 17902 | AP | 5.81E-05 | 0.000801836 | 0.130683333 | 0.7844 | -1.792142 |
| FXYD6 | 18925 | AP | 0.000189673 | 0.00213716 | 0.010303226 | 0.08476 | -2.107366702 |
| ARHGEF12 | 19162 | AP | 0.002351855 | 0.014848088 | 0.016912766 | 0.37076 | -3.087486234 |
| SLC48A1 | 21359 | AP | 0.000393061 | 0.003842772 | 0.016549032 | 0.09726 | -1.771060179 |
| LIMA1 | 21689 | AP | 0.002502263 | 0.01551482 | 0.039816129 | 0.34292 | -2.153225102 |
| PDE1B | 22190 | AP | 3.59E-06 | 7.11E-05 | 0.194940179 | 0.91426 | -1.54542226 |
| SRGAP1 | 22854 | AP | 0.002375197 | 0.014925795 | 0.005947887 | 0.23492 | -3.676208946 |
| NAP1L1 | 23468 | AP | 0.000652082 | 0.005686518 | 0.016914194 | 0.17424 | -2.332280535 |
| PPP1CC | 24498 | AP | 0.000253617 | 0.002713217 | 0.006359355 | 0.09518 | -2.705842904 |
| PRKAB1 | 24704 | AP | 3.99E-05 | 0.000579186 | 0.034681046 | 0.44414 | -2.549946522 |
| TMED2 | 25082 | AP | 0.000244747 | 0.002642974 | 0.009756129 | 0.05618 | -1.750665115 |
| PGAM5 | 25290 | AP | 0.008846404 | 0.03787266 | 0.007988742 | 0.05808 | -1.983788105 |
| HOMEZ | 26723 | AP | 0.003734502 | 0.020436677 | 0.066752518 | 0.33486 | -1.612720515 |
| SEC23A | 27345 | AP | 0.003916218 | 0.021135713 | 0.070405195 | 0.322 | -1.520284495 |
| ALDH6A1 | 28367 | AP | 0.007169861 | 0.032862866 | 0.048316556 | 0.25206 | -1.651892872 |
| UNC79 | 29036 | AP | 0.008749443 | 0.037626319 | 0.049961379 | 0.44632 | -2.18978589 |
| EVL | 29240 | AP | 0.000794545 | 0.006640428 | 0.059774839 | 0.4064 | -1.916753081 |
| SNRPN | 29699 | AP | 1.94E-05 | 0.000316735 | 0.00282129 | 0.01314 | -1.538466672 |
| TRIM69 | 30364 | AP | 0.001764619 | 0.012228963 | 0.002046452 | 0.04002 | -2.973271956 |
| TPM1 | 30981 | AP | 0.003192569 | 0.018396624 | 0.012216129 | 0.06052 | -1.600216758 |
| SPG21 | 31149 | AP | 8.67E-06 | 0.000151835 | 0.081797419 | 0.59308 | -1.981083602 |
| C15orf61 | 31326 | AP | 0.009871019 | 0.040877817 | 0.057786452 | 0.34084 | -1.774658812 |
| CLN6 | 31358 | AP | 0.002046246 | 0.013625977 | 0.029838562 | 0.26112 | -2.169178485 |
| KIF23 | 31389 | AP | 8.26E-05 | 0.001080365 | 0 | 0.94855 | #NAME? |
| ARNT2 | 32182 | AP | 0.003830846 | 0.020798177 | 0.089447482 | 0.54108 | -1.799915482 |
| GPRC5B | 34341 | AP | 0.008521523 | 0.036970664 | 0.004896104 | 0.03348 | -1.92250848 |
| NUP93 | 36498 | AP | 0.012071457 | 0.047431803 | 0.005711842 | 0.04546 | -2.074291236 |
| IST1 | 37514 | AP | 0.003821745 | 0.020798177 | 0.019169032 | 0.10944 | -1.74208038 |
| CMIP | 37765 | AP | 5.71E-05 | 0.000793893 | 0.01168961 | 0.11676 | -2.3014201 |
| DBNDD1 | 38196 | AP | 2.35E-05 | 0.000371673 | 0.029837838 | 0.64178 | -3.068468252 |
| CTDNEP1 | 38876 | AP | 0.004691113 | 0.024041954 | 0.028813548 | 0.17172 | -1.785019537 |
| RPL23 | 40602 | AP | 0.000212443 | 0.002346464 | 0.003107742 | 0.01448 | -1.53887199 |
| THRA | 40837 | AP | 0.002148855 | 0.013988098 | 0.005463399 | 0.1721 | -3.450004636 |
| FAM134C | 41082 | AP | 0.001455204 | 0.010670137 | 0.008502614 | 0.1778 | -3.040285633 |
| PSME3 | 41143 | AP | 0.000255051 | 0.002721125 | 0.014017419 | 0.15694 | -2.415562771 |
| CRHR1 | 41978 | AP | 0.001546789 | 0.011112757 | 0.025330323 | 0.19894 | -2.061001073 |
| SPOP | 42303 | AP | 0.000540225 | 0.004948053 | 0.010449032 | 0.14752 | -2.647454393 |
| SEC14L1 | 43705 | AP | 0.001100684 | 0.008636729 | 0.011263871 | 0.17744 | -2.75703218 |
| C1QTNF1 | 43983 | AP | 0.000114301 | 0.001409701 | 9.59E-05 | 0.56118 | -8.674591002 |
| TGIF1 | 44501 | AP | 0.003046049 | 0.017846792 | 0.006379355 | 0.07326 | -2.440947788 |
| DLGAP1 | 44516 | AP | 7.53E-07 | 1.75E-05 | 0.081544526 | 0.44056 | -1.686897448 |
| NOL4 | 45074 | AP | 0.002995175 | 0.017661048 | 0.004051429 | 0.07558 | -2.926122145 |
| NEDD4L | 45650 | AP | 0.00148997 | 0.010794354 | 0.069191304 | 0.59792 | -2.156581771 |
| SEMA6B | 46810 | AP | 0.006898801 | 0.031967166 | 0.029031387 | 0.6502 | -3.108902458 |
| EPS15L1 | 48152 | AP | 1.89E-05 | 0.000309626 | 0.029756164 | 0.4191 | -2.645073238 |
| CCDC124 | 48386 | AP | 0.000603039 | 0.005360266 | 0.030143871 | 0.21226 | -1.95183032 |
| KXD1 | 48455 | AP | 0.002545636 | 0.0156623 | 0.001713548 | 0.04184 | -3.195286519 |
| ZNF85 | 48732 | AP | 0.000688933 | 0.005883385 | 0.054931333 | 0.45986 | -2.124838175 |
| ZNF100 | 48786 | AP | 0.000790521 | 0.006613859 | 0.00172069 | 0.17034 | -4.595071267 |
| PSMD8 | 49637 | AP | 0.012056582 | 0.047431803 | 0.002247097 | 0.01164 | -1.644808382 |
| PRR19 | 50149 | AP | 0.002497099 | 0.015495073 | 0.129674 | 0.59866 | -1.529670216 |
| PRMT1 | 51039 | AP | 0.001658657 | 0.011650864 | 0.002323871 | 0.02248 | -2.269391709 |
| SH3YL1 | 52493 | AP | 0.011541582 | 0.045972599 | 0.0127 | 0.06348 | -1.609122902 |
| PDIA6 | 52666 | AP | 0.007446553 | 0.033688016 | 0.000462581 | 0.02266 | -3.891535625 |
| FEZ2 | 53195 | AP | 0.002764232 | 0.016689821 | 0.000487097 | 0.21284 | -6.07983317 |
| MTA3 | 53364 | AP | 0.002926367 | 0.017334632 | 0.120307432 | 0.5952 | -1.59884708 |
| RTN4 | 53582 | AP | 0.00892244 | 0.038114322 | 0.159235484 | 0.74424 | -1.541979426 |
| SPRED2 | 53802 | AP | 0.002376768 | 0.014925795 | 0.016802581 | 0.2676 | -2.767960844 |
| CTNNA2 | 54168 | AP | 4.32E-06 | 8.36E-05 | 0.002748993 | 0.04276 | -2.744368318 |
| VAMP8 | 54290 | AP | 0.006151046 | 0.029389163 | 0.049718831 | 0.32324 | -1.872011323 |
| PLEKHB2 | 55366 | AP | 0.001217439 | 0.009265081 | 0.047083226 | 0.30872 | -1.88051792 |
| GTDC1 | 55498 | AP | 0.005533077 | 0.027285595 | 0.024561871 | 0.12074 | -1.592444212 |
| PDE1A | 56453 | AP | 0.0033249 | 0.018881284 | 0.024157031 | 0.31572 | -2.570280258 |
| SATB2 | 56716 | AP | 0.00177013 | 0.012256309 | 0.027468182 | 0.26694 | -2.273995604 |
| SP140L | 57884 | AP | 0.012794331 | 0.049428915 | 0.00455082 | 0.471175 | -4.63992221 |
| CBFA2T2 | 58986 | AP | 0.000134134 | 0.001599942 | 0.038812987 | 0.20656 | -1.67183602 |
| SLC12A5 | 59653 | AP | 0.010309717 | 0.042266358 | 0.001142 | 0.02364 | -3.030159081 |
| SLC12A5 | 59654 | AP | 0.001628925 | 0.011538997 | 0.066950667 | 0.53354 | -2.075578015 |
| TSHZ2 | 59816 | AP | 0.007565577 | 0.034029886 | 0.011852991 | 0.5699 | -3.872880628 |
| RCAN1 | 60491 | AP | 0.00044378 | 0.004222649 | 0.008532903 | 0.25676 | -3.404212138 |
| TBC1D10A | 61714 | AP | 0.000584498 | 0.005261153 | 0.003783007 | 0.15466 | -3.710710086 |
| SUN2 | 62257 | AP | 0.009640082 | 0.040135744 | 0.040328671 | 0.30708 | -2.030045637 |
| TBC1D22A | 62720 | AP | 0.000339077 | 0.003430639 | 0.025715584 | 0.22094 | -2.150793966 |
| TBC1D5 | 63658 | AP | 0.003578773 | 0.01987615 | 0.015107042 | 0.11488 | -2.028727096 |
| SATB1 | 63670 | AP | 0.007378516 | 0.033476917 | 0.003057792 | 0.04924 | -2.779013146 |
| CLASP2 | 63870 | AP | 2.39E-05 | 0.000376028 | 0.002744667 | 0.02004 | -1.988070642 |
| MAP4 | 64546 | AP | 0.001187515 | 0.009090353 | 0.016008387 | 0.15108 | -2.244696719 |
| C3orf18 | 65061 | AP | 0.012066443 | 0.047431803 | 0.002947403 | 0.08882 | -3.405687543 |
| ARHGEF3 | 65362 | AP | 0.001122624 | 0.00876497 | 0.0351375 | 0.23282 | -1.890996687 |
| PDZRN3 | 65629 | AP | 0.00220637 | 0.014175034 | 0.047016327 | 0.65444 | -2.633284994 |
| ROBO2 | 65640 | AP | 0.004115357 | 0.021893636 | 0.062613514 | 0.60008 | -2.260081854 |
| SLC35A5 | 66110 | AP | 0.002017526 | 0.013526753 | 0.003178571 | 0.12052 | -3.635383855 |
| SEC22A | 66462 | AP | 0.001985038 | 0.013377843 | 0.038796078 | 0.40382 | -2.342650064 |
| SELT | 67289 | AP | 0.004453057 | 0.023232436 | 0.000396129 | 0.00458 | -2.44771428 |
| MCCC1 | 67770 | AP | 0.001706478 | 0.011901447 | 0.034056129 | 0.2906 | -2.143937734 |
| ECE2 | 67857 | AP | 0.004822604 | 0.024619171 | 0.165553247 | 0.75836 | -1.521865331 |
| WHSC1 | 68522 | AP | 0.000198836 | 0.002205501 | 0.026796403 | 0.3918 | -2.682483848 |
| RNF4 | 68566 | AP | 0.000291041 | 0.003034805 | 0.002719481 | 0.0809 | -3.392772947 |
| LCP2 | 74488 | AP | 0.00021566 | 0.002375291 | 0.049850323 | 0.4832 | -2.271405679 |
| RPL26L1 | 74567 | AP | 0.001227571 | 0.009333112 | 0.031496129 | 0.22804 | -1.979656402 |
| JARID2 | 75409 | AP | 0.012939229 | 0.049755573 | 0.02455 | 0.23064 | -2.240146199 |
| BAI3 | 76620 | AP | 0.003674515 | 0.020207252 | 0.01208069 | 0.0745 | -1.819190844 |
| PGM3 | 76860 | AP | 7.13E-07 | 1.70E-05 | 0.018981169 | 0.13796 | -1.983516415 |
| DYNLT1 | 78273 | AP | 0.000227974 | 0.002489906 | 0.00712129 | 0.15462 | -3.07788156 |
| PSMG3 | 78589 | AP | 0.000232533 | 0.002536167 | 0.03198129 | 0.18654 | -1.763494639 |
| FOXK1 | 78641 | AP | 5.07E-05 | 0.000710846 | 0.004234899 | 0.45266 | -4.671781735 |
| ARL4A | 78821 | AP | 0.001368696 | 0.010149869 | 0.031896711 | 0.14742 | -1.53078277 |
| ING3 | 81583 | AP | 0.00018651 | 0.002106966 | 0.022135484 | 0.13538 | -1.810903713 |
| KCNH2 | 82271 | AP | 0.004257289 | 0.022480667 | 0.141495364 | 0.73784 | -1.651460044 |
| PRKAG2 | 82386 | AP | 0.000345169 | 0.00346986 | 0.031399338 | 0.27776 | -2.17997063 |
| TNKS | 82594 | AP | 0.001064472 | 0.008394643 | 0.060099355 | 0.48882 | -2.095995217 |
| UBE2V2 | 83796 | AP | 0.000627699 | 0.005524249 | 0.006089677 | 0.04592 | -2.020305641 |
| NCALD | 84752 | AP | 0.009690089 | 0.040277646 | 0.001851316 | 0.01556 | -2.128806895 |
| NCALD | 84755 | AP | 0.009880516 | 0.040895519 | 0.053928947 | 0.27686 | -1.635844573 |
| OXR1 | 84843 | AP | 0.010876933 | 0.044064801 | 0.081951299 | 0.4771 | -1.76160096 |
| SYBU | 84906 | AP | 0.000251398 | 0.002700548 | 0.118301342 | 0.55472 | -1.545228365 |
| S1PR3 | 86791 | AP | 0.000532217 | 0.004897521 | 0.057152258 | 0.39812 | -1.941034567 |
| SLC44A1 | 87110 | AP | 0.011585807 | 0.046078445 | 0.03780596 | 0.1718 | -1.51386424 |
| PHF19 | 87400 | AP | 0.002293767 | 0.014568262 | 0.039617763 | 0.31328 | -2.067819777 |
| SH2D3C | 87661 | AP | 0.004770721 | 0.024386106 | 0.117876224 | 0.70994 | -1.795545337 |
| C9orf116 | 88112 | AP | 0.00066638 | 0.005747161 | 0.04615 | 0.2618 | -1.735683893 |
| EGFL7 | 88188 | AP | 6.81E-06 | 0.000123493 | 0.123794156 | 0.58098 | -1.54609618 |
| PHPT1 | 88223 | AP | 0.005123913 | 0.025770944 | 0.011253289 | 0.50572 | -3.805322673 |
| MCTS1 | 90007 | AP | 0.006284861 | 0.029908194 | 0.054051613 | 0.31444 | -1.760853894 |
| AIFM1 | 90068 | AP | 0.003356144 | 0.01901753 | 0.057323871 | 0.31056 | -1.689659986 |
| VMA21 | 90365 | AP | 0.007333467 | 0.033349814 | 0.011042581 | 0.0957 | -2.159459531 |
| CTXN2 | 93676 | AP | 0.001629694 | 0.011538997 | 0.033324286 | 0.21432 | -1.861183793 |
| SULT1A3 | 94133 | AP | 0.009965535 | 0.041138697 | 0.000714194 | 0.01144 | -2.773717263 |
| KCNAB2 | 361 | AP | 4.15E-55 | 3.25E-51 | 0.484332 | 0.01828 | 3.276963056 |
| PLEKHG5 | 465 | AP | 2.39E-05 | 0.000376028 | 0.263937037 | 0.05292 | 1.606929239 |
| NBL1 | 911 | AP | 1.15E-06 | 2.51E-05 | 0.02918 | 0.00198 | 2.690386698 |
| KIAA1522 | 1632 | AP | 2.21E-30 | 6.65E-28 | 0.55382 | 0.04092 | 2.605220783 |
| RNF220 | 2553 | AP | 5.59E-07 | 1.36E-05 | 0.089692903 | 0.01638 | 1.700330571 |
| NFIA | 3223 | AP | 8.56E-07 | 1.93E-05 | 0.228418831 | 0.038 | 1.793594763 |
| PRKACB | 3585 | AP | 2.61E-41 | 2.05E-38 | 0.184210968 | 0.00944 | 2.971125684 |
| AHCYL1 | 4088 | AP | 2.75E-07 | 6.89E-06 | 0.396424516 | 0.05156 | 2.039739469 |
| ATP1A1 | 4358 | AP | 8.63E-31 | 2.82E-28 | 0.048575325 | 0.00724 | 1.903494473 |
| PDE4DIP | 4405 | AP | 7.42E-07 | 1.74E-05 | 0.008196129 | 0.00164 | 1.608965732 |
| PDE4DIP | 4408 | AP | 6.90E-13 | 4.07E-11 | 0.048122581 | 0.01038 | 1.533870641 |
| RUSC1 | 8078 | AP | 3.16E-06 | 6.32E-05 | 0.489133766 | 0.10598 | 1.529385606 |
| CASQ1 | 8433 | AP | 1.56E-26 | 3.91E-24 | 0.819165772 | 0.10218 | 2.081550507 |
| TSTD1 | 8523 | AP | 0.008985052 | 0.038281796 | 0.028578621 | 0.0056 | 1.629892312 |
| PBX1 | 8783 | AP | 0.001364019 | 0.010134375 | 0.028039355 | 0.0042 | 1.898524528 |
| SMYD3 | 10488 | AP | 6.26E-09 | 2.11E-07 | 0.132179355 | 0.01492 | 2.181457154 |
| MGEA5 | 12915 | AP | 3.08E-08 | 9.27E-07 | 0.030596026 | 0.00306 | 2.302455231 |
| INPP5F | 13271 | AP | 1.86E-31 | 6.93E-29 | 0.415321918 | 0.02586 | 2.776356552 |
| INPP5F | 13272 | AP | 4.01E-06 | 7.81E-05 | 0.037839726 | 0.00232 | 2.791792318 |
| LRRC27 | 13489 | AP | 3.26E-05 | 0.000487502 | 0.176076712 | 0.03824 | 1.527037677 |
| CHID1 | 13803 | AP | 1.36E-18 | 1.75E-16 | 0.092709032 | 0.00334 | 3.323495096 |
| RHOG | 14026 | AP | 0.000905176 | 0.007383783 | 0.003444805 | 0.00026 | 2.583941004 |
| RIC3 | 14218 | AP | 5.13E-10 | 2.06E-08 | 0.076664748 | 0.00476 | 2.779194328 |
| DGKZ | 15541 | AP | 2.23E-17 | 2.39E-15 | 0.290236842 | 0.04478 | 1.868935675 |
| BSCL2 | 16400 | AP | 1.93E-07 | 4.99E-06 | 0.267708387 | 0.02968 | 2.199424862 |
| BSCL2 | 16403 | AP | 0.001328066 | 0.009933223 | 0.259581935 | 0.05346 | 1.580138686 |
| DLG2 | 18095 | AP | 1.53E-14 | 1.20E-12 | 0.173446296 | 0.01034 | 2.819848151 |
| DIXDC1 | 18706 | AP | 7.58E-13 | 4.43E-11 | 0.164090066 | 0.0276 | 1.782599689 |
| FXYD6 | 18924 | AP | 7.43E-06 | 0.000132845 | 0.011745161 | 0.00224 | 1.656965485 |
| SORL1 | 19183 | AP | 0.006072072 | 0.029100608 | 0.037365574 | 0.00676 | 1.709726903 |
| NINJ2 | 19604 | AP | 1.94E-05 | 0.000316735 | 0.319544371 | 0.02456 | 2.565777037 |
| CLSTN3 | 20081 | AP | 1.96E-16 | 1.85E-14 | 0.070369677 | 0.0105 | 1.902387195 |
| MANSC1 | 20502 | AP | 0.004605799 | 0.023682212 | 0.07174106 | 0.01244 | 1.752146155 |
| PTPRO | 20573 | AP | 2.17E-36 | 9.99E-34 | 0.482485417 | 0.03034 | 2.766483722 |
| BCAT1 | 20755 | AP | 1.21E-18 | 1.64E-16 | 0.241209459 | 0.02856 | 2.133658545 |
| CCDC91 | 20915 | AP | 0.009277919 | 0.039020077 | 0.010116883 | 0.00198 | 1.631108779 |
| CACNB3 | 21468 | AP | 3.63E-21 | 6.17E-19 | 0.064126712 | 0.00144 | 3.796217893 |
| CSAD | 21943 | AP | 1.22E-09 | 4.58E-08 | 0.133070833 | 0.02648 | 1.614491837 |
| ZNF385A | 22177 | AP | 6.26E-10 | 2.49E-08 | 0.38829281 | 0.07642 | 1.625515279 |
| PDE1B | 22191 | AP | 3.76E-06 | 7.37E-05 | 0.801245536 | 0.08426 | 2.25226018 |
| SHMT2 | 22538 | AP | 0.0016496 | 0.011642179 | 0.05627871 | 0.01202 | 1.543744376 |
| AGAP2 | 22721 | AP | 3.26E-19 | 4.73E-17 | 0.143464516 | 0.00352 | 3.707626741 |
| FAM19A2 | 22821 | AP | 3.88E-05 | 0.000564951 | 0.569121622 | 0.06944 | 2.103631087 |
| RIC8B | 24156 | AP | 1.50E-22 | 2.87E-20 | 0.217093464 | 0.03156 | 1.928437475 |
| UNG | 24277 | AP | 1.10E-17 | 1.23E-15 | 0.276369032 | 0.0178 | 2.742538589 |
| AACS | 25172 | AP | 1.98E-05 | 0.00032185 | 0.007976159 | 0.00028 | 3.349422636 |
| FRY | 25582 | AP | 2.08E-22 | 3.88E-20 | 0.275196522 | 0.00432 | 4.154230065 |
| RTN1 | 27756 | AP | 2.60E-20 | 4.16E-18 | 0.311611111 | 0.0463 | 1.906614011 |
| WARS | 29272 | AP | 1.97E-06 | 4.08E-05 | 0.144394156 | 0.02856 | 1.620539616 |
| PAK6 | 29956 | AP | 2.55E-11 | 1.19E-09 | 0.694488889 | 0.07514 | 2.223823123 |
| ZNF106 | 30163 | AP | 1.10E-11 | 5.36E-10 | 0.346274783 | 0.06302 | 1.703780493 |
| ARPP19 | 30671 | AP | 1.34E-30 | 4.20E-28 | 0.424589655 | 0.02328 | 2.903528562 |
| SMAD3 | 31303 | AP | 0.000687626 | 0.005883385 | 0.068528667 | 0.01256 | 1.696734988 |
| KIF23 | 31390 | AP | 6.34E-05 | 0.000865473 | 0.98337913 | 0.05145 | 2.950384271 |
| FBXL16 | 32957 | AP | 7.98E-12 | 4.06E-10 | 0.109490625 | 0.01266 | 2.157391512 |
| BAIAP3 | 33065 | AP | 1.18E-08 | 3.83E-07 | 0.126246018 | 0.02738 | 1.528419705 |
| ABAT | 33901 | AP | 1.23E-12 | 7.02E-11 | 0.320190968 | 0.04388 | 1.987458957 |
| EMP2 | 33987 | AP | 6.55E-05 | 0.000888849 | 0.251255333 | 0.04774 | 1.660700065 |
| ZC3H7A | 34034 | AP | 3.50E-05 | 0.000520005 | 0.053888889 | 0.00848 | 1.849213864 |
| FAM57B | 36028 | AP | 0.000196997 | 0.002194434 | 0.010671333 | 4.00E-04 | 3.28385175 |
| FAM57B | 36029 | AP | 0.007962992 | 0.035385379 | 0.014369333 | 0.00224 | 1.85862044 |
| LRRC29 | 36981 | AP | 1.01E-09 | 3.90E-08 | 0.409064238 | 0.07088 | 1.7528839 |
| CHTF8 | 37257 | AP | 3.27E-07 | 8.06E-06 | 0.199247742 | 0.03344 | 1.784796197 |
| TUBB3 | 38165 | AP | 5.80E-16 | 5.22E-14 | 0.028125161 | 0.00402 | 1.945382693 |
| DLG4 | 38844 | AP | 5.99E-17 | 6.17E-15 | 0.099210323 | 0.0112 | 2.181328289 |
| ACAP1 | 38921 | AP | 0.000128649 | 0.001547539 | 0.208959286 | 0.03886 | 1.682173984 |
| VAMP2 | 39102 | AP | 8.44E-12 | 4.26E-10 | 0.004374194 | 8.00E-05 | 4.001450815 |
| TIAF1 | 40041 | AP | 1.02E-11 | 5.08E-10 | 0.122180645 | 0.02164 | 1.730957194 |
| RAPGEFL1 | 40847 | AP | 2.06E-08 | 6.43E-07 | 0.405151613 | 0.07322 | 1.710792742 |
| MPP2 | 41734 | AP | 3.21E-20 | 5.03E-18 | 0.264405755 | 0.01764 | 2.707315825 |
| FMNL1 | 41952 | AP | 2.49E-41 | 2.05E-38 | 0.645211382 | 0.06744 | 2.258339674 |
| NSF | 42032 | AP | 0.002473953 | 0.015424781 | 0.004051748 | 0.00032 | 2.538582738 |
| KAT7 | 42321 | AP | 0.001779953 | 0.012289862 | 0.007153947 | 0.00112 | 1.854335599 |
| MSI2 | 42614 | AP | 0.001265589 | 0.009538813 | 0.078369677 | 0.0103 | 2.02929319 |
| SEPT9 | 43716 | AP | 2.83E-05 | 0.000440024 | 0.023155484 | 0.00488 | 1.557086417 |
| KCTD1 | 44991 | AP | 2.52E-06 | 5.14E-05 | 0.018158065 | 0.0016 | 2.429111159 |
| ATP5A1 | 45365 | AP | 4.81E-09 | 1.66E-07 | 0.106013953 | 0.0112 | 2.247656944 |
| CNDP2 | 45810 | AP | 0.000934387 | 0.007576608 | 0.023976774 | 0.005 | 1.567647707 |
| BSG | 46302 | AP | 8.72E-10 | 3.41E-08 | 0.007783226 | 0.00116 | 1.903550875 |
| PDE4A | 47521 | AP | 2.90E-34 | 1.13E-31 | 0.533722951 | 0.04072 | 2.573157513 |
| TPM4 | 48124 | AP | 9.06E-06 | 0.000157662 | 0.140935484 | 0.0305 | 1.530575541 |
| TMEM91 | 50041 | AP | 5.15E-18 | 6.21E-16 | 0.109616774 | 0.01908 | 1.748349746 |
| EML2 | 50493 | AP | 1.63E-06 | 3.46E-05 | 0.730970345 | 0.11256 | 1.870886478 |
| DBP | 50795 | AP | 4.73E-14 | 3.54E-12 | 0.092019355 | 0.00806 | 2.435085377 |
| MYADM | 51740 | AP | 3.09E-06 | 6.23E-05 | 0.02068366 | 0.00362 | 1.742869997 |
| BRSK1 | 52058 | AP | 1.97E-13 | 1.29E-11 | 0.063888732 | 0.01134 | 1.728806716 |
| ZNF211 | 52308 | AP | 1.89E-07 | 4.91E-06 | 0.635780159 | 0.13724 | 1.533121623 |
| VSNL1 | 52728 | AP | 6.78E-07 | 1.64E-05 | 0.024778626 | 0.00208 | 2.477613531 |
| DTNB | 52861 | AP | 3.91E-13 | 2.42E-11 | 0.042914379 | 0.00142 | 3.408550075 |
| CGREF1 | 52932 | AP | 1.10E-06 | 2.41E-05 | 0.044625714 | 0.00784 | 1.739071412 |
| ACYP2 | 53564 | AP | 6.60E-08 | 1.91E-06 | 0.011334194 | 0.00118 | 2.262309696 |
| SPTBN1 | 53577 | AP | 1.67E-05 | 0.00027707 | 0.15529037 | 0.01848 | 2.128607655 |
| MEIS1 | 53806 | AP | 3.76E-05 | 0.000552751 | 0.010079355 | 0.00108 | 2.233528215 |
| KCNIP3 | 54502 | AP | 1.50E-11 | 7.17E-10 | 0.137027206 | 0.00806 | 2.833265933 |
| UXS1 | 54850 | AP | 2.94E-05 | 0.000447277 | 0.016432258 | 0.00246 | 1.899085008 |
| ACTR3 | 55073 | AP | 0.002808756 | 0.016898602 | 0.004222581 | 0.00068 | 1.826108949 |
| AMMECR1L | 55250 | AP | 0.000389754 | 0.003834377 | 0.023835484 | 0.00474 | 1.615138254 |
| GAD1 | 55905 | AP | 3.07E-15 | 2.55E-13 | 0.18149403 | 0.03854 | 1.549526097 |
| RAPGEF4 | 55988 | AP | 4.81E-14 | 3.54E-12 | 0.258555556 | 0.037 | 1.944192673 |
| RAPGEF4 | 55991 | AP | 1.10E-41 | 1.23E-38 | 0.431128148 | 0.02776 | 2.742809238 |
| CHN1 | 56046 | AP | 1.87E-12 | 1.06E-10 | 0.025950323 | 8.00E-04 | 3.479327592 |
| PDE1A | 56450 | AP | 1.33E-51 | 5.20E-48 | 0.732304688 | 0.02788 | 3.268287081 |
| HIBCH | 56559 | AP | 0.001472248 | 0.010746058 | 0.029575484 | 0.00658 | 1.502911025 |
| GLS | 56588 | AP | 8.55E-15 | 6.90E-13 | 0.170982468 | 0.03366 | 1.625250833 |
| SPEG | 57691 | AP | 2.97E-10 | 1.25E-08 | 0.386695205 | 0.07894 | 1.58894873 |
| SDCBP2 | 58483 | AP | 0.000410862 | 0.003967271 | 0.098026087 | 0.01756 | 1.719610048 |
| RBM39 | 59230 | AP | 1.60E-05 | 0.000268471 | 0.007792903 | 0.00118 | 1.887699038 |
| MANBAL | 59337 | AP | 3.26E-12 | 1.77E-10 | 0.048044516 | 0.0066 | 1.985058351 |
| ZFP64 | 59807 | AP | 0.000225254 | 0.002470541 | 0.057824026 | 0.01072 | 1.685293208 |
| APP | 60280 | AP | 4.71E-10 | 1.92E-08 | 0.001528387 | 0.00012 | 2.54447653 |
| RCAN1 | 60494 | AP | 0.000594124 | 0.005317244 | 0.798374194 | 0.14786 | 1.686311522 |
| MPST | 62071 | AP | 2.27E-13 | 1.46E-11 | 0.391688148 | 0.05088 | 2.040996064 |
| MKL1 | 62349 | AP | 4.85E-06 | 9.25E-05 | 0.120867105 | 0.02574 | 1.546645436 |
| PACSIN2 | 62556 | AP | 1.08E-12 | 6.26E-11 | 0.037899355 | 0.0032 | 2.471783279 |
| KIAA0930 | 62648 | AP | 0.000118786 | 0.001448933 | 0.007393548 | 0.00122 | 1.801756922 |
| CLASP2 | 63872 | AP | 1.31E-22 | 2.63E-20 | 0.145050667 | 0.01592 | 2.209506926 |
| TRAK1 | 64266 | AP | 6.51E-06 | 0.000118304 | 0.047219231 | 0.0077 | 1.813580912 |
| ZNF197 | 64374 | AP | 7.81E-05 | 0.001032748 | 0.091223529 | 0.01058 | 2.154347435 |
| PFKFB4 | 64704 | AP | 0.00050147 | 0.004650291 | 0.069884354 | 0.00944 | 2.001885806 |
| ARHGEF3 | 65361 | AP | 2.06E-16 | 1.92E-14 | 0.397998026 | 0.02136 | 2.924927032 |
| PDZRN3 | 65630 | AP | 4.68E-23 | 9.63E-21 | 0.829173469 | 0.11042 | 2.016138108 |
| TMCC1 | 66748 | AP | 9.64E-05 | 0.001229655 | 0.0215 | 0.00254 | 2.135888854 |
| TFDP2 | 67089 | AP | 0.000141657 | 0.001668146 | 0.733958904 | 0.13618 | 1.684475498 |
| KCNAB1 | 67357 | AP | 1.91E-13 | 1.28E-11 | 0.228512295 | 0.02122 | 2.376645883 |
| TRMT44 | 68769 | AP | 0.009065655 | 0.038478669 | 0.046999346 | 0.00922 | 1.628758658 |
| KCNIP4 | 68933 | AP | 2.09E-39 | 1.26E-36 | 0.556641406 | 0.0078 | 4.267797504 |
| LIMCH1 | 69110 | AP | 0.007763091 | 0.034679273 | 0.006335948 | 0.00102 | 1.826436775 |
| LNX1 | 69317 | AP | 0.003852354 | 0.020877358 | 0.011089855 | 0.00178 | 1.829417369 |
| ANK2 | 70393 | AP | 2.70E-38 | 1.51E-35 | 0.387884892 | 0.03674 | 2.356842546 |
| FBXW7 | 70846 | AP | 3.16E-13 | 2.00E-11 | 0.369292105 | 0.05544 | 1.896286589 |
| FBXW7 | 70848 | AP | 6.33E-12 | 3.31E-10 | 0.034451974 | 0.00082 | 3.738017226 |
| MARCH1 | 71023 | AP | 5.14E-06 | 9.71E-05 | 0.78056124 | 0.1284 | 1.804862809 |
| GPM6A | 71209 | AP | 3.37E-19 | 4.80E-17 | 0.048266452 | 0.00158 | 3.419311889 |
| PCSK1 | 72835 | AP | 6.77E-18 | 7.92E-16 | 0.113696183 | 0.0022 | 3.945072471 |
| SYNPO | 74101 | AP | 4.24E-18 | 5.18E-16 | 0.176931933 | 0.00614 | 3.360940356 |
| CYFIP2 | 74323 | AP | 0.002170036 | 0.014040335 | 0.014197761 | 0.00312 | 1.515251288 |
| MGAT1 | 75018 | AP | 3.15E-06 | 6.32E-05 | 0.0977 | 0.02066 | 1.553702095 |
| DAAM2 | 76052 | AP | 4.93E-12 | 2.61E-10 | 0.154294958 | 0.0309 | 1.608109898 |
| GTPBP2 | 76311 | AP | 0.002137805 | 0.013988098 | 0.044074675 | 0.00704 | 1.834277192 |
| ANKRD6 | 77001 | AP | 3.19E-05 | 0.000480117 | 0.481094776 | 0.10154 | 1.555611481 |
| FYN | 77272 | AP | 0.003698951 | 0.020284654 | 0.008872903 | 0.00152 | 1.764291716 |
| NCOA7 | 77428 | AP | 1.60E-26 | 3.91E-24 | 0.582081081 | 0.04206 | 2.627512582 |
| MAP7 | 77914 | AP | 5.72E-12 | 3.01E-10 | 0.426246087 | 0.0931 | 1.521342664 |
| CNKSR3 | 78220 | AP | 4.75E-14 | 3.54E-12 | 0.095986486 | 0.0033 | 3.370284947 |
| SERAC1 | 78252 | AP | 0.007641199 | 0.034271607 | 0.01045461 | 0.00188 | 1.715771246 |
| PRKAR1B | 78505 | AP | 3.80E-10 | 1.57E-08 | 0.046148667 | 0.00754 | 1.811645887 |
| NUDT1 | 78607 | AP | 0.012139409 | 0.047603262 | 0.011464516 | 0.00236 | 1.580595092 |
| WIPI2 | 78654 | AP | 0.006661181 | 0.031273207 | 0.007354839 | 0.00138 | 1.673274925 |
| HDAC9 | 78886 | AP | 7.94E-11 | 3.59E-09 | 0.193262097 | 0.0246 | 2.061300839 |
| SNX10 | 79044 | AP | 2.03E-05 | 0.000330415 | 0.083187943 | 0.01708 | 1.583194236 |
| CHN2 | 79084 | AP | 8.34E-06 | 0.000147475 | 0.644848322 | 0.1207 | 1.675707002 |
| ELMO1 | 79264 | AP | 2.79E-10 | 1.19E-08 | 0.049527097 | 0.0092 | 1.683316445 |
| GRB10 | 79710 | AP | 6.52E-17 | 6.61E-15 | 0.141368702 | 0.00774 | 2.9049697 |
| GRB10 | 79711 | AP | 4.12E-06 | 8.00E-05 | 0.322636641 | 0.06686 | 1.573925859 |
| CPSF4 | 80630 | AP | 7.87E-09 | 2.61E-07 | 0.328229032 | 0.04928 | 1.896193315 |
| PILRB | 80928 | AP | 1.91E-21 | 3.33E-19 | 0.243194194 | 0.02716 | 2.192114972 |
| ST7 | 81555 | AP | 5.79E-06 | 0.000106846 | 0.044309032 | 0.00364 | 2.499204863 |
| PRKAG2 | 82384 | AP | 6.78E-37 | 3.54E-34 | 0.342744371 | 0.06644 | 1.64068561 |
| PRKAG2 | 82387 | AP | 4.52E-07 | 1.10E-05 | 0.035678808 | 0.00718 | 1.603257515 |
| MTUS1 | 82814 | AP | 0.00114703 | 0.008885656 | 0.011087097 | 0.00158 | 1.948357132 |
| GFRA2 | 82905 | AP | 7.02E-08 | 1.98E-06 | 0.081404274 | 0.00224 | 3.592951906 |
| DMTN | 82924 | AP | 6.64E-19 | 9.12E-17 | 0.64694375 | 0.14144 | 1.520383752 |
| ZNF395 | 83211 | AP | 0.000177305 | 0.00201813 | 0.329599048 | 0.05714 | 1.752372512 |
| TPD52 | 84268 | AP | 4.86E-08 | 1.42E-06 | 0.011343791 | 0.00056 | 3.008489028 |
| NECAB1 | 84419 | AP | 6.08E-14 | 4.33E-12 | 0.214489423 | 0.03106 | 1.93233961 |
| ENPP2 | 85002 | AP | 1.17E-07 | 3.16E-06 | 0.457606061 | 0.10182 | 1.502802136 |
| LYNX1 | 85363 | AP | 4.27E-12 | 2.28E-10 | 0.3485625 | 0.07732 | 1.5058649 |
| ARHGEF39 | 86269 | AP | 4.54E-09 | 1.59E-07 | 0.600417105 | 0.05168 | 2.45255373 |
| FRMD3 | 86680 | AP | 8.35E-07 | 1.88E-05 | 0.368902586 | 0.038325 | 2.26443019 |
| RGS3 | 87288 | AP | 0.01065301 | 0.043282207 | 0.012856129 | 0.0026 | 1.59830922 |
| RGS3 | 87289 | AP | 2.65E-08 | 8.07E-07 | 0.021685161 | 0.00158 | 2.619203368 |
| MVB12B | 87593 | AP | 7.68E-05 | 0.001019519 | 0.0372 | 0.00402 | 2.225026859 |
| AK1 | 87673 | AP | 1.05E-39 | 6.86E-37 | 0.192672903 | 0.02392 | 2.08627902 |
| PPP2R4 | 87835 | AP | 1.01E-10 | 4.51E-09 | 0.085897419 | 0.01632 | 1.660762437 |
| AIF1L | 87913 | AP | 0.00035392 | 0.003539652 | 0.017612418 | 0.00312 | 1.730771237 |
| AIF1L | 87914 | AP | 1.86E-13 | 1.26E-11 | 0.043981046 | 0.00564 | 2.053874697 |
| ARHGAP6 | 88471 | AP | 0.004082532 | 0.021792987 | 0.274641667 | 0.06092 | 1.50590569 |
| SH3KBP1 | 88640 | AP | 0.004178794 | 0.022150039 | 0.012233987 | 0.00194 | 1.841529919 |
| SH3KBP1 | 88642 | AP | 4.09E-08 | 1.21E-06 | 0.131547712 | 0.0135 | 2.276679932 |
| CASK | 88857 | AP | 2.31E-07 | 5.83E-06 | 0.048698065 | 0.0039 | 2.524662733 |
| IQSEC2 | 89210 | AP | 6.65E-08 | 1.91E-06 | 0.663720779 | 0.07136 | 2.23012406 |
| DLG3 | 89379 | AP | 7.93E-26 | 1.88E-23 | 0.481322464 | 0.03538 | 2.61039076 |
| DLG3 | 89380 | AP | 2.70E-10 | 1.15E-08 | 0.158515942 | 0.01554 | 2.322437824 |
| TSC22D3 | 89835 | AP | 0.000101368 | 0.001280342 | 0.020638065 | 0.00402 | 1.63585526 |
| GRIA3 | 90009 | AP | 2.45E-05 | 0.000383946 | 0.049282301 | 0.00758 | 1.872051809 |
| DNASE1L1 | 90575 | AP | 6.59E-17 | 6.61E-15 | 0.204417532 | 0.0306 | 1.899164621 |
| SDF4 | 39 | AT | 0.016655191 | 0.042599491 | 0.004323871 | 0.05558 | -2.553672366 |
| ACOT7 | 392 | AT | 0.006793564 | 0.021767076 | 0.002614194 | 0.01236 | -1.553509797 |
| CAMTA1 | 501 | AT | 0.001471545 | 0.006661268 | 0.074064516 | 0.60838 | -2.105863134 |
| UBR4 | 879 | AT | 0.00012254 | 0.001003992 | 0.006345161 | 0.03956 | -1.830125986 |
| CDC42 | 1003 | AT | 0.006878367 | 0.021965754 | 0.020826452 | 0.36206 | -2.855586053 |
| S100PBP | 1636 | AT | 1.36E-05 | 0.000184045 | 0.018280645 | 0.4798 | -3.267526492 |
| ZSCAN20 | 1678 | AT | 0.000136491 | 0.001091505 | 0.058713548 | 0.47348 | -2.087439166 |
| SMIM12 | 1699 | AT | 0.00067837 | 0.00378627 | 0.005185806 | 0.05464 | -2.35484085 |
| INPP5B | 1848 | AT | 8.79E-05 | 0.000770741 | 0.005880645 | 0.13246 | -3.114614236 |
| TTC39A | 2950 | AT | 0.019782526 | 0.048895083 | 0.024410968 | 0.31268 | -2.550147774 |
| SLC35D1 | 3346 | AT | 0.000218594 | 0.001607989 | 0.047690968 | 0.24912 | -1.653192684 |
| HS2ST1 | 3693 | AT | 0.000919879 | 0.004686942 | 0.010819355 | 0.08298 | -2.03726297 |
| PKN2 | 3698 | AT | 4.25E-05 | 0.000443088 | 0.010473548 | 0.18096 | -2.849423135 |
| ABCD3 | 3810 | AT | 0.000654255 | 0.003676245 | 0.016096774 | 0.10844 | -1.907578133 |
| FAM102B | 3940 | AT | 0.005026183 | 0.01711433 | 0.005907097 | 0.08318 | -2.644852463 |
| CYB561D1 | 4015 | AT | 0.005764337 | 0.019112807 | 0.011407097 | 0.0662 | -1.758444777 |
| SLC16A1 | 4265 | AT | 0.001654255 | 0.007282222 | 0.009209032 | 0.20098 | -3.083020631 |
| ENSA | 7495 | AT | 0.000853206 | 0.004420273 | 0.013454839 | 0.08786 | -1.876405842 |
| ILF2 | 7753 | AT | 0.002116749 | 0.008732998 | 0.000929677 | 0.00614 | -1.887742356 |
| TPM3 | 7790 | AT | 0.000677944 | 0.00378627 | 0.041558065 | 0.32096 | -2.04422491 |
| ATP8B2 | 7833 | AT | 0.001812696 | 0.007802386 | 0.009725161 | 0.07616 | -2.058119916 |
| FLAD1 | 7864 | AT | 5.18E-05 | 0.000515944 | 0.017378065 | 0.17984 | -2.336858816 |
| FCER1G | 8600 | AT | 0.00358136 | 0.013016835 | 0.001672903 | 0.06896 | -3.718966051 |
| MGST3 | 8799 | AT | 0.000422025 | 0.00265542 | 0.000822581 | 0.00434 | -1.6631831 |
| TIPRL | 8902 | AT | 0.006993893 | 0.022234072 | 0.006672903 | 0.15238 | -3.12832237 |
| LAMC2 | 9169 | AT | 0.004976189 | 0.017031456 | 0.036001389 | 0.52828 | -2.686068928 |
| PTPRC | 9321 | AT | 0.002176894 | 0.008912201 | 0.007695484 | 0.12666 | -2.800872685 |
| DDX59 | 9334 | AT | 2.02E-05 | 0.000245331 | 0.029789677 | 0.24546 | -2.108972063 |
| NEK2 | 9718 | AT | 0.012294638 | 0.033965392 | 0.062834839 | 0.5445 | -2.159358266 |
| DISC1 | 10282 | AT | 0.00031106 | 0.002081566 | 0.005837419 | 0.18686 | -3.466070865 |
| B3GALNT2 | 10353 | AT | 0.002350057 | 0.009406448 | 0.046723226 | 0.26784 | -1.746148406 |
| HEATR1 | 10395 | AT | 0.001281732 | 0.006013919 | 0.002742581 | 0.08232 | -3.401714773 |
| ZNF124 | 10514 | AT | 4.01E-05 | 0.000426912 | 0.0621 | 0.3679 | -1.779065173 |
| PFKFB3 | 10695 | AT | 0.006862098 | 0.021941067 | 0.006001935 | 0.02838 | -1.553602674 |
| SEC61A2 | 10758 | AT | 0.020014991 | 0.049327226 | 0.001995484 | 0.00968 | -1.579175339 |
| RPP38 | 10861 | AT | 0.000544773 | 0.003223361 | 0.026385161 | 0.29344 | -2.408871409 |
| NRP1 | 11195 | AT | 0.003360834 | 0.012420263 | 0.003695484 | 0.03044 | -2.108646034 |
| ZFAND4 | 11368 | AT | 0.011067246 | 0.031454195 | 0.015865584 | 0.10488 | -1.888664578 |
| VSTM4 | 11499 | AT | 0.000626854 | 0.003573892 | 0.021118065 | 0.11698 | -1.711874168 |
| RTKN2 | 11871 | AT | 0.011990499 | 0.033394059 | 0.055868387 | 0.3419 | -1.811519602 |
| KCNMA1 | 12280 | AT | 0.00550504 | 0.018347752 | 0.054549032 | 0.45426 | -2.11956975 |
| RPS24 | 12294 | AT | 4.06E-05 | 0.000429748 | 0.001343871 | 0.00844 | -1.837428077 |
| STAMBPL1 | 12472 | AT | 0.002401334 | 0.009575018 | 0.006777778 | 0.06756 | -2.299366805 |
| KIF20B | 12497 | AT | 0.000119699 | 0.000985858 | 0.144878065 | 0.77438 | -1.676170255 |
| FAM178A | 12841 | AT | 1.32E-05 | 0.000180956 | 0.017954194 | 0.14668 | -2.100429632 |
| VTI1A | 13116 | AT | 0.006635953 | 0.02135082 | 0.01452 | 0.08174 | -1.728016469 |
| VWA2 | 13198 | AT | 0.011276875 | 0.031949075 | 0.042883621 | 0.2519 | -1.770542232 |
| FGFR2 | 13296 | AT | 0.004222745 | 0.01486445 | 0.023597419 | 0.22062 | -2.235304408 |
| CHID1 | 13806 | AT | 1.31E-07 | 3.20E-06 | 0.090777419 | 0.76348 | -2.12947636 |
| MRPL23 | 13893 | AT | 0.020314832 | 0.049874854 | 0.016441935 | 0.11894 | -1.978784051 |
| ZNF195 | 13964 | AT | 0.002776846 | 0.010714067 | 0.003493548 | 0.021 | -1.793604488 |
| ZNF215 | 14188 | AT | 0.000574915 | 0.003347196 | 0.087612931 | 0.60738 | -1.936226023 |
| SAA2 | 14577 | AT | 0.000204394 | 0.001538747 | 0.06891938 | 0.83354 | -2.492744278 |
| TSG101 | 14662 | AT | 0.001515237 | 0.006811057 | 0.010582581 | 0.06486 | -1.813021787 |
| UEVLD | 14666 | AT | 2.78E-07 | 6.19E-06 | 0.014825806 | 0.22328 | -2.712057248 |
| SLC5A12 | 14745 | AT | 0.013172505 | 0.035709679 | 0.074382222 | 0.43336 | -1.762351827 |
| NDUFS3 | 15782 | AT | 3.41E-05 | 0.000368447 | 0.009023226 | 0.0619 | -1.925718281 |
| RBM4 | 17090 | AT | 0.016429069 | 0.042189104 | 0.000393548 | 0.01076 | -3.308386807 |
| SHANK2 | 17408 | AT | 0.011877703 | 0.033169667 | 0.005778523 | 0.03168 | -1.701537368 |
| ANAPC15 | 17566 | AT | 1.28E-05 | 0.000176305 | 0.016980645 | 0.11186 | -1.885173914 |
| NEU3 | 17812 | AT | 0.000677221 | 0.00378627 | 0.046008387 | 0.3301 | -1.970571931 |
| AAMDC | 17974 | AT | 0.000267912 | 0.001857315 | 0.026838065 | 0.19986 | -2.007795924 |
| INTS4 | 17989 | AT | 0.000152756 | 0.00119918 | 0.009233548 | 0.08494 | -2.21910171 |
| TMEM135 | 18206 | AT | 0.000828713 | 0.004319506 | 0.000746452 | 0.05034 | -4.211224472 |
| MED17 | 18327 | AT | 2.00E-05 | 0.000244236 | 0.036562581 | 0.2612 | -1.966261067 |
| ACAT1 | 18599 | AT | 0.0020417 | 0.008484887 | 0.003019355 | 0.01652 | -1.699528589 |
| DDX10 | 18623 | AT | 0.001410779 | 0.00645917 | 0.012761935 | 0.07438 | -1.762720138 |
| SDHD | 18731 | AT | 0.016261396 | 0.0418003 | 0.001198065 | 0.01528 | -2.545837432 |
| SDHD | 18733 | AT | 0.005714967 | 0.018965424 | 0.00120129 | 0.02324 | -2.962478684 |
| SDHD | 18734 | AT | 8.46E-05 | 0.000746507 | 0.002852903 | 0.01374 | -1.571974135 |
| GLB1L2 | 19563 | AT | 0.002210375 | 0.009030091 | 0.058053846 | 0.35174 | -1.801521304 |
| PARP11 | 19761 | AT | 0.007313417 | 0.023068385 | 0.015791613 | 0.07908 | -1.610981028 |
| TNFRSF1A | 19829 | AT | 0.001445343 | 0.006584468 | 0.027032258 | 0.13696 | -1.622658016 |
| PLEKHA5 | 20647 | AT | 0.000747597 | 0.003999681 | 0.020860645 | 0.11178 | -1.678668277 |
| RASSF8 | 20838 | AT | 0.000696227 | 0.003841958 | 0.001007097 | 0.09554 | -4.552473297 |
| ALG10 | 21065 | AT | 0.002164548 | 0.008871079 | 0.018499355 | 0.3204 | -2.851834357 |
| ALG10B | 21068 | AT | 0.001171231 | 0.005597669 | 0.059943226 | 0.3132 | -1.653444086 |
| ANO6 | 21298 | AT | 0.002672015 | 0.010371885 | 0.01469871 | 0.12904 | -2.172362721 |
| TROAP | 21551 | AT | 0.000119368 | 0.000985231 | 0.1555 | 0.91408 | -1.771272363 |
| SPATS2 | 21577 | AT | 0.008463072 | 0.025702334 | 0.020210968 | 0.15908 | -2.063181806 |
| MYL6 | 22375 | AT | 0.000312527 | 0.002083627 | 0.028777419 | 0.16034 | -1.717705531 |
| HMGA2 | 22881 | AT | 0.004808711 | 0.016519764 | 0.004792086 | 0.60902 | -4.844885232 |
| RAP1B | 22935 | AT | 0.000534335 | 0.003173486 | 0.002536774 | 0.08724 | -3.53776967 |
| CNOT2 | 23366 | AT | 0.00253484 | 0.009969939 | 0.019755484 | 0.23808 | -2.489175634 |
| TMEM19 | 23410 | AT | 0.00015845 | 0.001240089 | 0.013569032 | 0.06458 | -1.560114609 |
| DCN | 23655 | AT | 0.009276758 | 0.027516172 | 0.000853548 | 0.07202 | -4.435296904 |
| APPL2 | 24130 | AT | 0.011434915 | 0.032187337 | 0.003311613 | 0.02454 | -2.002869087 |
| TCP11L2 | 24138 | AT | 0.003293402 | 0.012229691 | 0.002968387 | 0.03168 | -2.367666827 |
| HNF1A | 24784 | AT | 0.000574234 | 0.003347196 | 0.109843871 | 0.49822 | -1.511981744 |
| KDM2B | 24877 | AT | 4.15E-05 | 0.000436226 | 0.019980645 | 0.17554 | -2.173102874 |
| ABCB9 | 24999 | AT | 0.000404876 | 0.002564661 | 0.013046452 | 0.1364 | -2.347075556 |
| TPT1 | 25796 | AT | 0.000193124 | 0.001467552 | 0.001274839 | 0.00634 | -1.6040591 |
| LRCH1 | 25842 | AT | 0.001261531 | 0.005959035 | 0.028373548 | 0.20468 | -1.975990468 |
| ARHGEF7 | 26283 | AT | 0.002258982 | 0.009165521 | 0.064125161 | 0.62616 | -2.278769111 |
| MCF2L | 26328 | AT | 0.001415688 | 0.006473036 | 0.006876129 | 0.07532 | -2.393689851 |
| SEC23A | 27348 | AT | 0.000121687 | 0.000999025 | 0.059602581 | 0.2891 | -1.579073776 |
| TRIM9 | 27503 | AT | 0.005091549 | 0.017262717 | 0.076250323 | 0.3425 | -1.502250012 |
| LGALS3 | 27618 | AT | 0.000314061 | 0.002088977 | 0.002941935 | 0.1875 | -4.154711153 |
| PCNXL4 | 27765 | AT | 0.00582538 | 0.019262356 | 0.00109871 | 0.00716 | -1.87437351 |
| ERH | 28140 | AT | 0.010591062 | 0.030453747 | 0.000900645 | 0.00422 | -1.544479055 |
| TTLL5 | 28520 | AT | 0.000258366 | 0.001806861 | 0.00928129 | 0.09566 | -2.332799658 |
| WDR20 | 29339 | AT | 0.000963264 | 0.004878977 | 0.002485806 | 0.02736 | -2.398484958 |
| EMC4 | 29839 | AT | 0.000145982 | 0.001157795 | 0.002048387 | 0.03026 | -2.692774007 |
| FAM98B | 29920 | AT | 3.29E-05 | 0.000357759 | 0.011681935 | 0.06418 | -1.703647963 |
| C15orf57 | 29992 | AT | 9.18E-05 | 0.000797487 | 0.026054839 | 0.15176 | -1.762096828 |
| DYX1C1 | 30732 | AT | 0.000658349 | 0.003696438 | 0.034125161 | 0.19704 | -1.753371774 |
| TPM1 | 30984 | AT | 2.77E-05 | 0.000311838 | 0.045954194 | 0.37892 | -2.109679993 |
| CD276 | 31610 | AT | 7.17E-05 | 0.000663096 | 0.019012903 | 0.13938 | -1.99208615 |
| HYKK | 32099 | AT | 0.000582038 | 0.003373028 | 0.055347458 | 0.28664 | -1.644596348 |
| AKAP13 | 32350 | AT | 0.006226636 | 0.020211004 | 0.007591613 | 0.08706 | -2.439553464 |
| ACAN | 32396 | AT | 0.004363414 | 0.015296892 | 0.032956552 | 0.29808 | -2.202171827 |
| PKMYT1 | 33328 | AT | 1.87E-05 | 0.000231333 | 0.115092258 | 0.67718 | -1.772203066 |
| TMC7 | 34279 | AT | 0.006208241 | 0.020197053 | 0.046643226 | 0.23748 | -1.627555706 |
| LYRM1 | 34406 | AT | 0.000252308 | 0.001778147 | 0.004231613 | 0.03832 | -2.203388732 |
| INO80E | 36003 | AT | 0.001746301 | 0.007563053 | 0.011645161 | 0.06136 | -1.661867403 |
| CKLF | 36727 | AT | 0.008021679 | 0.024711247 | 0.024725806 | 0.1111 | -1.502583203 |
| CMTM3 | 36808 | AT | 0.008302256 | 0.025374856 | 0.004718065 | 0.02336 | -1.599626503 |
| PDP2 | 36881 | AT | 0.001438694 | 0.006562656 | 0.002568387 | 0.03322 | -2.559873991 |
| LRRC36 | 37015 | AT | 0.002054875 | 0.008530443 | 0.091769032 | 0.47768 | -1.649666151 |
| NFATC3 | 37178 | AT | 0.002516443 | 0.009907693 | 0.00706129 | 0.17408 | -3.204887164 |
| NFAT5 | 37290 | AT | 0.003487587 | 0.012736055 | 0.003572903 | 0.01926 | -1.684651913 |
| DDX19B | 37343 | AT | 0.002887697 | 0.011042779 | 0.006592258 | 0.14908 | -3.118587135 |
| BCMO1 | 37763 | AT | 0.005100833 | 0.017276944 | 0.062968874 | 0.49602 | -2.063975705 |
| OSGIN1 | 37799 | AT | 0.001411818 | 0.00645917 | 0.009814839 | 0.09778 | -2.298824663 |
| C16orf74 | 37886 | AT | 0.001670677 | 0.007341672 | 0.043112418 | 0.24146 | -1.722892745 |
| KLHDC4 | 37949 | AT | 0.000482759 | 0.002932697 | 0.002107097 | 0.10494 | -3.908077695 |
| TRPV3 | 38443 | AT | 0.00608276 | 0.019911676 | 0.037534014 | 0.224 | -1.786398501 |
| SHPK | 38463 | AT | 3.25E-05 | 0.000355652 | 0.027198065 | 0.19538 | -1.971800567 |
| MED11 | 38578 | AT | 9.31E-05 | 0.000805707 | 0.049169032 | 0.3027 | -1.817478216 |
| MPDU1 | 38997 | AT | 0.000116211 | 0.000962498 | 0.000730323 | 0.08668 | -4.776492128 |
| TP53 | 39036 | AT | 6.01E-05 | 0.00057735 | 0.006822581 | 0.13364 | -2.974911823 |
| NDEL1 | 39187 | AT | 1.72E-05 | 0.00021882 | 0.004317419 | 0.0329 | -2.030814806 |
| ZNF286A | 39371 | AT | 0.008458694 | 0.025702334 | 0.025480645 | 0.17798 | -1.943752034 |
| CCDC144A | 39441 | AT | 0.000343602 | 0.002256243 | 0.005045161 | 0.02354 | -1.540271478 |
| GID4 | 39538 | AT | 0.001070828 | 0.005259176 | 0.022451613 | 0.10246 | -1.518110023 |
| TMEM98 | 40215 | AT | 0.005016149 | 0.01711433 | 0.022704516 | 0.12668 | -1.719100369 |
| MLLT6 | 40581 | AT | 1.82E-05 | 0.000228013 | 0.001071613 | 0.01308 | -2.501919446 |
| KRT222 | 40883 | AT | 0.001521551 | 0.006827494 | 0.025992258 | 0.29132 | -2.416623592 |
| JUP | 40931 | AT | 0.011382655 | 0.032107373 | 0.079320645 | 0.43798 | -1.70867481 |
| TMUB2 | 41787 | AT | 1.91E-08 | 5.54E-07 | 0.04603871 | 0.30304 | -1.884382253 |
| XYLT2 | 42366 | AT | 0.001131329 | 0.005481721 | 0.011514194 | 0.11218 | -2.276524228 |
| MBTD1 | 42518 | AT | 3.22E-05 | 0.00035337 | 0.020506452 | 0.28398 | -2.628164264 |
| TOM1L1 | 42535 | AT | 0.002600763 | 0.010156676 | 0.026574675 | 0.16734 | -1.840068964 |
| RNF43 | 42671 | AT | 0.004757789 | 0.016403307 | 0.022552288 | 0.26648 | -2.469462688 |
| MTMR4 | 42682 | AT | 0.013059605 | 0.035511991 | 0.002950323 | 0.02076 | -1.951113545 |
| ABCA9 | 43158 | AT | 0.002802261 | 0.010790526 | 0.016964516 | 0.10486 | -1.821502251 |
| MRPS7 | 43411 | AT | 6.34E-05 | 0.000599714 | 0.005051613 | 0.03314 | -1.881033433 |
| ANAPC11 | 44203 | AT | 0.004667512 | 0.016142599 | 0.004892903 | 0.0222 | -1.512306455 |
| TWSG1 | 44599 | AT | 0.000288166 | 0.001962429 | 0.016916774 | 0.22936 | -2.606987136 |
| IMPA2 | 44660 | AT | 0.000402914 | 0.002558539 | 0.007930968 | 0.29734 | -3.624101201 |
| ELAC1 | 45554 | AT | 0.000280698 | 0.001926895 | 0.028735484 | 0.26584 | -2.224761893 |
| SLC39A3 | 46621 | AT | 0.002718688 | 0.01051599 | 0.004990323 | 0.06504 | -2.567501912 |
| ZNF559 | 47273 | AT | 2.51E-12 | 1.29E-10 | 0.052816774 | 0.27086 | -1.634773249 |
| ZNF846 | 47400 | AT | 0.000121451 | 0.000999025 | 0.014969281 | 0.18872 | -2.534264264 |
| ZNF20 | 47777 | AT | 6.90E-06 | 0.000104428 | 0.014605806 | 0.44876 | -3.425069072 |
| EMR2 | 48028 | AT | 0.003109575 | 0.011642406 | 0.084163636 | 0.47384 | -1.728106756 |
| USE1 | 48235 | AT | 6.81E-05 | 0.000635755 | 0.04169871 | 0.193 | -1.532220004 |
| PIK3R2 | 48396 | AT | 0.004414781 | 0.015455864 | 0.059059355 | 0.36824 | -1.830191947 |
| NDUFA13 | 48647 | AT | 0.002168872 | 0.008884076 | 0.003390968 | 0.03778 | -2.410664512 |
| ZNF506 | 48680 | AT | 0.001709637 | 0.007475661 | 0.069455484 | 0.3316 | -1.563243396 |
| ZNF430 | 48742 | AT | 0.000462349 | 0.002843231 | 0.007383226 | 0.22104 | -3.399133036 |
| ZNF738 | 48763 | AT | 0.000932037 | 0.004745756 | 0.047270968 | 0.33286 | -1.951825664 |
| HKR1 | 49481 | AT | 1.44E-07 | 3.51E-06 | 0.09558 | 0.45674 | -1.564150708 |
| YIF1B | 49606 | AT | 3.15E-06 | 5.19E-05 | 0.071922581 | 0.4663 | -1.869238932 |
| TMEM145 | 50152 | AT | 5.98E-06 | 9.16E-05 | 0.033406579 | 0.17618 | -1.662753345 |
| PHLDB3 | 50197 | AT | 0.004244928 | 0.014924627 | 0.034354194 | 0.16956 | -1.596482752 |
| ETHE1 | 50198 | AT | 0.004566385 | 0.015878332 | 0.009985161 | 0.1146 | -2.440347684 |
| ZNF283 | 50245 | AT | 8.33E-06 | 0.000121826 | 0.001646452 | 0.62604 | -5.940791832 |
| PTGIR | 50570 | AT | 0.001223058 | 0.005812919 | 0.065521429 | 0.69422 | -2.36041167 |
| FLT3LG | 50943 | AT | 0.000140879 | 0.001120782 | 0.041690323 | 0.40484 | -2.273222898 |
| VRK3 | 51146 | AT | 0.000505489 | 0.003020784 | 0.06216129 | 0.35588 | -1.744861131 |
| CD33 | 51314 | AT | 0.000130929 | 0.001058006 | 0.035387742 | 0.21136 | -1.787197354 |
| ZNF83 | 51481 | AT | 4.10E-05 | 0.000432505 | 0.128821935 | 0.61828 | -1.568510324 |
| NDUFA3 | 51776 | AT | 3.86E-06 | 6.25E-05 | 0.05076129 | 0.71464 | -2.644644857 |
| RPS9 | 51815 | AT | 0.000242089 | 0.001737275 | 0.000134194 | 0.0114 | -4.442085486 |
| FCAR | 51953 | AT | 0.000969001 | 0.004898382 | 0.057815862 | 0.40902 | -1.956500886 |
| ZNF606 | 52380 | AT | 0.00031272 | 0.002083627 | 0.040846452 | 0.33626 | -2.108064717 |
| IAH1 | 52630 | AT | 0.001517683 | 0.006814104 | 0.010753548 | 0.1475 | -2.618592393 |
| ADAM17 | 52635 | AT | 0.009722948 | 0.028564865 | 0.006225161 | 0.14426 | -3.143017875 |
| GREB1 | 52703 | AT | 0.000546166 | 0.003226356 | 0.003439355 | 0.03812 | -2.405455172 |
| HS1BP3 | 52780 | AT | 0.015569816 | 0.040454234 | 0.004992258 | 0.09478 | -2.943670091 |
| MPV17 | 52964 | AT | 0.000210386 | 0.001563995 | 0.010802581 | 0.05712 | -1.665369264 |
| IFT172 | 53017 | AT | 0.001343471 | 0.006273059 | 0.043544516 | 0.29458 | -1.911766839 |
| LCLAT1 | 53115 | AT | 3.45E-05 | 0.000371231 | 0.04176 | 0.319 | -2.033252159 |
| THADA | 53377 | AT | 0.00032233 | 0.0021282 | 0.000429677 | 0.00358 | -2.12008334 |
| THADA | 53380 | AT | 0.000107277 | 0.000906798 | 0.074068387 | 0.36316 | -1.589854692 |
| THADA | 53381 | AT | 5.55E-05 | 0.000544953 | 0.001366452 | 0.03188 | -3.149761538 |
| SLC3A1 | 53422 | AT | 0.008057952 | 0.02478801 | 0.015080519 | 0.08806 | -1.76461459 |
| TTC7A | 53484 | AT | 0.005060903 | 0.017217345 | 0.002312258 | 0.05128 | -3.099076248 |
| ACYP2 | 53569 | AT | 0.000889685 | 0.004590753 | 0.000918065 | 0.0105 | -2.436862869 |
| COMMD1 | 53710 | AT | 0.016572962 | 0.042459074 | 0.00069871 | 0.0139 | -2.990408803 |
| MEIS1 | 53807 | AT | 0.002619959 | 0.010216119 | 0.032797419 | 0.16304 | -1.603645735 |
| SFXN5 | 53946 | AT | 0.009804986 | 0.028735748 | 0.001271613 | 0.02014 | -2.76242179 |
| MOB1A | 54021 | AT | 0.0002676 | 0.001856822 | 0.010653548 | 0.11124 | -2.345797011 |
| COA5 | 54638 | AT | 4.99E-05 | 0.000502367 | 0.007242581 | 0.11334 | -2.750414565 |
| ST6GAL2 | 54867 | AT | 0.013618218 | 0.036707288 | 0.022957419 | 0.12198 | -1.670215929 |
| PLEKHB2 | 55369 | AT | 0.000748675 | 0.003999681 | 0.000217419 | 0.02834 | -4.870201514 |
| ARHGAP15 | 55490 | AT | 0.005423454 | 0.018146443 | 0.001648052 | 0.07936 | -3.87440051 |
| METTL5 | 55889 | AT | 2.06E-06 | 3.62E-05 | 0.00520129 | 0.05806 | -2.412570225 |
| HAT1 | 55961 | AT | 2.01E-05 | 0.000245331 | 0.009727742 | 0.14216 | -2.681971387 |
| CCDC141 | 56396 | AT | 0.007802464 | 0.024204908 | 0.049184892 | 0.23384 | -1.559050616 |
| SPAG16 | 57328 | AT | 0.00145362 | 0.00660339 | 0.002247742 | 0.01592 | -1.957650052 |
| USP37 | 57485 | AT | 0.008387281 | 0.02556645 | 0.013667742 | 0.09934 | -1.983509857 |
| RHBDD1 | 57792 | AT | 0.010835511 | 0.031020005 | 0.006445806 | 0.0359 | -1.717307539 |
| SP140L | 57887 | AT | 2.38E-05 | 0.0002788 | 0.058485161 | 0.27186 | -1.536514159 |
| DIS3L2 | 57982 | AT | 5.95E-05 | 0.000573145 | 0.027412258 | 0.1791 | -1.876954021 |
| CSNK2A1 | 58460 | AT | 2.79E-05 | 0.000313394 | 0.005350968 | 0.02932 | -1.700992446 |
| POFUT1 | 58941 | AT | 0.004574511 | 0.015892251 | 0.006483871 | 0.05542 | -2.145622836 |
| AAR2 | 59277 | AT | 0.017110737 | 0.043576701 | 0.000734839 | 0.00552 | -2.016482107 |
| ZFP64 | 59810 | AT | 0.001107269 | 0.005405914 | 0.004425161 | 0.02152 | -1.581676008 |
| GNAS | 59998 | AT | 1.35E-05 | 0.000184045 | 0.00419871 | 0.02286 | -1.6946114 |
| LAMA5 | 60060 | AT | 0.0003393 | 0.002231794 | 0.007699355 | 0.27064 | -3.559652986 |
| KCNQ2 | 60121 | AT | 0.000102486 | 0.000875903 | 0.107848026 | 0.86922 | -2.086873185 |
| JAM2 | 60254 | AT | 2.48E-05 | 0.000284086 | 0.022469677 | 0.15562 | -1.935250408 |
| ATP5J | 60260 | AT | 0.000221354 | 0.001625274 | 0.000960645 | 0.0341 | -3.569447562 |
| GRIK1 | 60325 | AT | 0.006517468 | 0.021031975 | 0.000300658 | 0.04856 | -5.08458237 |
| DONSON | 60441 | AT | 0.001068468 | 0.005259176 | 0.016574194 | 0.1958 | -2.46924685 |
| CRYZL1 | 60451 | AT | 1.68E-05 | 0.000214898 | 0.015065806 | 0.15906 | -2.356853787 |
| ITSN1 | 60466 | AT | 0.000821239 | 0.004289246 | 0.091141935 | 0.44996 | -1.596740668 |
| SUMO3 | 60834 | AT | 0.002599067 | 0.010155198 | 0.017012903 | 0.1181 | -1.937559654 |
| COMT | 61100 | AT | 0.006102522 | 0.019942494 | 0.005374194 | 0.03412 | -1.848275197 |
| KREMEN1 | 61569 | AT | 0.001821721 | 0.007828314 | 0.004893548 | 0.10088 | -3.02601401 |
| POLR2F | 62179 | AT | 0.000720412 | 0.003927622 | 0.037465161 | 0.20316 | -1.690582377 |
| POLR2F | 62182 | AT | 0.001866518 | 0.007967325 | 0.026369677 | 0.35002 | -2.585775528 |
| SUMF1 | 62984 | AT | 3.22E-05 | 0.00035337 | 0.0146 | 0.11886 | -2.096924801 |
| FANCD2 | 63304 | AT | 0.000391696 | 0.002507963 | 0.006842581 | 0.28556 | -3.731287217 |
| FANCD2 | 63305 | AT | 0.001057879 | 0.00523104 | 0.039702581 | 0.20232 | -1.628434413 |
| RPL15 | 63730 | AT | 0.000755807 | 0.004026616 | 0.003545806 | 0.03554 | -2.304893199 |
| MLH1 | 63930 | AT | 0.00022332 | 0.001636474 | 0.01413871 | 0.10582 | -2.012823135 |
| MAP4 | 64549 | AT | 0.009248685 | 0.027454386 | 0.022263226 | 0.3726 | -2.817569208 |
| USP4 | 64851 | AT | 7.08E-05 | 0.000656705 | 0.004785806 | 0.09312 | -2.968234436 |
| SLMAP | 65393 | AT | 3.85E-06 | 6.23E-05 | 0.002337419 | 0.04276 | -2.906555605 |
| PPP4R2 | 65624 | AT | 7.48E-05 | 0.000683931 | 0.002595484 | 0.06502 | -3.220921953 |
| PHLDB2 | 66062 | AT | 0.000180374 | 0.00138221 | 0.003585161 | 0.08788 | -3.199168784 |
| RBP1 | 67032 | AT | 0.001013185 | 0.005053855 | 0.015268387 | 0.07266 | -1.560006539 |
| NMD3 | 67508 | AT | 0.00112391 | 0.005456014 | 0.011821935 | 0.09674 | -2.102070222 |
| PHC3 | 67602 | AT | 0.000477146 | 0.002910063 | 0.028516129 | 0.16888 | -1.778718545 |
| VPS8 | 67972 | AT | 0.000772319 | 0.004094778 | 0.005176774 | 0.02862 | -1.709923654 |
| MAP3K13 | 67987 | AT | 0.001178062 | 0.005623329 | 0.032429032 | 0.15472 | -1.562562953 |
| IL1RAP | 68106 | AT | 0.008492395 | 0.025764609 | 0.008944516 | 0.35314 | -3.675823957 |
| TMEM44 | 68156 | AT | 1.62E-05 | 0.000209463 | 0.021909032 | 0.20162 | -2.219485754 |
| C4orf19 | 69000 | AT | 0.00224334 | 0.009135714 | 0.06368961 | 0.40326 | -1.845560068 |
| KLHL5 | 69041 | AT | 0.000466036 | 0.002856761 | 0.01472 | 0.10418 | -1.956913059 |
| SEPT11 | 69617 | AT | 0.000578345 | 0.00335716 | 0.043388387 | 0.34688 | -2.078787072 |
| PLAC8 | 69773 | AT | 0.004427605 | 0.01547955 | 0.011972848 | 0.12112 | -2.314140397 |
| GSTCD | 70253 | AT | 0.002572746 | 0.010066048 | 0.003929032 | 0.03684 | -2.238191061 |
| RPL34 | 70298 | AT | 7.71E-06 | 0.000114227 | 0.027998065 | 0.73342 | -3.265583142 |
| NAA15 | 70629 | AT | 8.34E-05 | 0.000738394 | 0.009318065 | 0.09116 | -2.280661267 |
| MGST2 | 70632 | AT | 0.012271803 | 0.033957734 | 0.038815584 | 0.1785 | -1.525766775 |
| INPP4B | 70687 | AT | 0.00324417 | 0.012087631 | 0.112351613 | 0.69252 | -1.818703764 |
| PALLD | 71124 | AT | 0.000724318 | 0.003932232 | 6.39E-05 | 0.06112 | -6.863729505 |
| NEIL3 | 71226 | AT | 0.000251487 | 0.001778147 | 0.038509868 | 0.72058 | -2.929141912 |
| KIAA1430 | 71331 | AT | 0.004472087 | 0.015599693 | 0.003692258 | 0.02854 | -2.0450684 |
| PDCD6 | 71425 | AT | 0.008523048 | 0.025847434 | 0.001612258 | 0.01402 | -2.162849161 |
| CARD6 | 71874 | AT | 0.013904128 | 0.037282889 | 0.005143226 | 0.17286 | -3.514801547 |
| CENPK | 72213 | AT | 0.003977213 | 0.01414343 | 0.045757419 | 0.5857 | -2.549453763 |
| MAST4 | 72280 | AT | 0.001566799 | 0.006989847 | 0.003920645 | 0.09544 | -3.192241556 |
| FCHO2 | 72481 | AT | 0.000310024 | 0.002076434 | 0.003476129 | 0.10634 | -3.42072218 |
| RIOK2 | 72877 | AT | 5.39E-07 | 1.12E-05 | 0.049921935 | 0.3474 | -1.940016359 |
| NREP | 72977 | AT | 0.001446892 | 0.006584468 | 0.000430323 | 0.00294 | -1.921629745 |
| MARCH3 | 73178 | AT | 0.016083376 | 0.041374782 | 0.013832258 | 0.20068 | -2.674708194 |
| CDKL3 | 73366 | AT | 4.18E-05 | 0.000437757 | 0.097437419 | 0.51688 | -1.66860042 |
| SLC36A2 | 74163 | AT | 0.005321381 | 0.017851404 | 0 | 0.28134 | #NAME? |
| GALNT10 | 74213 | AT | 0.000107629 | 0.00090746 | 0.008057419 | 0.06036 | -2.013733308 |
| ADAM19 | 74384 | AT | 0.00506689 | 0.017218015 | 0.005213548 | 0.03618 | -1.937245784 |
| ERGIC1 | 74560 | AT | 0.000113576 | 0.000948199 | 0.001954194 | 0.03136 | -2.775555594 |
| CREBRF | 74576 | AT | 0.000426463 | 0.002668685 | 0.013576774 | 0.06272 | -1.530319823 |
| RNF130 | 74993 | AT | 0.014448645 | 0.038382014 | 0.050813548 | 0.54694 | -2.376176088 |
| GNB2L1 | 75056 | AT | 0.002417648 | 0.009621913 | 0.002904516 | 0.02024 | -1.941394033 |
| SSR1 | 75255 | AT | 0.000942251 | 0.004785371 | 0.007173548 | 0.05876 | -2.103060926 |
| TMEM14B | 75314 | AT | 0.000121579 | 0.000999025 | 0.000332258 | 0.02252 | -4.216247113 |
| NEDD9 | 75339 | AT | 0.00658166 | 0.021211561 | 0.016363226 | 0.18856 | -2.44437977 |
| PPARD | 75907 | AT | 4.64E-05 | 0.00047471 | 0.022427097 | 0.15264 | -1.917812302 |
| FKBP5 | 75918 | AT | 0.009775758 | 0.028698142 | 0.008816774 | 0.06204 | -1.951123273 |
| MAPK13 | 75951 | AT | 0.000970588 | 0.004899977 | 0.035667742 | 0.3421 | -2.260856399 |
| UBR2 | 76182 | AT | 0.001100066 | 0.005380484 | 0.001419355 | 0.072 | -3.92646369 |
| PTK7 | 76245 | AT | 0.00181933 | 0.007826573 | 0.008176774 | 0.14722 | -2.890630347 |
| POLR1C | 76302 | AT | 0.000776139 | 0.004109405 | 0.070881935 | 0.39698 | -1.722870289 |
| LRRC1 | 76510 | AT | 0.008894871 | 0.026702904 | 0.006620645 | 0.08188 | -2.515061939 |
| MLIP | 76516 | AT | 0.000427566 | 0.002673239 | 0.006171324 | 0.06372 | -2.33458516 |
| UBE3D | 76842 | AT | 7.55E-05 | 0.000686914 | 0.011993548 | 0.11274 | -2.240715412 |
| RNGTT | 76982 | AT | 4.60E-05 | 0.0004738 | 0.003568387 | 0.16466 | -3.831769042 |
| CCNC | 77076 | AT | 0.000105516 | 0.000897217 | 0.007035484 | 0.04434 | -1.840920735 |
| GRIK2 | 77097 | AT | 0.009235797 | 0.027454386 | 0.049211613 | 0.28702 | -1.763422269 |
| SGK1 | 77774 | AT | 0.002252868 | 0.009155711 | 0.00040129 | 0.0041 | -2.324057091 |
| AIG1 | 77971 | AT | 2.75E-05 | 0.000310351 | 0.013205806 | 0.14594 | -2.402538963 |
| NUP43 | 78095 | AT | 4.49E-06 | 7.11E-05 | 0.009986452 | 0.35708 | -3.576730511 |
| IYD | 78139 | AT | 0.008729838 | 0.026370817 | 0.0117968 | 0.1639 | -2.631428177 |
| SYNE1 | 78179 | AT | 0.015608141 | 0.040509308 | 0.000696774 | 0.00546 | -2.05874268 |
| HEATR2 | 78512 | AT | 0.012544607 | 0.034476426 | 0.002639355 | 0.02286 | -2.15885415 |
| TNRC18 | 78663 | AT | 0.001362849 | 0.00631762 | 0.003541935 | 0.04968 | -2.640929113 |
| C7orf10 | 79306 | AT | 0.002328153 | 0.009333195 | 0.008662581 | 0.1884 | -3.079554688 |
| PSMA2 | 79317 | AT | 4.51E-07 | 9.59E-06 | 0.014684516 | 0.06994 | -1.560844118 |
| OGDH | 79543 | AT | 1.44E-05 | 0.000193044 | 0.03281871 | 0.14724 | -1.501065139 |
| H2AFV | 79571 | AT | 0.000397574 | 0.002541373 | 0.00399871 | 0.04162 | -2.342609093 |
| UPP1 | 79633 | AT | 0.010474781 | 0.030153132 | 0.049261935 | 0.38622 | -2.059255472 |
| WBSCR22 | 79996 | AT | 0.002953318 | 0.011209812 | 0.00741871 | 0.11908 | -2.775790392 |
| LAT2 | 80065 | AT | 0.000723122 | 0.003931274 | 0.02088 | 0.1051 | -1.616120515 |
| SHFM1 | 80555 | AT | 0.007554962 | 0.023627262 | 0.010115484 | 0.07104 | -1.949175792 |
| FOXP2 | 81483 | AT | 0.011728315 | 0.032885639 | 0.002962092 | 0.02736 | -2.223186485 |
| CREB3L2 | 81912 | AT | 0.003656495 | 0.013208959 | 0.006075484 | 0.1774 | -3.374145434 |
| SLC37A3 | 81982 | AT | 0.000255838 | 0.001792962 | 0.001660645 | 0.01862 | -2.417030094 |
| DENND2A | 82008 | AT | 0.000237957 | 0.001709607 | 0.029988387 | 0.2699 | -2.19724131 |
| LMBR1 | 82468 | AT | 4.51E-05 | 0.000466258 | 0.022496774 | 0.1068 | -1.557585997 |
| ANGPT2 | 82577 | AT | 0.002442532 | 0.00969592 | 0.011352258 | 0.23752 | -3.040835158 |
| FAM86B1 | 82677 | AT | 0.001365042 | 0.006320231 | 0.026844516 | 0.21528 | -2.081877947 |
| SLC39A14 | 83008 | AT | 0.017419256 | 0.044129164 | 0.00057871 | 0.0341 | -4.076251732 |
| ADAM9 | 83482 | AT | 0.014552443 | 0.038542054 | 0.003626452 | 0.02596 | -1.968302239 |
| GINS4 | 83516 | AT | 0.000307952 | 0.002066144 | 0.052692258 | 0.6034 | -2.438111788 |
| IKBKB | 83590 | AT | 0.001071074 | 0.005259176 | 0.000501935 | 0.08514 | -5.133580646 |
| HGSNAT | 83762 | AT | 2.83E-05 | 0.000315802 | 0.049105161 | 0.22874 | -1.53862184 |
| RPS20 | 83884 | AT | 1.89E-09 | 6.40E-08 | 0.062521935 | 0.31304 | -1.610813515 |
| ASPH | 83966 | AT | 0.005611438 | 0.018662024 | 0.027603871 | 0.36822 | -2.59072457 |
| MCMDC2 | 84040 | AT | 0.010878545 | 0.031064723 | 0.001889677 | 0.07276 | -3.650760217 |
| TCEB1 | 84201 | AT | 0.000224454 | 0.001639121 | 0.002683871 | 0.0853 | -3.458914311 |
| DOCK8 | 85698 | AT | 0.00194456 | 0.008205134 | 0.009294194 | 0.18836 | -3.00896517 |
| DOCK8 | 85699 | AT | 0.000307073 | 0.002066144 | 0.00312129 | 0.0453 | -2.675060551 |
| SPATA6L | 85752 | AT | 0.005511367 | 0.0183593 | 0.070759868 | 0.37938 | -1.679246332 |
| PTPRD | 85841 | AT | 0.000249824 | 0.00177012 | 0.003185161 | 0.01606 | -1.61782878 |
| CLTA | 86327 | AT | 2.28E-05 | 0.000269431 | 0.003581935 | 0.02166 | -1.799563949 |
| PCSK5 | 86632 | AT | 0.00311371 | 0.011645849 | 0.030129677 | 0.15472 | -1.636106385 |
| NTRK2 | 86723 | AT | 0.007685651 | 0.023900236 | 0.022083226 | 0.13854 | -1.836340786 |
| NAA35 | 86742 | AT | 7.62E-08 | 2.00E-06 | 0.009055484 | 0.16016 | -2.872802789 |
| CTSL | 86775 | AT | 0.000988628 | 0.00496504 | 0.002849677 | 0.01674 | -1.770595263 |
| ZNF169 | 86925 | AT | 0.000245077 | 0.001743004 | 0.02584 | 0.14588 | -1.730860687 |
| TRIM14 | 87031 | AT | 0.00039139 | 0.002507963 | 0.04397987 | 0.37378 | -2.139935357 |
| SVEP1 | 87186 | AT | 0.010076378 | 0.029308086 | 0.022802158 | 0.26578 | -2.455813706 |
| PTGR1 | 87218 | AT | 0.000266999 | 0.00185599 | 0.010445806 | 0.28548 | -3.307971373 |
| RABEPK | 87552 | AT | 6.14E-05 | 0.000587554 | 0.017010968 | 0.11328 | -1.896004333 |
| RALGPS1 | 87611 | AT | 5.97E-06 | 9.16E-05 | 0.015275484 | 0.19212 | -2.531870995 |
| GBGT1 | 88019 | AT | 0.009866046 | 0.028815925 | 0.049392903 | 0.27448 | -1.715071644 |
| SARDH | 88079 | AT | 0.006896455 | 0.022014397 | 0.002116774 | 0.03252 | -2.731961962 |
| EHMT1 | 88340 | AT | 0.002711646 | 0.010494014 | 0.003005161 | 0.07538 | -3.222210747 |
| CXorf23 | 88649 | AT | 0.016959649 | 0.043306371 | 0.011093548 | 0.07382 | -1.895265985 |
| KIF4A | 89372 | AT | 5.50E-07 | 1.13E-05 | 0.002271613 | 0.8835 | -5.963401183 |
| MAGT1 | 89536 | AT | 0.002036571 | 0.008477283 | 0.008013548 | 0.17024 | -3.056075549 |
| SYTL4 | 89602 | AT | 0.000629302 | 0.003573892 | 0.015005161 | 0.10798 | -1.973551797 |
| COL4A6 | 89859 | AT | 0.000455175 | 0.002812583 | 0.056382 | 0.62304 | -2.402460764 |
| PHF6 | 90146 | AT | 0.000147865 | 0.001169122 | 0.017250323 | 0.09034 | -1.655749487 |
| ATP11C | 90242 | AT | 0.011069559 | 0.031454195 | 0.006292903 | 0.06918 | -2.397289277 |
| UBE4B | 593 | AT | 8.97E-12 | 4.32E-10 | 0.029490968 | 0.00652 | 1.509209662 |
| KIF1B | 601 | AT | 4.64E-56 | 5.97E-53 | 0.235153548 | 0.0237 | 2.29476365 |
| NIPAL3 | 1109 | AT | 3.27E-31 | 7.87E-29 | 0.059746452 | 0.00728 | 2.10497894 |
| MYCL | 1976 | AT | 6.25E-18 | 6.10E-16 | 0.0987 | 0.0166 | 1.782682251 |
| SLC1A7 | 3054 | AT | 0.00592928 | 0.019517002 | 0.011054676 | 0.0016 | 1.9328499 |
| WDR78 | 3327 | AT | 0.017084379 | 0.043523951 | 0.08100129 | 0.01718 | 1.550719168 |
| C1orf173 | 3463 | AT | 4.00E-13 | 2.34E-11 | 0.121800658 | 0.01834 | 1.89330129 |
| DNM3 | 9000 | AT | 1.99E-20 | 2.65E-18 | 0.061915484 | 0.0055 | 2.421022199 |
| DNM3 | 9001 | AT | 1.60E-09 | 5.46E-08 | 0.016084516 | 0.0028 | 1.748237661 |
| NFASC | 9493 | AT | 1.89E-28 | 3.31E-26 | 0.152323226 | 0.02172 | 1.947771254 |
| CNST | 10495 | AT | 4.30E-05 | 0.000447205 | 0.02832 | 0.00616 | 1.525491491 |
| ANK3 | 11847 | AT | 3.65E-10 | 1.47E-08 | 0.026822581 | 0.00356 | 2.019483549 |
| ZNF365 | 11878 | AT | 2.01E-24 | 3.23E-22 | 0.074544516 | 0.0041 | 2.900409505 |
| SPOCK2 | 12072 | AT | 1.28E-19 | 1.54E-17 | 0.011877419 | 0.00224 | 1.668163198 |
| SLC35G1 | 12569 | AT | 5.59E-08 | 1.52E-06 | 0.107686452 | 0.0076 | 2.651075531 |
| ATRNL1 | 13219 | AT | 2.95E-10 | 1.22E-08 | 0.094370588 | 0.01 | 2.244644366 |
| FAM53B | 13405 | AT | 0.003434068 | 0.012605702 | 0.002028387 | 0.00026 | 2.054314592 |
| TTC40 | 13507 | AT | 1.59E-11 | 7.61E-10 | 0.415057143 | 0.0638 | 1.872663014 |
| ZNF195 | 13962 | AT | 3.45E-11 | 1.57E-09 | 0.008483226 | 0.00046 | 2.914619569 |
| MICAL2 | 14417 | AT | 1.02E-46 | 9.83E-44 | 0.109403226 | 0.00654 | 2.81710321 |
| SAA2 | 14579 | AT | 2.21E-05 | 0.000262993 | 0.153953488 | 0.0323 | 1.561583303 |
| SAA2 | 14580 | AT | 3.50E-05 | 0.000375907 | 0.773510853 | 0.1071 | 1.977176723 |
| KIAA1549L | 14895 | AT | 6.62E-06 | 0.000100657 | 0.006312903 | 5.00E-04 | 2.53574285 |
| SDHAF2 | 16221 | AT | 0.00433721 | 0.015218731 | 0.008926452 | 0.00148 | 1.796976872 |
| STIP1 | 16569 | AT | 0.002005547 | 0.008379817 | 0.001736774 | 0.00028 | 1.824995157 |
| PELI3 | 17033 | AT | 9.42E-35 | 3.16E-32 | 0.136662581 | 0.0121 | 2.42430952 |
| NEU3 | 17813 | AT | 6.84E-08 | 1.82E-06 | 0.05917871 | 0.00846 | 1.94521267 |
| DLG2 | 18101 | AT | 1.79E-06 | 3.18E-05 | 0.010434839 | 0.00072 | 2.673654151 |
| WNK1 | 19614 | AT | 1.96E-05 | 0.000241187 | 0.003330968 | 5.00E-04 | 1.896410056 |
| PTPRO | 20576 | AT | 6.68E-13 | 3.73E-11 | 0.052187097 | 0.00356 | 2.685074731 |
| NELL2 | 21289 | AT | 0.000872421 | 0.004513756 | 0.001882581 | 0.00024 | 2.059759875 |
| TROAP | 21553 | AT | 0.001219397 | 0.005802673 | 0.494255195 | 0.08592 | 1.749635342 |
| SLC4A8 | 21857 | AT | 0.001629504 | 0.007198765 | 0.058849677 | 0.01284 | 1.522421054 |
| GNPTAB | 24002 | AT | 2.59E-08 | 7.25E-07 | 0.011538065 | 0.00242 | 1.561883987 |
| PITPNM2 | 25043 | AT | 7.89E-10 | 2.95E-08 | 0.091850968 | 0.01236 | 2.005701896 |
| DCLK1 | 25624 | AT | 1.19E-20 | 1.61E-18 | 0.283352903 | 0.04336 | 1.877155772 |
| MCF2L | 26324 | AT | 3.00E-14 | 1.98E-12 | 0.012052258 | 0.00214 | 1.728446205 |
| ESR2 | 27868 | AT | 0.000447363 | 0.002778988 | 0.088942636 | 0.0122 | 1.986555667 |
| SPTB | 27909 | AT | 1.06E-32 | 2.81E-30 | 0.321579739 | 0.02238 | 2.66507783 |
| RGS6 | 28205 | AT | 6.25E-08 | 1.68E-06 | 0.026305806 | 0.00178 | 2.693176328 |
| KLC1 | 29467 | AT | 0.000834073 | 0.004335719 | 0.51586 | 0.11438 | 1.506309172 |
| INF2 | 29545 | AT | 4.32E-16 | 3.50E-14 | 0.135630323 | 0.02324 | 1.764058036 |
| FMN1 | 29830 | AT | 0.000145017 | 0.001151326 | 0.010085806 | 0.00112 | 2.197800448 |
| TTBK2 | 30199 | AT | 1.13E-07 | 2.84E-06 | 0.039012903 | 0.00758 | 1.638379244 |
| PEAK1 | 31985 | AT | 0.000956516 | 0.004847982 | 0.00422 | 0.00088 | 1.5676685 |
| TM2D3 | 32767 | AT | 2.13E-40 | 1.49E-37 | 0.065707742 | 0.01072 | 1.813105601 |
| C16orf45 | 34155 | AT | 3.13E-05 | 0.000346321 | 0.00356 | 0.00072 | 1.598264612 |
| SYT17 | 34294 | AT | 5.63E-16 | 4.34E-14 | 0.161507097 | 0.02576 | 1.835726183 |
| CDH13 | 37779 | AT | 2.88E-07 | 6.39E-06 | 0.018492258 | 0.00242 | 2.033584621 |
| KIAA0513 | 37877 | AT | 1.76E-33 | 5.23E-31 | 0.074574839 | 0.00512 | 2.678648729 |
| DLG4 | 38847 | AT | 6.16E-09 | 1.92E-07 | 0.007999355 | 0.00118 | 1.913846455 |
| GLP2R | 39254 | AT | 0.000168388 | 0.001300704 | 0.033874775 | 0.00618 | 1.701352359 |
| DHRS11 | 40495 | AT | 0.003403169 | 0.012539962 | 0.016725806 | 0.00282 | 1.780215938 |
| ITGB3 | 42069 | AT | 0.001597682 | 0.007094776 | 0.145204516 | 0.02022 | 1.971470991 |
| GRIN2C | 43310 | AT | 6.45E-20 | 8.03E-18 | 0.226424837 | 0.0491 | 1.528554008 |
| RPTOR | 44080 | AT | 2.54E-12 | 1.30E-10 | 0.025909032 | 0.0043 | 1.795976621 |
| OSBPL1A | 44878 | AT | 5.76E-22 | 8.38E-20 | 0.027207742 | 0.00186 | 2.682925075 |
| PIK3C3 | 45318 | AT | 0.00670132 | 0.02154999 | 0.006154839 | 0.00116 | 1.668818549 |
| LIPG | 45488 | AT | 0.015157456 | 0.039705818 | 0.038627742 | 0.00814 | 1.557180541 |
| MBD2 | 45576 | AT | 1.70E-13 | 1.05E-11 | 0.068496774 | 0.00366 | 2.929323505 |
| MBP | 45844 | AT | 3.70E-07 | 8.05E-06 | 0.084337419 | 0.0137 | 1.817429817 |
| ZNF66 | 48727 | AT | 4.69E-17 | 4.07E-15 | 0.092628378 | 0.01418 | 1.876763036 |
| ZNF66 | 48728 | AT | 3.47E-16 | 2.88E-14 | 0.520579054 | 0.0632 | 2.108637456 |
| TSHZ3 | 48868 | AT | 0.000214886 | 0.001591311 | 0.017674839 | 0.00226 | 2.056777273 |
| ZNF527 | 49507 | AT | 0.00057574 | 0.003347196 | 0.150393548 | 0.03326 | 1.50890004 |
| DYRK1B | 49844 | AT | 3.79E-07 | 8.21E-06 | 0.028032903 | 0.00504 | 1.715972853 |
| ZNF283 | 50248 | AT | 4.22E-38 | 1.63E-35 | 0.507446452 | 0.0779 | 1.873965238 |
| ZSCAN18 | 52402 | AT | 0.008009723 | 0.024711247 | 0.002752258 | 0.00026 | 2.359495337 |
| MTA3 | 53367 | AT | 1.41E-19 | 1.64E-17 | 0.175455484 | 0.02204 | 2.074526373 |
| PRKCE | 53461 | AT | 1.82E-10 | 7.80E-09 | 0.035003871 | 0.00332 | 2.355493872 |
| MEIS1 | 53808 | AT | 0.000312611 | 0.002083627 | 0.001800645 | 2.00E-04 | 2.197582936 |
| AAK1 | 53858 | AT | 2.45E-05 | 0.000282924 | 0.585814839 | 0.09822 | 1.785793904 |
| ADD2 | 53902 | AT | 7.86E-70 | 3.03E-66 | 0.353303871 | 0.02858 | 2.514621338 |
| CD8B | 54449 | AT | 0.000275745 | 0.00190306 | 0.213355 | 0.04474 | 1.562089489 |
| LIMS3L | 54936 | AT | 1.78E-06 | 3.16E-05 | 0.428942105 | 0.030475 | 2.644415282 |
| CCDC148 | 55662 | AT | 4.47E-10 | 1.76E-08 | 0.506969118 | 0.10932 | 1.534170729 |
| GAD1 | 55907 | AT | 8.15E-39 | 3.31E-36 | 0.171494839 | 0.007 | 3.198643022 |
| GLS | 56590 | AT | 1.83E-30 | 4.02E-28 | 0.600427097 | 0.03118 | 2.957864366 |
| ICA1L | 56954 | AT | 1.24E-07 | 3.08E-06 | 0.021433548 | 0.00168 | 2.546163583 |
| PLEKHM3 | 57206 | AT | 1.39E-06 | 2.55E-05 | 0.102399219 | 0.01698 | 1.796842902 |
| DOCK10 | 57783 | AT | 0.000305603 | 0.002061152 | 0.018169677 | 0.00338 | 1.681878419 |
| NGEF | 58033 | AT | 0.008445564 | 0.025693313 | 0.005850649 | 0.00076 | 2.040989501 |
| AGAP1 | 58086 | AT | 6.09E-13 | 3.48E-11 | 0.012689677 | 0.00086 | 2.691611751 |
| NRSN2 | 58445 | AT | 1.18E-15 | 8.62E-14 | 0.011320645 | 0.00204 | 1.713678256 |
| PLCB1 | 58673 | AT | 0.001371869 | 0.006328985 | 0.007622581 | 0.00136 | 1.72363028 |
| ZBP1 | 59942 | AT | 0.000730189 | 0.003950193 | 0.0634976 | 0.00914 | 1.938341724 |
| KCNQ2 | 60120 | AT | 0.000102485 | 0.000875903 | 0.892152632 | 0.13078 | 1.920120707 |
| MYT1 | 60198 | AT | 2.01E-13 | 1.21E-11 | 0.158523179 | 0.01064 | 2.701280337 |
| ITGB2 | 60849 | AT | 1.47E-09 | 5.22E-08 | 0.027576774 | 0.00082 | 3.515424842 |
| MAPK1 | 61257 | AT | 1.51E-12 | 8.04E-11 | 0.073296129 | 0.01332 | 1.705241132 |
| SYNGR1 | 62298 | AT | 1.29E-08 | 3.92E-07 | 0.255556774 | 0.0257 | 2.296953601 |
| TRAIP | 64923 | AT | 0.010113541 | 0.029365458 | 0.068009032 | 0.01258 | 1.687532272 |
| FOXP1 | 65602 | AT | 6.72E-11 | 2.96E-09 | 0.023963871 | 0.00116 | 3.028127315 |
| KALRN | 66523 | AT | 6.91E-06 | 0.000104428 | 0.644205161 | 0.124 | 1.647735683 |
| KALRN | 66525 | AT | 8.20E-05 | 0.000731012 | 0.004432903 | 0.00082 | 1.687505664 |
| NPHP3 | 66809 | AT | 0.001145283 | 0.005520549 | 0.017290323 | 0.00366 | 1.552683809 |
| PLCH1 | 67348 | AT | 0.000915911 | 0.004682202 | 0.019609804 | 0.00372 | 1.662305973 |
| ABCC5 | 67813 | AT | 4.65E-21 | 6.52E-19 | 0.051429032 | 0.00614 | 2.125378101 |
| EHHADH | 67979 | AT | 0.00040123 | 0.002552049 | 0.012694194 | 0.00172 | 1.998820397 |
| ZFYVE28 | 68560 | AT | 1.01E-18 | 1.13E-16 | 0.033075484 | 0.00322 | 2.32941098 |
| ABLIM2 | 68745 | AT | 1.28E-39 | 6.60E-37 | 0.384285806 | 0.04234 | 2.205654298 |
| GPR125 | 68941 | AT | 1.94E-13 | 1.19E-11 | 0.017284516 | 0.00258 | 1.90202168 |
| PCDH7 | 68993 | AT | 9.73E-11 | 4.24E-09 | 0.147350746 | 0.01396 | 2.356619676 |
| CEP135 | 69338 | AT | 0.002143998 | 0.008819681 | 0.10515871 | 0.01442 | 1.986854598 |
| RUFY3 | 69449 | AT | 3.35E-40 | 2.15E-37 | 0.318575484 | 0.0535 | 1.784177791 |
| FAM13A | 69905 | AT | 5.03E-40 | 2.98E-37 | 0.133437419 | 0.01956 | 1.920145934 |
| FAM13A | 69909 | AT | 7.96E-07 | 1.55E-05 | 0.049256774 | 0.00948 | 1.647862589 |
| MYO10 | 71604 | AT | 6.67E-12 | 3.27E-10 | 0.003274194 | 0.00044 | 2.007052146 |
| RANBP3L | 71811 | AT | 4.99E-05 | 0.000502367 | 0.012144516 | 0.00106 | 2.438608812 |
| MCTP1 | 72801 | AT | 1.03E-05 | 0.000146027 | 0.033143871 | 0.00522 | 1.84836041 |
| TRIM36 | 73020 | AT | 2.59E-07 | 5.84E-06 | 0.352461935 | 0.07696 | 1.521656826 |
| C5orf63 | 73181 | AT | 1.27E-35 | 4.66E-33 | 0.199529032 | 0.01712 | 2.45571238 |
| C5orf63 | 73182 | AT | 4.21E-24 | 6.36E-22 | 0.131166452 | 0.01186 | 2.403295746 |
| MEGF10 | 73186 | AT | 2.68E-16 | 2.28E-14 | 0.088263226 | 0.0129 | 1.923096241 |
| FBN2 | 73199 | AT | 0.001017494 | 0.005067092 | 0.166602027 | 0.02262 | 1.996773426 |
| ACSL6 | 73248 | AT | 5.79E-10 | 2.22E-08 | 0.050840645 | 0.00316 | 2.778124109 |
| PCDH1 | 73832 | AT | 0.000160563 | 0.001252165 | 0.474151613 | 0.09184 | 1.641479196 |
| SYNPO | 74104 | AT | 9.04E-29 | 1.74E-26 | 0.788934194 | 0.08208 | 2.262988531 |
| KCNIP1 | 74492 | AT | 0.010695273 | 0.030701944 | 0.002987097 | 0.00056 | 1.674120432 |
| NSG2 | 74597 | AT | 0.00343554 | 0.012605702 | 0.004936129 | 0.00072 | 1.925085494 |
| PHACTR1 | 75367 | AT | 1.16E-12 | 6.31E-11 | 0.031565161 | 0.00264 | 2.481275104 |
| DAAM2 | 76055 | AT | 1.00E-17 | 9.31E-16 | 0.034610323 | 0.00284 | 2.500347926 |
| HMGCLL1 | 76534 | AT | 3.00E-18 | 3.08E-16 | 0.179370992 | 0.03594 | 1.607605363 |
| DST | 76558 | AT | 0.000210272 | 0.001563995 | 0.004601935 | 0.00058 | 2.071204148 |
| DST | 76559 | AT | 3.55E-10 | 1.44E-08 | 0.009893548 | 0.00204 | 1.578933059 |
| AHI1 | 77889 | AT | 6.21E-05 | 0.000593545 | 0.011380645 | 0.00132 | 2.154282383 |
| AHI1 | 77890 | AT | 3.02E-21 | 4.31E-19 | 0.078794194 | 0.01618 | 1.583063397 |
| PCMT1 | 78107 | AT | 3.76E-30 | 7.82E-28 | 0.084029677 | 0.01698 | 1.599133858 |
| TMEM242 | 78240 | AT | 1.94E-18 | 2.05E-16 | 0.070728387 | 0.0063 | 2.418297374 |
| SOD2 | 78301 | AT | 1.31E-07 | 3.20E-06 | 0.150743871 | 0.02368 | 1.850951368 |
| URGCP | 79357 | AT | 2.23E-09 | 7.48E-08 | 0.027447742 | 0.0043 | 1.853668881 |
| EGFR | 79742 | AT | 1.58E-05 | 0.000206378 | 0.004387097 | 0.00046 | 2.255196471 |
| PCLO | 80261 | AT | 1.29E-17 | 1.16E-15 | 0.185030405 | 0.02338 | 2.068639209 |
| CCDC132 | 80457 | AT | 3.05E-10 | 1.26E-08 | 0.00858 | 0.00052 | 2.803360381 |
| DYNC1I1 | 80543 | AT | 2.00E-11 | 9.36E-10 | 0.018320645 | 0.00134 | 2.615358961 |
| BCAP29 | 81360 | AT | 5.67E-14 | 3.70E-12 | 0.057985161 | 0.0121 | 1.566981685 |
| LAMB4 | 81393 | AT | 6.27E-10 | 2.39E-08 | 0.283204762 | 0.01386 | 3.017163183 |
| NRCAM | 81398 | AT | 8.05E-08 | 2.10E-06 | 0.009972903 | 0.00098 | 2.320074445 |
| FOXP2 | 81480 | AT | 1.30E-09 | 4.72E-08 | 0.07403268 | 0.0061 | 2.496217843 |
| CLEC5A | 82057 | AT | 0.001732535 | 0.007541672 | 0.127076774 | 0.02728 | 1.538637592 |
| TNFRSF10C | 83065 | AT | 0.000226337 | 0.001649178 | 0.120732895 | 0.01426 | 2.136122209 |
| DOCK5 | 83104 | AT | 1.18E-05 | 0.000163782 | 0.036297419 | 0.00742 | 1.587567589 |
| AP3M2 | 83558 | AT | 2.59E-06 | 4.40E-05 | 0.031714839 | 0.00414 | 2.036088882 |
| TPD52 | 84271 | AT | 6.85E-27 | 1.12E-24 | 0.041343871 | 0.00634 | 1.875045419 |
| ZFAND1 | 84303 | AT | 0.00289471 | 0.011042779 | 0.011160645 | 0.00188 | 1.781121989 |
| ADAMTSL1 | 85949 | AT | 0.001606011 | 0.007123554 | 0.069603311 | 0.01092 | 1.852216171 |
| FRMPD1 | 86421 | AT | 0.001032978 | 0.00512434 | 0.032045695 | 0.00662 | 1.577067494 |
| TRPM3 | 86558 | AT | 5.09E-08 | 1.39E-06 | 0.020259477 | 0.0011 | 2.91331251 |
| TRPM3 | 86559 | AT | 2.21E-06 | 3.80E-05 | 0.020117647 | 0.00314 | 1.857374593 |
| TRPM3 | 86561 | AT | 5.50E-33 | 1.57E-30 | 0.263507843 | 0.00234 | 4.723932203 |
| RASEF | 86677 | AT | 1.49E-28 | 2.73E-26 | 0.258609677 | 0.00944 | 3.310363908 |
| SHC3 | 86795 | AT | 1.36E-06 | 2.51E-05 | 0.018638158 | 0.00296 | 1.840021711 |
| EPB41L4B | 87163 | AT | 3.96E-18 | 3.96E-16 | 0.187520915 | 0.01324 | 2.650647836 |
| RC3H2 | 87485 | AT | 1.08E-07 | 2.74E-06 | 0.045701935 | 0.00638 | 1.968972552 |
| RALGPS1 | 87609 | AT | 3.21E-16 | 2.69E-14 | 0.038975484 | 0.0025 | 2.746642098 |
| ARHGAP6 | 88478 | AT | 0.014639701 | 0.038729393 | 0.015726452 | 0.00334 | 1.549373304 |
| ACOT9 | 88691 | AT | 0.000218293 | 0.001607989 | 0.059303871 | 0.01198 | 1.599435989 |
| SYP | 89105 | AT | 7.66E-18 | 7.29E-16 | 0.034852903 | 0.00632 | 1.707417232 |
| EDA | 89363 | AT | 5.28E-05 | 0.000524273 | 0.130918065 | 0.01418 | 2.222739145 |
| KIF4A | 89373 | AT | 5.50E-07 | 1.13E-05 | 0.997728387 | 0.1165 | 2.147589809 |
| SEPT6 | 89964 | AT | 1.40E-62 | 2.69E-59 | 0.465245806 | 0.06144 | 2.024504793 |
| SEPT6 | 89965 | AT | 1.36E-18 | 1.47E-16 | 0.031167097 | 0.0045 | 1.935285551 |
| GRIA3 | 90012 | AT | 9.45E-08 | 2.43E-06 | 0.054563226 | 0.00938 | 1.760780373 |
| GRIA3 | 90014 | AT | 9.07E-05 | 0.000789861 | 0.043623226 | 0.00788 | 1.711261807 |
| IDS | 90292 | AT | 4.58E-20 | 5.88E-18 | 0.113197419 | 0.01956 | 1.755646704 |
| RBP7 | 592 | ES | 0.009642097 | 0.047307758 | 0.017281132 | 0.28316 | -2.796396834 |
| KIF1B | 606 | ES | 1.42E-05 | 0.000311455 | 0.161377444 | 0.85758 | -1.670368478 |
| KIF1B | 607 | ES | 0.007014402 | 0.038247755 | 0.072836885 | 0.6951 | -2.255833228 |
| TARDBP | 635 | ES | 0.000840309 | 0.008297184 | 0.010305161 | 0.17748 | -2.846213061 |
| EXOSC10 | 647 | ES | 0.000727054 | 0.00737006 | 0.011198065 | 0.37824 | -3.519787962 |
| ARHGEF10L | 862 | ES | 0.002980413 | 0.021025007 | 0.054854902 | 0.5144 | -2.23830962 |
| UBR4 | 887 | ES | 0.008491402 | 0.043611631 | 0.034618447 | 0.55118 | -2.767674756 |
| EIF4G3 | 959 | ES | 0.000553529 | 0.005977878 | 0.0316184 | 0.71646 | -3.120583189 |
| RAP1GAP | 991 | ES | 0.000301256 | 0.003684935 | 0.074494631 | 0.4626 | -1.826135696 |
| RAP1GAP | 992 | ES | 0.001574144 | 0.013184259 | 0.114218919 | 0.53506 | -1.544261942 |
| KDM1A | 1030 | ES | 0.00870533 | 0.044286709 | 0.013900645 | 0.36474 | -3.267249517 |
| FAM76A | 1344 | ES | 0.000621987 | 0.006566246 | 0.054858696 | 0.56072 | -2.324460963 |
| SNRNP40 | 1459 | ES | 0.000154622 | 0.002184614 | 0.033657419 | 0.18084 | -1.681379145 |
| OSCP1 | 1780 | ES | 0.005336606 | 0.032051615 | 0.030657616 | 0.39428 | -2.554180206 |
| MEAF6 | 1805 | ES | 0.000701371 | 0.007192158 | 0.103231613 | 0.85546 | -2.114664203 |
| PTPRF | 2189 | ES | 0.009205593 | 0.045854101 | 0.136124183 | 0.69814 | -1.634852076 |
| PTPRF | 2190 | ES | 0.006563773 | 0.0365321 | 0.056309524 | 0.72094 | -2.549692233 |
| MKNK1 | 2814 | ES | 1.57E-05 | 0.000337029 | 0.126064286 | 0.6581 | -1.652564915 |
| SGIP1 | 3316 | ES | 0.001471348 | 0.012546064 | 0.097583333 | 0.7939 | -2.096250795 |
| FPGT | 3452 | ES | 0.003227108 | 0.022184215 | 0.003045033 | 0.1914 | -4.140853704 |
| LPHN2 | 3565 | ES | 7.54E-11 | 5.13E-09 | 0.075258667 | 0.94452 | -2.529745793 |
| LPHN2 | 3570 | ES | 0.009842365 | 0.048039049 | 0.016840816 | 0.2774 | -2.801655025 |
| LPHN2 | 3571 | ES | 1.86E-09 | 1.03E-07 | 0.184423776 | 0.94942 | -1.638615031 |
| LPHN2 | 3573 | ES | 0.000233086 | 0.003010298 | 0.026272115 | 0.852875 | -3.48010487 |
| PRKACB | 3592 | ES | 0.00968993 | 0.047464556 | 0.087441818 | 0.65438 | -2.012714585 |
| PRKACB | 3593 | ES | 0.010410797 | 0.04998579 | 0.0630125 | 0.7067 | -2.417273128 |
| RPL5 | 3768 | ES | 0.004837142 | 0.029789738 | 0.000883871 | 0.00608 | -1.928448887 |
| FNBP1L | 3790 | ES | 0.001171924 | 0.010581103 | 0.011994631 | 0.7153 | -4.088242913 |
| DPYD | 3838 | ES | 0.001773355 | 0.014256068 | 0.068216406 | 0.900075 | -2.579792996 |
| PDE4DIP | 4420 | ES | 0.000415027 | 0.004795625 | 0.070348571 | 0.79244 | -2.421654316 |
| VPS45 | 7421 | ES | 0.000404079 | 0.004705459 | 0.013593548 | 0.1228 | -2.200961719 |
| RFX5 | 7609 | ES | 0.000367128 | 0.004352548 | 0.006868831 | 0.24296 | -3.565902865 |
| SELENBP1 | 7620 | ES | 0.004280086 | 0.027291224 | 0.015769065 | 0.07352 | -1.539507385 |
| CCT3 | 8239 | ES | 0.001084699 | 0.01004081 | 0.007151613 | 0.10068 | -2.644609258 |
| MEF2D | 8275 | ES | 0.003640854 | 0.0241655 | 0.091455921 | 0.81808 | -2.191103012 |
| NOS1AP | 8739 | ES | 0.000501928 | 0.005552229 | 0.028768293 | 0.44136 | -2.730587037 |
| DNM3 | 9003 | ES | 0.009688843 | 0.047464556 | 0.100971296 | 0.74652 | -2.000586127 |
| CAMSAP2 | 9338 | ES | 0.002693119 | 0.019464258 | 0.056265079 | 0.7103 | -2.535613333 |
| KDM5B | 9420 | ES | 0.003405712 | 0.023060366 | 0.1117875 | 0.51992 | -1.537075206 |
| ATP2B4 | 9450 | ES | 0.000143122 | 0.002058897 | 0.149751299 | 0.85524 | -1.742406222 |
| NFASC | 9507 | ES | 0.003636648 | 0.024158074 | 0.047810714 | 0.75712 | -2.762271999 |
| NFASC | 9512 | ES | 0.003459914 | 0.023279995 | 0.026694964 | 0.63018 | -3.161530558 |
| NFASC | 9515 | ES | 0.000177017 | 0.002423579 | 0.120045324 | 0.79586 | -1.891553922 |
| TP53BP2 | 9930 | ES | 0.000445154 | 0.005039158 | 0.153756849 | 0.7106 | -1.53073723 |
| CDC42BPA | 10048 | ES | 0.00277783 | 0.019959465 | 0.01580566 | 0.40036 | -3.231996015 |
| SIPA1L2 | 10315 | ES | 0.00422442 | 0.027046725 | 0.051052597 | 0.67744 | -2.585464565 |
| CELF2 | 10743 | ES | 0.009099714 | 0.045627597 | 0.00345 | 0.1036 | -3.402163099 |
| FRMD4A | 10815 | ES | 0.000784462 | 0.007872371 | 0.129784967 | 0.70838 | -1.697101689 |
| ITGB1 | 11188 | ES | 0.001223445 | 0.010945186 | 0.011843226 | 0.05874 | -1.601364884 |
| NCOA4 | 11549 | ES | 0.004621042 | 0.02882328 | 0.00601634 | 0.0333 | -1.711078318 |
| DNAJC12 | 11904 | ES | 9.49E-05 | 0.00146797 | 0.003028188 | 0.06586 | -3.079566883 |
| HERC4 | 11916 | ES | 0.000603642 | 0.006404059 | 0.119790476 | 0.71768 | -1.790279602 |
| ANXA7 | 12145 | ES | 2.16E-05 | 0.000439217 | 0.062386452 | 0.7308 | -2.460791694 |
| CAMK2G | 12248 | ES | 0.00964163 | 0.047307758 | 0.048810458 | 0.2482 | -1.62629029 |
| CAMK2G | 12249 | ES | 0.001338627 | 0.011679389 | 0.04863961 | 0.422 | -2.160567086 |
| KCNMA1 | 12286 | ES | 6.77E-07 | 2.27E-05 | 0.090913235 | 0.86146 | -2.248723031 |
| SORBS1 | 12622 | ES | 0.000374673 | 0.004421081 | 0.014073684 | 0.3011 | -3.06313575 |
| SORBS1 | 12627 | ES | 0.003232922 | 0.022212301 | 0.02111938 | 0.33358 | -2.759691622 |
| SORBS1 | 12629 | ES | 0.002888452 | 0.020559704 | 0.012046207 | 0.631475 | -3.959308523 |
| SORBS1 | 12630 | ES | 0.000113248 | 0.001698053 | 0.017717361 | 0.79328 | -3.801631236 |
| ZFYVE27 | 12737 | ES | 0.002726784 | 0.019669743 | 0.069539869 | 0.7397 | -2.364344452 |
| SLK | 13030 | ES | 0.007529296 | 0.040024012 | 0.020504286 | 0.12554 | -1.811990509 |
| TACC2 | 13344 | ES | 0.001651569 | 0.013553335 | 0.039442742 | 0.56476 | -2.661550813 |
| TUBGCP2 | 13534 | ES | 0.009732557 | 0.047626799 | 0.006809091 | 0.03682 | -1.687782558 |
| TALDO1 | 13738 | ES | 0.000124149 | 0.001829731 | 0.009936774 | 0.05876 | -1.777218911 |
| CHID1 | 13814 | ES | 0.00071523 | 0.007289524 | 0.00108 | 0.08906 | -4.412349259 |
| ZNF195 | 13979 | ES | 0.007464103 | 0.039858099 | 0.074513907 | 0.61218 | -2.106040574 |
| STIM1 | 14032 | ES | 0.000247995 | 0.003160043 | 0.01259404 | 0.13288 | -2.3562228 |
| APBB1 | 14118 | ES | 0.010107962 | 0.048969703 | 0.128189032 | 0.7499 | -1.766433875 |
| LDHA | 14636 | ES | 0.008224011 | 0.042705201 | 0.003196774 | 0.09328 | -3.373463486 |
| ELP4 | 14825 | ES | 0.00011461 | 0.001716074 | 0.01838129 | 0.32298 | -2.866257085 |
| LDLRAD3 | 15419 | ES | 5.12E-05 | 0.000895848 | 0.148803226 | 0.70312 | -1.552902773 |
| ATG13 | 15582 | ES | 8.45E-38 | 5.78E-35 | 0.083 | 0.915 | -2.400083457 |
| ATG13 | 15583 | ES | 9.25E-27 | 2.67E-24 | 0.197271613 | 0.94382 | -1.565353945 |
| MADD | 15717 | ES | 0.001082204 | 0.010036258 | 0.158815789 | 0.8664 | -1.696601722 |
| MADD | 15721 | ES | 0.000271583 | 0.00341649 | 0.14795 | 0.82316 | -1.716276214 |
| LPXN | 16011 | ES | 0.001316776 | 0.011535605 | 0.021320915 | 0.31466 | -2.691804173 |
| RTN3 | 16530 | ES | 0.009743496 | 0.047664816 | 0.012096129 | 0.21528 | -2.87905402 |
| RTN3 | 16533 | ES | 0.000866858 | 0.008489558 | 0.025227097 | 0.15576 | -1.820397675 |
| DPF2 | 16815 | ES | 4.76E-06 | 0.000119712 | 0.069589032 | 0.77382 | -2.408732316 |
| YIF1A | 17009 | ES | 0.005279234 | 0.03178323 | 0.005062581 | 0.04334 | -2.147199634 |
| PPP6R3 | 17311 | ES | 0.009348884 | 0.046230846 | 0.019471613 | 0.47524 | -3.194862283 |
| PPFIA1 | 17394 | ES | 5.93E-06 | 0.000145414 | 0.124735526 | 0.681 | -1.6973666 |
| RNF121 | 17462 | ES | 0.003134477 | 0.021760956 | 0.063169737 | 0.42088 | -1.896522418 |
| AAMDC | 17980 | ES | 0.000671127 | 0.006938241 | 0.114466452 | 0.58164 | -1.625569919 |
| USP28 | 18805 | ES | 6.90E-06 | 0.000165978 | 0.11847973 | 0.83646 | -1.954436812 |
| PHLDB1 | 19036 | ES | 0.005874317 | 0.034043409 | 0.069503226 | 0.47304 | -1.917806786 |
| PHLDB1 | 19041 | ES | 1.49E-05 | 0.000323181 | 0.152998701 | 0.7923 | -1.644510675 |
| SLC37A4 | 19074 | ES | 1.64E-06 | 4.85E-05 | 0.133149032 | 0.85538 | -1.86007677 |
| GRAMD1B | 19205 | ES | 3.75E-06 | 9.78E-05 | 0.069884211 | 0.80978 | -2.449922869 |
| ERC1 | 19656 | ES | 1.13E-05 | 0.000254669 | 0.051044444 | 0.794525 | -2.745047739 |
| ERC1 | 19658 | ES | 4.75E-05 | 0.000838758 | 0.124606452 | 0.781725 | -1.836342633 |
| NOP2 | 19890 | ES | 0.000102811 | 0.001564325 | 0.014853289 | 0.51382 | -3.543651655 |
| C2CD5 | 20735 | ES | 0.001649614 | 0.013553335 | 0.021545 | 0.41196 | -2.950782486 |
| C2CD5 | 20736 | ES | 0.004111671 | 0.026471415 | 0.011749265 | 0.44508 | -3.634463381 |
| PPFIBP1 | 20894 | ES | 0.004188336 | 0.026895877 | 0.047744538 | 0.6666 | -2.636325497 |
| DNM1L | 21060 | ES | 0.009102452 | 0.045627597 | 0.013286 | 0.12716 | -2.258735287 |
| DNM1L | 21061 | ES | 0.00109606 | 0.010102437 | 0.050044 | 0.68384 | -2.614821354 |
| PPHLN1 | 21226 | ES | 0.00475372 | 0.029444857 | 0.012334839 | 0.11758 | -2.254691279 |
| TWF1 | 21275 | ES | 0.008615511 | 0.044020887 | 0.006656774 | 0.34686 | -3.953286228 |
| CERS5 | 21659 | ES | 6.93E-05 | 0.001129409 | 0.088598065 | 0.68574 | -2.046388535 |
| CERS5 | 21660 | ES | 2.29E-05 | 0.000461715 | 0.088820645 | 0.71202 | -2.081486887 |
| CERS5 | 21661 | ES | 0.002292498 | 0.017353031 | 0.035080645 | 0.42792 | -2.501286704 |
| CERS5 | 21681 | ES | 0.000150518 | 0.002138699 | 0.058738961 | 0.5388 | -2.216241206 |
| CERS5 | 21684 | ES | 0.002507802 | 0.018471231 | 0.014197403 | 0.1336 | -2.241791229 |
| CS | 22418 | ES | 0.003463851 | 0.023288044 | 0.006036774 | 0.18142 | -3.40294499 |
| R3HDM2 | 22572 | ES | 0.006689622 | 0.036986054 | 0.04855098 | 0.58776 | -2.493704313 |
| R3HDM2 | 22573 | ES | 0.007393008 | 0.039635907 | 0.053653333 | 0.63286 | -2.46770563 |
| DCTN2 | 22643 | ES | 0.008922155 | 0.045052789 | 0.032692903 | 0.5119 | -2.750971266 |
| LRIG3 | 22772 | ES | 0.005500545 | 0.032696598 | 0.082682407 | 0.414433333 | -1.611905274 |
| MON2 | 22840 | ES | 0.008195659 | 0.042602122 | 0.026441026 | 0.56346 | -3.059179541 |
| CPSF6 | 23307 | ES | 0.006100406 | 0.034828521 | 0.001431818 | 0.0309 | -3.071811091 |
| NAP1L1 | 23480 | ES | 0.000109942 | 0.001659399 | 0.126841304 | 0.7024 | -1.711566309 |
| NAP1L1 | 23483 | ES | 0.007793525 | 0.041022431 | 0.002685806 | 0.04132 | -2.733365607 |
| ATP2B1 | 23647 | ES | 3.66E-05 | 0.000679883 | 0.087 | 0.90122 | -2.337841282 |
| ACTR6 | 23910 | ES | 2.84E-05 | 0.000553466 | 0.013520261 | 0.06568 | -1.580605058 |
| CHPT1 | 23996 | ES | 0.009252401 | 0.045919888 | 0.010341558 | 0.25566 | -3.207677859 |
| CCDC53 | 24023 | ES | 0.000303409 | 0.003703021 | 7.55E-05 | 0.07116 | -6.848767138 |
| C12orf23 | 24179 | ES | 0.000694572 | 0.007127303 | 0.020152258 | 0.18066 | -2.193300467 |
| MMAB | 24322 | ES | 1.72E-06 | 5.05E-05 | 0.009961935 | 0.32284 | -3.478385466 |
| MMAB | 24323 | ES | 0.001502867 | 0.012742583 | 0.006325161 | 0.11574 | -2.906810761 |
| GIT2 | 24393 | ES | 7.39E-11 | 5.05E-09 | 0.034674265 | 0.6503 | -2.931436034 |
| TCTN1 | 24478 | ES | 0.003193249 | 0.022050577 | 0.085097561 | 0.61228 | -1.97339132 |
| TCTN1 | 24483 | ES | 0.001663954 | 0.013631729 | 0.00814902 | 0.15658 | -2.955669435 |
| TRAFD1 | 24579 | ES | 0.000976648 | 0.009286496 | 0.006462914 | 0.2902 | -3.804490055 |
| SDSL | 24644 | ES | 0.000124803 | 0.001837574 | 0.0178875 | 0.34188 | -2.950357653 |
| C12orf43 | 24819 | ES | 0.002019607 | 0.015732964 | 0.106328571 | 0.56212 | -1.66518132 |
| CAMKK2 | 24853 | ES | 0.007657445 | 0.040533231 | 0.099777483 | 0.7703 | -2.043837508 |
| DCLK1 | 25625 | ES | 6.38E-06 | 0.000155807 | 0.119017931 | 0.55422 | -1.538287557 |
| LRCH1 | 25843 | ES | 0.006619266 | 0.036732164 | 0.028821277 | 0.56255 | -2.971366134 |
| TRIM13 | 25919 | ES | 0.000730938 | 0.007394472 | 0.012096104 | 0.16508 | -2.613546796 |
| MBNL2 | 26140 | ES | 0.007526477 | 0.040023179 | 0.094357252 | 0.73208 | -2.048801667 |
| DOCK9 | 26177 | ES | 0.000248156 | 0.003160043 | 0.093356618 | 0.82902 | -2.183817522 |
| ARHGEF7 | 26285 | ES | 0.001194406 | 0.010758244 | 0.073089677 | 0.62078 | -2.139289607 |
| ARHGEF7 | 26293 | ES | 0.005792357 | 0.033776766 | 0.01105303 | 0.1225 | -2.405406404 |
| CCNB1IP1 | 26422 | ES | 0.001848503 | 0.014664139 | 0.01403961 | 0.3001 | -3.062233104 |
| NUBPL | 27126 | ES | 0.001516953 | 0.012835547 | 0.000906767 | 0.06438 | -4.26267287 |
| GEMIN2 | 27356 | ES | 0.000142564 | 0.002052845 | 0.00788961 | 0.27122 | -3.537383546 |
| FKBP3 | 27404 | ES | 0.000158829 | 0.002237742 | 0.004025161 | 0.14242 | -3.566215456 |
| MIS18BP1 | 27410 | ES | 0.006089138 | 0.034805329 | 0.026266667 | 0.392475 | -2.704172133 |
| NIN | 27494 | ES | 0.00394893 | 0.025599076 | 0.038346479 | 0.62074 | -2.784249607 |
| TRIM9 | 27507 | ES | 0.009221795 | 0.045858831 | 0.022495425 | 0.56842 | -3.229548634 |
| CNIH1 | 27581 | ES | 2.02E-56 | 1.52E-52 | 0.120546218 | 0.973 | -2.088350847 |
| ACTR10 | 27682 | ES | 0.000162366 | 0.002273738 | 0.003384516 | 0.27154 | -4.384898508 |
| DAAM1 | 27733 | ES | 0.002416582 | 0.018029022 | 0.04807874 | 0.47222 | -2.284604892 |
| SLC38A6 | 27793 | ES | 0.000616084 | 0.006508499 | 0.131820261 | 0.81368 | -1.820127829 |
| ACTN1 | 28116 | ES | 3.21E-05 | 0.00061374 | 0.019371613 | 0.81944 | -3.744812438 |
| SIPA1L1 | 28199 | ES | 0.006238682 | 0.035364291 | 0.118895302 | 0.68378 | -1.749392938 |
| ZNF410 | 28328 | ES | 1.82E-05 | 0.000378591 | 0.031829677 | 0.24418 | -2.037506551 |
| MLH3 | 28470 | ES | 0.000407354 | 0.00473416 | 0.01569187 | 0.5935 | -3.632894475 |
| TTC8 | 28787 | ES | 0.006591157 | 0.03665567 | 0.038265772 | 0.43294 | -2.42604334 |
| TTC8 | 28788 | ES | 0.000533574 | 0.005812476 | 0.009051678 | 0.06482 | -1.968664059 |
| TTC7B | 28826 | ES | 0.005469691 | 0.032590516 | 0.047047368 | 0.27182 | -1.75398515 |
| FBLN5 | 28893 | ES | 0.001012573 | 0.009551008 | 0.043511565 | 0.61624 | -2.650609742 |
| MTA1 | 29643 | ES | 0.007250914 | 0.039101487 | 0.066011688 | 0.64558 | -2.280317316 |
| MTA1 | 29644 | ES | 1.66E-33 | 7.55E-31 | 0.206604348 | 0.9823 | -1.55909116 |
| APBA2 | 29758 | ES | 0.00192432 | 0.015137785 | 0.090725161 | 0.81406 | -2.194199342 |
| PPIP5K1 | 30267 | ES | 0.000173997 | 0.002393136 | 0.028787407 | 0.34672 | -2.488579489 |
| NPTN | 31606 | ES | 0.00018022 | 0.002458485 | 0.076270323 | 0.46112 | -1.799374407 |
| SNUPN | 31881 | ES | 1.93E-16 | 2.35E-14 | 0.189931193 | 0.92074 | -1.578515832 |
| SNUPN | 31883 | ES | 0.001216052 | 0.010907465 | 0.0316625 | 0.41748 | -2.579103624 |
| LRRC28 | 32637 | ES | 0.009770609 | 0.047780901 | 0.022379355 | 0.22564 | -2.310801934 |
| MEF2A | 32715 | ES | 2.43E-05 | 0.000484538 | 0.093943137 | 0.78366 | -2.121285576 |
| GNPTG | 33076 | ES | 0.004692533 | 0.029125865 | 0.012612903 | 0.09932 | -2.063626605 |
| TBC1D24 | 33273 | ES | 2.12E-05 | 0.000430857 | 0.055200758 | 0.85374 | -2.73865002 |
| CORO7 | 33672 | ES | 0.004889059 | 0.030003824 | 0.012281579 | 0.10032 | -2.100264584 |
| DNAJA3 | 33726 | ES | 2.48E-09 | 1.33E-07 | 0.030386364 | 0.26032 | -2.1479177 |
| ABCC1 | 34211 | ES | 0.001574253 | 0.013184259 | 0.02635 | 0.867775 | -3.494464189 |
| TNRC6A | 35650 | ES | 9.70E-05 | 0.001494608 | 0.062097857 | 0.42566 | -1.924929424 |
| CLN3 | 35719 | ES | 0.000368405 | 0.004364249 | 0.007414839 | 0.10176 | -2.619133876 |
| CCDC101 | 35755 | ES | 0.001262405 | 0.01117652 | 0.018709091 | 0.16982 | -2.205729502 |
| SULT1A1 | 35819 | ES | 0.005898061 | 0.034123004 | 0.033222759 | 0.1531 | -1.527856161 |
| RABEP2 | 35894 | ES | 0.000730187 | 0.007391848 | 0.012393421 | 0.3334 | -3.292177199 |
| CYLD | 36395 | ES | 0.004727105 | 0.029292071 | 0.018146099 | 0.4311 | -3.167884459 |
| DDX19A | 37382 | ES | 0.000249139 | 0.003165937 | 0.027220779 | 0.44062 | -2.784202202 |
| KIAA0513 | 37879 | ES | 5.27E-06 | 0.000130884 | 0.1938125 | 0.87114 | -1.502911502 |
| COX4I1 | 37910 | ES | 0.000839235 | 0.008294685 | 0.003703226 | 0.10706 | -3.364185146 |
| KLHDC4 | 37961 | ES | 0.001600792 | 0.01329542 | 0.001011724 | 0.15136 | -5.008005163 |
| GLOD4 | 38276 | ES | 0.000947302 | 0.009052547 | 0.010044516 | 0.04844 | -1.573299092 |
| SLC43A2 | 38350 | ES | 0.001639634 | 0.013500389 | 0.036647059 | 0.62956 | -2.843687988 |
| MINK1 | 38597 | ES | 0.003983693 | 0.025779966 | 0.054976623 | 0.68362 | -2.520494142 |
| CAMTA2 | 38633 | ES | 0.003000456 | 0.021116976 | 0.122555195 | 0.81736 | -1.897518136 |
| MYH10 | 39196 | ES | 7.88E-07 | 2.59E-05 | 0.0214125 | 0.63962 | -3.396899386 |
| MYH10 | 39199 | ES | 5.98E-05 | 0.001008656 | 0.181925424 | 0.91146 | -1.611450866 |
| AKAP10 | 39785 | ES | 0.004772012 | 0.029533825 | 0.019538571 | 0.44168 | -3.118195104 |
| IFT20 | 39882 | ES | 0.000190751 | 0.002576422 | 0.032592258 | 0.23832 | -1.989539531 |
| GIT1 | 40046 | ES | 0.005839131 | 0.033935014 | 0.141041935 | 0.79102 | -1.724265991 |
| DDX52 | 40542 | ES | 0.000147351 | 0.002101643 | 0.006157895 | 0.30478 | -3.901855249 |
| MRPL45 | 40572 | ES | 0.000670525 | 0.006937374 | 0.01559871 | 0.27976 | -2.886743895 |
| MED1 | 40648 | ES | 0.004555647 | 0.028511673 | 0.020736842 | 0.18218 | -2.173083279 |
| NKIRAS2 | 40978 | ES | 5.48E-05 | 0.000944706 | 0.002214839 | 0.3672 | -5.110727075 |
| ATP6V0A1 | 41046 | ES | 0.002208518 | 0.016870249 | 0.123154839 | 0.82982 | -1.907766395 |
| LSM12 | 41759 | ES | 0.000327548 | 0.003967796 | 0.068949032 | 0.43016 | -1.830789664 |
| FMNL1 | 41954 | ES | 0.000407819 | 0.00473416 | 0.126789542 | 0.7749 | -1.810205423 |
| SNX11 | 42174 | ES | 0.000293285 | 0.003613893 | 0.06858129 | 0.48866 | -1.963647189 |
| CALCOCO2 | 42231 | ES | 9.03E-05 | 0.001412113 | 0.081050968 | 0.42664 | -1.660862378 |
| SAMD14 | 42352 | ES | 0.009870562 | 0.048145411 | 0.002257419 | 0.0141 | -1.831952515 |
| SKA2 | 42731 | ES | 0.010269481 | 0.049465269 | 0.0087 | 0.15536 | -2.882421979 |
| SKA2 | 42734 | ES | 0.004517815 | 0.028369384 | 0.020168387 | 0.24806 | -2.509554269 |
| SKA2 | 42751 | ES | 0.001414642 | 0.012215 | 0.005477483 | 0.05022 | -2.215767584 |
| DDX5 | 43064 | ES | 0.000513242 | 0.005648287 | 0.081348344 | 0.5589 | -1.927230085 |
| AMZ2 | 43131 | ES | 0.001236226 | 0.011022645 | 0.01172 | 0.0987 | -2.130788162 |
| EXOC7 | 43564 | ES | 0.000286378 | 0.003549151 | 0.058783871 | 0.70778 | -2.488265797 |
| PGS1 | 43881 | ES | 0.000294889 | 0.003624753 | 0.058716556 | 0.43796 | -2.009405846 |
| CSNK1D | 44311 | ES | 0.002721762 | 0.019652374 | 0.042786452 | 0.37438 | -2.169049823 |
| DLGAP1 | 44525 | ES | 8.92E-08 | 3.62E-06 | 0.118773984 | 0.68298 | -1.749243186 |
| EPB41L3 | 44540 | ES | 0.002333636 | 0.017585648 | 0.066267586 | 0.442 | -1.897609 |
| EPB41L3 | 44541 | ES | 0.000890575 | 0.008654179 | 0.179670548 | 0.83066 | -1.531095681 |
| PTPRM | 44569 | ES | 0.000163539 | 0.002280593 | 0.099095161 | 0.67098 | -1.912658717 |
| SOGA2 | 44579 | ES | 0.002078072 | 0.016061523 | 0.125358197 | 0.79276 | -1.844345315 |
| TUBB6 | 44678 | ES | 0.008966462 | 0.045210777 | 0.024787097 | 0.38358 | -2.739224978 |
| FAM210A | 44743 | ES | 0.006260909 | 0.035448347 | 0.007408571 | 0.07598 | -2.327832517 |
| SS18 | 44966 | ES | 0.001858721 | 0.0147155 | 0.001822078 | 0.07608 | -3.731807853 |
| INO80C | 45189 | ES | 0.000332062 | 0.004011461 | 0.12125871 | 0.6928 | -1.742814998 |
| INO80C | 45190 | ES | 0.000892868 | 0.008670854 | 0.11992 | 0.71998 | -1.79239858 |
| TPGS2 | 45270 | ES | 0.009642185 | 0.047307758 | 0.02589708 | 0.3394 | -2.57304912 |
| HAUS1 | 45389 | ES | 7.06E-06 | 0.000169275 | 0.003620645 | 0.65288 | -5.194741114 |
| DYM | 45472 | ES | 0.003854909 | 0.025206979 | 0.016788816 | 0.32236 | -2.954955996 |
| WDR7 | 45631 | ES | 0.005356469 | 0.032106776 | 0.075358621 | 0.78814 | -2.347417412 |
| NEDD4L | 45654 | ES | 0.001260998 | 0.01117652 | 0.004060294 | 0.16438 | -3.700925407 |
| MBP | 45845 | ES | 0.001986158 | 0.015510607 | 1.43E-05 | 0.00058 | -3.703768067 |
| PQLC1 | 46254 | ES | 0.001627757 | 0.013439724 | 0.129416774 | 0.60062 | -1.534924451 |
| PQLC1 | 46262 | ES | 0.00912973 | 0.045672956 | 0.036850331 | 0.18396 | -1.607853738 |
| ZNF554 | 46626 | ES | 4.10E-08 | 1.78E-06 | 0.112558273 | 0.7234 | -1.860491246 |
| DPP9 | 46826 | ES | 0.000719629 | 0.007319476 | 0.009888435 | 0.2127 | -3.068516792 |
| PTPRS | 46845 | ES | 0.004762229 | 0.029485418 | 0.015198601 | 0.21952 | -2.670239934 |
| C19orf82 | 47381 | ES | 0.000258067 | 0.003262839 | 0.124567763 | 0.56482 | -1.511657246 |
| HOOK2 | 47866 | ES | 0.009122198 | 0.045665666 | 0.099095946 | 0.66608 | -1.905321252 |
| TECR | 48003 | ES | 0.009509355 | 0.046900961 | 0.001220645 | 0.01016 | -2.119078902 |
| ARMC6 | 48567 | ES | 0.002732284 | 0.019699963 | 0.004649286 | 0.29584 | -4.15310517 |
| ARMC6 | 48579 | ES | 0.000254653 | 0.003230543 | 0.00082549 | 0.02864 | -3.546582234 |
| LSM14A | 48955 | ES | 0.000245582 | 0.003139319 | 0.00104 | 0.09378 | -4.501730901 |
| GRAMD1A | 49012 | ES | 0.005601978 | 0.033135438 | 0.056043226 | 0.63584 | -2.428823677 |
| TBCB | 49356 | ES | 0.002736902 | 0.019720945 | 0.000976774 | 0.00676 | -1.934522666 |
| ZNF260 | 49390 | ES | 6.51E-06 | 0.000158365 | 0.050736752 | 0.70284 | -2.628478728 |
| SIRT2 | 49711 | ES | 0.001088859 | 0.010072824 | 0.010373548 | 0.24554 | -3.164200725 |
| SNRPA | 50001 | ES | 1.51E-05 | 0.000325581 | 0.003347742 | 0.04486 | -2.59526046 |
| APOC1 | 50359 | ES | 0.008728605 | 0.04437508 | 0.000488387 | 0.01814 | -3.614766401 |
| MARK4 | 50420 | ES | 6.68E-05 | 0.001095853 | 0.092439355 | 0.90062 | -2.276530609 |
| RUVBL2 | 50861 | ES | 0.000361846 | 0.004313743 | 0.013109032 | 0.19468 | -2.698055707 |
| RUVBL2 | 50865 | ES | 0.000841214 | 0.008297885 | 0.01790129 | 0.2962 | -2.806162107 |
| RUVBL2 | 50869 | ES | 6.13E-05 | 0.001027643 | 0.068950667 | 0.67572 | -2.282387516 |
| AP2A1 | 51066 | ES | 6.66E-06 | 0.000161387 | 0.158608387 | 0.8238 | -1.647489592 |
| ZNF329 | 52415 | ES | 0.0056533 | 0.033288701 | 0.057782171 | 0.43364 | -2.015534437 |
| LPIN1 | 52711 | ES | 0.006804465 | 0.037455704 | 0.025572932 | 0.3539 | -2.627479926 |
| SMC6 | 52732 | ES | 0.003854021 | 0.025206979 | 0.013333333 | 0.1329 | -2.2993298 |
| WDR35 | 52757 | ES | 0.006467341 | 0.036177158 | 0.035628972 | 0.536425 | -2.711767631 |
| DTNB | 52867 | ES | 0.004723298 | 0.029280553 | 0.132275325 | 0.7907 | -1.788033086 |
| NRBP1 | 53009 | ES | 0.000982198 | 0.009309828 | 0.011500645 | 0.21884 | -2.945937734 |
| ZNF512 | 53026 | ES | 0.007386061 | 0.039613224 | 0.040323438 | 0.18202 | -1.507183696 |
| LBH | 53112 | ES | 0.003223581 | 0.022184215 | 0.004637013 | 0.14806 | -3.463547191 |
| ATL2 | 53255 | ES | 0.002201297 | 0.016832196 | 0.031786029 | 0.51508 | -2.785295362 |
| MTA3 | 53371 | ES | 0.003281529 | 0.022453902 | 0.015546753 | 0.45154 | -3.36881214 |
| DYNC2LI1 | 53403 | ES | 0.000273741 | 0.003434667 | 0.001631169 | 0.3225 | -5.286806304 |
| MCFD2 | 53478 | ES | 0.00083143 | 0.008222953 | 0.057757143 | 0.57166 | -2.292297381 |
| COMMD1 | 53712 | ES | 1.38E-05 | 0.000304182 | 0.003535484 | 0.0546 | -2.737183713 |
| EHBP1 | 53719 | ES | 0.000990439 | 0.009382022 | 0.068801408 | 0.7942 | -2.446111102 |
| ACTR2 | 53799 | ES | 0.006522283 | 0.036381997 | 0.001333548 | 0.01472 | -2.401363764 |
| TIA1 | 53873 | ES | 0.000991216 | 0.009383467 | 0.048039355 | 0.32594 | -1.914692748 |
| DCTN1 | 54045 | ES | 0.005450654 | 0.032502848 | 0.076443871 | 0.77862 | -2.320966363 |
| DCTN1 | 54048 | ES | 4.44E-07 | 1.55E-05 | 0.083896053 | 0.93566 | -2.411673599 |
| SNRNP200 | 54521 | ES | 4.34E-05 | 0.000782611 | 0.030347097 | 0.33038 | -2.387542652 |
| LMAN2L | 54569 | ES | 0.002408515 | 0.017986692 | 0.094270968 | 0.58494 | -1.825336007 |
| INPP4A | 54631 | ES | 0.001675496 | 0.013688983 | 0.019184397 | 0.4293 | -3.108058674 |
| CLASP1 | 55173 | ES | 0.005412267 | 0.032312394 | 0.021413235 | 0.5323 | -3.213198038 |
| CLASP1 | 55176 | ES | 0.00641992 | 0.03602488 | 0.014016216 | 0.1502 | -2.371752779 |
| BIN1 | 55184 | ES | 0.000284181 | 0.003530656 | 0.079729032 | 0.36996 | -1.534761103 |
| SMPD4 | 55298 | ES | 0.008523836 | 0.043723054 | 0.047260526 | 0.43796 | -2.226452174 |
| SMPD4 | 55299 | ES | 0.003274858 | 0.022428386 | 0.070325828 | 0.49666 | -1.954766562 |
| MZT2B | 55331 | ES | 0.00428086 | 0.027291224 | 0.015503226 | 0.12768 | -2.108479015 |
| RAB3GAP1 | 55432 | ES | 0.003054259 | 0.021377141 | 0.033556129 | 0.52396 | -2.748195815 |
| MMADHC | 55561 | ES | 0.0023776 | 0.017800033 | 0.003734194 | 0.03422 | -2.215278391 |
| CERS6 | 55828 | ES | 0.00012169 | 0.00180411 | 0.122972185 | 0.86186 | -1.947134649 |
| SSB | 55885 | ES | 0.00078944 | 0.007906496 | 0.002723226 | 0.23322 | -4.450165078 |
| OSBPL6 | 56154 | ES | 0.00117823 | 0.010625278 | 0.082890345 | 0.71958 | -2.161149121 |
| NCKAP1 | 56467 | ES | 0.000243844 | 0.003122405 | 0.066168212 | 0.7597 | -2.440723453 |
| CFLAR | 56799 | ES | 0.000103712 | 0.001574845 | 0.013764052 | 0.59488 | -3.766299418 |
| ABI2 | 57019 | ES | 0.000404917 | 0.004711397 | 0.121125806 | 0.81436 | -1.9055728 |
| ABI2 | 57022 | ES | 0.005167259 | 0.031257095 | 0.041695484 | 0.71668 | -2.844236614 |
| MAP2 | 57228 | ES | 4.79E-08 | 2.03E-06 | 0.178668421 | 0.93584 | -1.65591283 |
| RPE | 57242 | ES | 0.000130767 | 0.001912272 | 0.042516129 | 0.24092 | -1.734581418 |
| RQCD1 | 57497 | ES | 7.10E-05 | 0.001153633 | 0.013256129 | 0.23058 | -2.85613786 |
| RQCD1 | 57499 | ES | 0.000563873 | 0.006063452 | 0.020025974 | 0.14134 | -1.954138203 |
| ATG16L1 | 58036 | ES | 0.002466256 | 0.018281667 | 0.039531579 | 0.54112 | -2.616541246 |
| ATG16L1 | 58037 | ES | 8.64E-07 | 2.82E-05 | 0.050947748 | 0.93024 | -2.904642064 |
| LRRFIP1 | 58132 | ES | 0.000235576 | 0.003037238 | 0.069181935 | 0.78794 | -2.432682164 |
| KIF1A | 58302 | ES | 0.00826905 | 0.042840094 | 0.117943066 | 0.55836 | -1.554801902 |
| HM13 | 58890 | ES | 0.000442447 | 0.005019851 | 0.052765806 | 0.60002 | -2.431099612 |
| HM13 | 58891 | ES | 0.000732547 | 0.007405774 | 0.027918065 | 0.46738 | -2.817868678 |
| ERGIC3 | 59176 | ES | 0.004798859 | 0.029643915 | 0.044551613 | 0.609 | -2.61516991 |
| NFS1 | 59219 | ES | 0.000152245 | 0.002155078 | 0.038291447 | 0.27576 | -1.974304357 |
| RBM39 | 59237 | ES | 0.001517247 | 0.012835547 | 0.002584516 | 0.01278 | -1.598343143 |
| RPN2 | 59332 | ES | 1.47E-05 | 0.000319012 | 0.096624516 | 0.7053 | -1.987790745 |
| SRC | 59350 | ES | 0.000266276 | 0.003358156 | 0.045763265 | 0.65042 | -2.654136606 |
| ARFGAP1 | 60112 | ES | 0.003508747 | 0.023495319 | 0.03232129 | 0.76048 | -3.158223657 |
| TPD52L2 | 60170 | ES | 0.005411486 | 0.032312394 | 0.033207742 | 0.68262 | -3.023155296 |
| TPD52L2 | 60171 | ES | 0.007471605 | 0.039872433 | 0.037898065 | 0.33694 | -2.18500483 |
| USP16 | 60303 | ES | 3.47E-05 | 0.000654123 | 0.089699351 | 0.78936 | -2.174758961 |
| GRIK1 | 60327 | ES | 5.19E-05 | 0.000901977 | 0.034455469 | 0.37568 | -2.389069988 |
| SYNJ1 | 60370 | ES | 0.000205309 | 0.002731331 | 0.061969725 | 0.82746 | -2.591714812 |
| SYNJ1 | 60371 | ES | 1.72E-06 | 5.04E-05 | 0.113536607 | 0.91922 | -2.09140017 |
| ITSN1 | 60475 | ES | 0.003730971 | 0.024599865 | 0.015733333 | 0.428425 | -3.30433409 |
| ARVCF | 61109 | ES | 0.005946996 | 0.034335918 | 0.063337908 | 0.56596 | -2.190039384 |
| ZNF74 | 61152 | ES | 0.006461267 | 0.036162409 | 0.078363889 | 0.58876 | -2.01665541 |
| AP1B1 | 61605 | ES | 6.05E-08 | 2.54E-06 | 0.152323226 | 0.80906 | -1.669868331 |
| RBFOX2 | 61983 | ES | 0.002907239 | 0.020654312 | 0.027875839 | 0.34866 | -2.52633691 |
| TXN2 | 62050 | ES | 0.001081924 | 0.010036258 | 0.003416774 | 0.02386 | -1.943506529 |
| MPST | 62073 | ES | 0.001795963 | 0.014345759 | 0.020576129 | 0.21902 | -2.365031431 |
| SH3BP1 | 62140 | ES | 0.000589137 | 0.00628945 | 0.005811842 | 0.54696 | -4.544478095 |
| TBC1D22A | 62731 | ES | 8.90E-05 | 0.001395642 | 0.002835333 | 0.09154 | -3.474616527 |
| SHANK3 | 62904 | ES | 0.002650574 | 0.01921219 | 0.179817829 | 0.82924 | -1.528565339 |
| TRNT1 | 62979 | ES | 5.21E-05 | 0.000901977 | 0.026408609 | 0.43858 | -2.80985217 |
| SH3BP5 | 63567 | ES | 0.002633211 | 0.019126617 | 0.004092617 | 0.05152 | -2.532785353 |
| LRRFIP2 | 63958 | ES | 7.81E-05 | 0.001249967 | 0.084245455 | 0.69024 | -2.103304747 |
| CTNNB1 | 64254 | ES | 0.002181794 | 0.016717082 | 0.027814194 | 0.14886 | -1.677459817 |
| POMGNT2 | 64340 | ES | 0.000241995 | 0.003101367 | 0.101333775 | 0.52366 | -1.642422849 |
| TCAIM | 64361 | ES | 5.18E-06 | 0.000129331 | 0.008856376 | 0.47936 | -3.991314247 |
| MAP4 | 64567 | ES | 0.001713123 | 0.013920746 | 0.049003226 | 0.2313 | -1.551829441 |
| PBRM1 | 65243 | ES | 0.006133677 | 0.034940344 | 0.095818018 | 0.570066667 | -1.783302566 |
| CADPS | 65501 | ES | 3.22E-11 | 2.32E-09 | 0.129759124 | 0.58204 | -1.500859334 |
| MAGI1 | 65531 | ES | 2.77E-16 | 3.36E-14 | 0.146131343 | 0.93 | -1.850678757 |
| ARL6IP5 | 65577 | ES | 0.000513833 | 0.005650659 | 0.002446452 | 0.0314 | -2.552169239 |
| CLDND1 | 65782 | ES | 0.000389612 | 0.004568667 | 0.029269032 | 0.16646 | -1.738225003 |
| CD47 | 66013 | ES | 0.010190067 | 0.049208889 | 0.082049677 | 0.67226 | -2.103320284 |
| RAB43 | 66695 | ES | 0.006612779 | 0.036709714 | 0.008543046 | 0.09876 | -2.447575004 |
| ISY1 | 66700 | ES | 0.001031483 | 0.009684197 | 0.011505161 | 0.22448 | -2.970990874 |
| ATP1B3 | 67081 | ES | 0.000230046 | 0.002982554 | 0.001899355 | 0.06526 | -3.536865021 |
| ATP1B3 | 67084 | ES | 0.001102142 | 0.010147848 | 0.036836774 | 0.52098 | -2.649215008 |
| GYG1 | 67204 | ES | 0.005071692 | 0.030817601 | 0.028052903 | 0.48752 | -2.855239187 |
| EIF2A | 67287 | ES | 0.001154181 | 0.010465517 | 0.002595484 | 0.3359 | -4.863040535 |
| MFSD1 | 67452 | ES | 0.001832162 | 0.014583519 | 0.026541935 | 0.11952 | -1.504757768 |
| NLGN1 | 67666 | ES | 0.000232301 | 0.003002734 | 0.022496639 | 0.145025 | -1.863540236 |
| EIF4G1 | 67884 | ES | 0.003433783 | 0.023158393 | 0.029746 | 0.66458 | -3.106460593 |
| VPS8 | 67974 | ES | 0.003372857 | 0.022948334 | 0.048457724 | 0.7115 | -2.686683679 |
| VPS8 | 67977 | ES | 0.000880098 | 0.008563436 | 0.047664407 | 0.40354 | -2.136090685 |
| APOD | 68181 | ES | 0.00515836 | 0.031231454 | 0.009136774 | 0.12352 | -2.604095696 |
| TCTEX1D2 | 68236 | ES | 0.00752102 | 0.040022476 | 0.023548387 | 0.16572 | -1.951242288 |
| FAM193A | 68579 | ES | 0.002469366 | 0.018286689 | 0.011142581 | 0.14594 | -2.572421715 |
| AFAP1 | 68741 | ES | 0.001921228 | 0.015121369 | 0.149286822 | 0.80226 | -1.681563311 |
| PACRGL | 68881 | ES | 0.001261716 | 0.01117652 | 0.120072932 | 0.78542 | -1.878119279 |
| PACRGL | 68882 | ES | 0.001022569 | 0.009623326 | 0.038904575 | 0.52146 | -2.595520713 |
| PACRGL | 68884 | ES | 0.001858893 | 0.0147155 | 0.018709804 | 0.33126 | -2.873855905 |
| STIM2 | 68991 | ES | 0.003951383 | 0.025603937 | 0.01355871 | 0.19574 | -2.669758127 |
| LIMCH1 | 69115 | ES | 0.009112874 | 0.045649393 | 0.082031613 | 0.65596 | -2.078995115 |
| OCIAD1 | 69234 | ES | 0.003028832 | 0.021251817 | 0.001134839 | 0.0367 | -3.47628622 |
| LPHN3 | 69401 | ES | 0.000577855 | 0.006196077 | 0.030096711 | 0.42732 | -2.653117266 |
| OSTC | 70305 | ES | 6.28E-05 | 0.001045797 | 0.007506452 | 0.19494 | -3.256928953 |
| ANK2 | 70397 | ES | 0.008667753 | 0.044155315 | 0.052954839 | 0.40204 | -2.027112136 |
| ANK2 | 70399 | ES | 0.007213978 | 0.038995637 | 0.056272483 | 0.64222 | -2.434725261 |
| CAMK2D | 70414 | ES | 3.67E-05 | 0.00068199 | 0.039880645 | 0.92094 | -3.139503765 |
| SEC24D | 70445 | ES | 0.006219069 | 0.035306369 | 0.01045871 | 0.15532 | -2.698052412 |
| ABCE1 | 70753 | ES | 3.75E-05 | 0.000692743 | 0.011957419 | 0.32908 | -3.31494893 |
| DCLK2 | 70809 | ES | 0.006701435 | 0.037037748 | 0.020377778 | 0.54916 | -3.293944856 |
| MND1 | 70870 | ES | 0.002550824 | 0.018742247 | 0.00619931 | 0.318375 | -3.938791882 |
| DCTD | 71247 | ES | 0.009951836 | 0.048400501 | 0.065295918 | 0.34602 | -1.667567048 |
| SORBS2 | 71376 | ES | 2.18E-18 | 3.25E-16 | 0.156845255 | 0.9647 | -1.816557487 |
| FBXO4 | 71889 | ES | 0.000238072 | 0.003061532 | 0.139767097 | 0.63928 | -1.5203651 |
| KIF2A | 72178 | ES | 0.001884527 | 0.01488913 | 0.151349351 | 0.76262 | -1.617169128 |
| MEF2C | 72754 | ES | 8.49E-07 | 2.77E-05 | 0.093733766 | 0.92014 | -2.284067343 |
| AP3S1 | 73040 | ES | 0.002778897 | 0.019959465 | 0.02394 | 0.30882 | -2.557207883 |
| HSD17B4 | 73082 | ES | 0.002628659 | 0.01911787 | 0.004759355 | 0.08724 | -2.90855082 |
| SAR1B | 73418 | ES | 0.001057647 | 0.009865521 | 0.001118065 | 0.267 | -5.475649579 |
| APBB3 | 73678 | ES | 0.006101952 | 0.034828521 | 0.065443269 | 0.60224 | -2.219472388 |
| APBB3 | 73682 | ES | 0.002799254 | 0.020086482 | 0.146378302 | 0.82148 | -1.724913212 |
| IK | 73709 | ES | 0.000401727 | 0.004688786 | 0.005758065 | 0.15066 | -3.264424246 |
| ABLIM3 | 74024 | ES | 5.36E-12 | 4.22E-10 | 0.125650714 | 0.8082 | -1.861303604 |
| CYFIP2 | 74336 | ES | 0.004448104 | 0.028001818 | 0.010257616 | 0.106 | -2.33541865 |
| CPEB4 | 74591 | ES | 0.006342 | 0.035667523 | 0.090295192 | 0.6828 | -2.023117773 |
| CPEB4 | 74594 | ES | 0.00148823 | 0.012662646 | 0.092662609 | 0.66722 | -1.974154794 |
| CPEB4 | 74595 | ES | 0.002822076 | 0.020161671 | 0.0507375 | 0.57684 | -2.43089965 |
| CLTB | 74653 | ES | 0.000602676 | 0.006398325 | 0.086754839 | 0.82402 | -2.251108607 |
| DBN1 | 74775 | ES | 0.000422246 | 0.004841823 | 0.044852903 | 0.71568 | -2.769844821 |
| ZNF354A | 74880 | ES | 0.00021696 | 0.002848529 | 0.079523776 | 0.60978 | -2.037042187 |
| TMEM14B | 75315 | ES | 6.70E-05 | 0.001099163 | 0.112418919 | 0.7438 | -1.88953994 |
| MCUR1 | 75402 | ES | 0.007611179 | 0.040387878 | 0.001907742 | 0.05708 | -3.398533483 |
| TAF8 | 76168 | ES | 0.001369763 | 0.011909566 | 0.072092857 | 0.40398 | -1.723410401 |
| TJAP1 | 76274 | ES | 0.000528425 | 0.005773116 | 0.152301935 | 0.73268 | -1.570844076 |
| DST | 76561 | ES | 1.11E-05 | 0.00025291 | 0.121343226 | 0.7206 | -1.781461091 |
| SMIM8 | 76952 | ES | 0.004293947 | 0.027340494 | 0.036811034 | 0.53808 | -2.682209598 |
| GOPC | 77347 | ES | 0.003131245 | 0.021760956 | 0.097464885 | 0.7813 | -2.081467035 |
| TPD52L1 | 77415 | ES | 0.002047978 | 0.015912934 | 0.119146763 | 0.68046 | -1.742413008 |
| TPD52L1 | 77418 | ES | 0.005708251 | 0.033502301 | 0.089587591 | 0.53798 | -1.792604565 |
| MAP3K4 | 78359 | ES | 0.005764138 | 0.033716843 | 0.018015517 | 0.14744 | -2.102187858 |
| MLLT4 | 78450 | ES | 0.000683478 | 0.007027858 | 0.102482301 | 0.74088 | -1.978148559 |
| MLLT4 | 78453 | ES | 0.002211129 | 0.016881615 | 0.038746479 | 0.58024 | -2.706401927 |
| MLLT4 | 78457 | ES | 0.006000851 | 0.034543402 | 0.1307 | 0.6802 | -1.649482252 |
| ICA1 | 78789 | ES | 0.005631822 | 0.033206298 | 0.027958333 | 0.13502 | -1.574707609 |
| FAM221A | 78990 | ES | 0.000120041 | 0.001783176 | 0.107614194 | 0.58034 | -1.685061589 |
| ADCYAP1R1 | 79172 | ES | 3.77E-06 | 9.81E-05 | 0.068367333 | 0.43618 | -1.853159874 |
| CRCP | 79873 | ES | 0.000511592 | 0.005638389 | 0.068332903 | 0.39166 | -1.74600272 |
| ELN | 80056 | ES | 7.42E-05 | 0.001202765 | 0.1602 | 0.89762 | -1.723323782 |
| ADAM22 | 80353 | ES | 1.54E-05 | 0.000332038 | 0.057660417 | 0.66518 | -2.445486762 |
| TRRAP | 80598 | ES | 0.005981452 | 0.034483479 | 0.093118519 | 0.48066 | -1.641287085 |
| SMURF1 | 80600 | ES | 0.000417638 | 0.004803721 | 0.044814286 | 0.32882 | -1.992983522 |
| ARPC1B | 80609 | ES | 0.000175769 | 0.00241309 | 0.003145806 | 0.1876 | -4.088241756 |
| BUD31 | 80622 | ES | 0.000135457 | 0.001965565 | 0.019451613 | 0.16256 | -2.123117172 |
| ZKSCAN1 | 80872 | ES | 0.001601993 | 0.013298046 | 0.034275 | 0.40788 | -2.476556788 |
| LHFPL3 | 81264 | ES | 0.006607641 | 0.036708303 | 0.023910145 | 0.16912 | -1.956305679 |
| PUS7 | 81290 | ES | 0.001459528 | 0.012506296 | 0.002458442 | 0.17358 | -4.257110952 |
| NRCAM | 81400 | ES | 0.001600735 | 0.01329542 | 0.008918182 | 0.47754 | -3.980555832 |
| NRCAM | 81401 | ES | 0.001702319 | 0.013847923 | 0.024664935 | 0.614 | -3.214612326 |
| NRCAM | 81402 | ES | 0.000115261 | 0.001722383 | 0.076048701 | 0.78904 | -2.339443075 |
| NRCAM | 81405 | ES | 0.000646472 | 0.006767699 | 0.078915436 | 0.77752 | -2.287732515 |
| DOCK4 | 81438 | ES | 0.006836929 | 0.037593105 | 0.039760769 | 0.58326 | -2.685752328 |
| CADPS2 | 81609 | ES | 1.31E-07 | 5.15E-06 | 0.102905344 | 0.75314 | -1.990441563 |
| CADPS2 | 81610 | ES | 3.63E-07 | 1.30E-05 | 0.08182439 | 0.913725 | -2.412954283 |
| CADPS2 | 81612 | ES | 8.89E-05 | 0.001394592 | 0.074776667 | 0.899275 | -2.48708299 |
| IRF5 | 81735 | ES | 0.003906091 | 0.025431036 | 0.021583916 | 0.39068 | -2.895940399 |
| CALD1 | 81859 | ES | 0.001749249 | 0.0141227 | 0.035567097 | 0.47282 | -2.587293804 |
| C7orf55-LUC7L2 | 81960 | ES | 0.001336184 | 0.011664836 | 0.013577124 | 0.1814 | -2.592318207 |
| C7orf55-LUC7L2 | 81961 | ES | 0.009293237 | 0.046036013 | 0.034558824 | 0.21366 | -1.821723065 |
| TBXAS1 | 81971 | ES | 0.002811955 | 0.020119997 | 0.007121333 | 0.20562 | -3.362934831 |
| ABCB8 | 82313 | ES | 0.010074849 | 0.048872282 | 0.026070588 | 0.1332 | -1.631043967 |
| FBXO25 | 82547 | ES | 0.000164775 | 0.0022957 | 0.10345974 | 0.74182 | -1.969924072 |
| FBXO16 | 83217 | ES | 5.22E-07 | 1.80E-05 | 0.126496063 | 0.8984 | -1.960404218 |
| TMEM66 | 83267 | ES | 0.001120801 | 0.010261267 | 0.008634416 | 0.04494 | -1.649572237 |
| DCTN6 | 83284 | ES | 3.88E-05 | 0.000713788 | 0.020448387 | 0.23202 | -2.428919566 |
| FGFR1 | 83429 | ES | 0.006494716 | 0.036295566 | 0.021893289 | 0.210125 | -2.261522459 |
| TACC1 | 83447 | ES | 0.00603463 | 0.034678361 | 0.018720155 | 0.36956 | -2.982712356 |
| VDAC3 | 83725 | ES | 0.004231871 | 0.027082897 | 0.092318065 | 0.7523 | -2.097895343 |
| HGSNAT | 83764 | ES | 0.001236068 | 0.011022645 | 0.016780667 | 0.10558 | -1.839241529 |
| TCEA1 | 83855 | ES | 0.008134291 | 0.042400412 | 0.008319355 | 0.09002 | -2.381447159 |
| NSMAF | 83944 | ES | 0.007372871 | 0.039584415 | 0.091108966 | 0.43038 | -1.552612326 |
| TPD52 | 84276 | ES | 0.007431333 | 0.039742168 | 0.071968387 | 0.78974 | -2.395476823 |
| CA2 | 84357 | ES | 0.004682905 | 0.029117257 | 0.002426623 | 0.01674 | -1.931300331 |
| RMDN1 | 84377 | ES | 0.000479748 | 0.005354154 | 0.032453896 | 0.22954 | -1.956256805 |
| FAM92A1 | 84528 | ES | 0.00022933 | 0.002977126 | 0.001894194 | 0.02714 | -2.662215475 |
| RRM2B | 84774 | ES | 0.002421475 | 0.018038665 | 0.017415108 | 0.54574 | -3.444804571 |
| MTSS1 | 85114 | ES | 0.00647905 | 0.036221477 | 0.149248182 | 0.671225 | -1.503493831 |
| PTK2 | 85315 | ES | 0.007620432 | 0.040408449 | 0.076732258 | 0.65196 | -2.139661016 |
| PTK2 | 85316 | ES | 0.002648477 | 0.019206249 | 0.091529412 | 0.62948 | -1.928233721 |
| UNC13B | 86247 | ES | 0.002623399 | 0.019088846 | 0.034951974 | 0.619 | -2.874130334 |
| CLTA | 86331 | ES | 0.010326591 | 0.049660794 | 0.013849677 | 0.51178 | -3.609632904 |
| PRUNE2 | 86643 | ES | 0.001424701 | 0.012273654 | 0.08059 | 0.855 | -2.361726897 |
| TMEM245 | 87161 | ES | 0.000821633 | 0.008138758 | 0.052927559 | 0.8042 | -2.720923826 |
| AKAP2 | 87178 | ES | 0.00440512 | 0.027795115 | 0.0177475 | 0.56512 | -3.460793437 |
| AKAP2 | 87179 | ES | 0.004402136 | 0.027793916 | 0.027716667 | 0.68046 | -3.200735123 |
| VAV2 | 88085 | ES | 0.00534602 | 0.032095333 | 0.027557554 | 0.51064 | -2.919388152 |
| NSMF | 88323 | ES | 1.49E-06 | 4.45E-05 | 0.159234641 | 0.86462 | -1.691911263 |
| NSMF | 88325 | ES | 1.48E-06 | 4.44E-05 | 0.114706452 | 0.87976 | -2.037272873 |
| ARHGAP6 | 88480 | ES | 2.04E-05 | 0.000417948 | 0.102948624 | 0.76888 | -2.010704844 |
| SH3KBP1 | 88646 | ES | 0.001294257 | 0.011384767 | 0.012542208 | 0.57766 | -3.829885881 |
| CXorf23 | 88651 | ES | 0.000788269 | 0.007900034 | 0.046565414 | 0.28015 | -1.794467107 |
| CASK | 88869 | ES | 0.00991243 | 0.048271319 | 0.025662338 | 0.53422 | -3.035783283 |
| MAOB | 88877 | ES | 0.008216075 | 0.042682906 | 0.004299333 | 0.05982 | -2.632880081 |
| KDM5C | 89196 | ES | 0.001278342 | 0.01127777 | 0.000761074 | 0.09904 | -4.868548724 |
| IQSEC2 | 89211 | ES | 2.84E-08 | 1.25E-06 | 0.184438583 | 0.93528 | -1.623529427 |
| ZC4H2 | 89319 | ES | 0.007754682 | 0.040918266 | 0.002151389 | 0.0493 | -3.131810453 |
| GDPD2 | 89374 | ES | 0.000715899 | 0.007291404 | 0.02904898 | 0.41864 | -2.668028005 |
| CSTF2 | 89612 | ES | 0.002805334 | 0.020110914 | 0.042384314 | 0.63078 | -2.700178814 |
| TMEM255A | 89994 | ES | 0.001824052 | 0.014539224 | 0.165154902 | 0.79752 | -1.574623079 |
| KIF21A | 93028 | ES | 0.000221698 | 0.00290312 | 0.121221854 | 0.83224 | -1.926498487 |
| TXN2 | 96153 | ES | 0.000606418 | 0.006424446 | 0.054349669 | 0.2663 | -1.589184972 |
| SIRT2 | 100925 | ES | 0.005947631 | 0.034335918 | 0.048541071 | 0.62726 | -2.558950855 |
| SIRT2 | 100926 | ES | 2.78E-05 | 0.000545489 | 0.164884167 | 0.83854 | -1.626419077 |
| ANK2 | 105815 | ES | 9.66E-08 | 3.88E-06 | 0.131287273 | 0.84152 | -1.857821937 |
| NRCAM | 136557 | ES | 0.005709632 | 0.033502301 | 0.008035514 | 0.18682 | -3.146274617 |
| NPIPA8 | 266561 | ES | 0.009159468 | 0.04573752 | 0.132914 | 0.70284 | -1.665426967 |
| NRXN2 | 271646 | ES | 0.000151081 | 0.002142643 | 0.010642308 | 0.11338 | -2.36590766 |
| NDUFA13 | 302227 | ES | 0.000381644 | 0.004492765 | 0.059422581 | 0.45342 | -2.032144549 |
| UBXN11 | 1261 | ES | 1.27E-13 | 1.20E-11 | 0.364968841 | 0.04468 | 2.100286007 |
| UBXN11 | 1262 | ES | 7.76E-12 | 6.02E-10 | 0.708468212 | 0.09702 | 1.988188049 |
| PUM1 | 1453 | ES | 0.000231629 | 0.002999213 | 0.009886207 | 0.00128 | 2.044280465 |
| ST3GAL3 | 2475 | ES | 9.17E-05 | 0.00142846 | 0.010772115 | 0.001375 | 2.058507155 |
| ST3GAL3 | 2476 | ES | 6.09E-07 | 2.06E-05 | 0.063704274 | 0.010025 | 1.849169675 |
| ST3GAL3 | 2477 | ES | 0.000299006 | 0.003666354 | 0.009919048 | 0.0014 | 1.957984674 |
| ST3GAL3 | 2478 | ES | 4.98E-05 | 0.00087306 | 0.048450893 | 0.010125 | 1.565543154 |
| ECHDC2 | 3037 | ES | 6.29E-05 | 0.001046389 | 0.289622481 | 0.039925 | 1.98157559 |
| SSBP3 | 3144 | ES | 3.39E-47 | 6.37E-44 | 0.288089677 | 0.02672 | 2.377859463 |
| LRIF1 | 4130 | ES | 2.72E-06 | 7.50E-05 | 0.054546853 | 0.00496 | 2.397654282 |
| IGSF3 | 4366 | ES | 8.93E-28 | 2.80E-25 | 0.363773077 | 0.007575 | 3.871676907 |
| SUSD4 | 9916 | ES | 1.48E-06 | 4.44E-05 | 0.022315972 | 0.0046 | 1.579246362 |
| MRPL55 | 10109 | ES | 1.41E-16 | 1.75E-14 | 0.045592903 | 0.00626 | 1.985571888 |
| MRPL55 | 10125 | ES | 8.30E-18 | 1.13E-15 | 0.013033548 | 0.00166 | 2.060709076 |
| MRPL55 | 10133 | ES | 1.10E-11 | 8.18E-10 | 0.136545033 | 0.01708 | 2.078746285 |
| MRPL55 | 10134 | ES | 6.08E-14 | 5.86E-12 | 0.062144595 | 0.00652 | 2.254589465 |
| MRPL55 | 10154 | ES | 1.80E-05 | 0.000376165 | 0.1505 | 0.03312 | 1.513825755 |
| MRPL55 | 10162 | ES | 4.70E-18 | 6.73E-16 | 0.040744218 | 0.00436 | 2.234841875 |
| MRPL55 | 10163 | ES | 1.65E-15 | 1.88E-13 | 0.015748252 | 0.0017 | 2.226101108 |
| OPTN | 10782 | ES | 4.36E-08 | 1.88E-06 | 0.029358824 | 0.00348 | 2.132560839 |
| NEBL | 10964 | ES | 8.56E-05 | 0.001347028 | 0.003935938 | 3.00E-04 | 2.574121904 |
| KIAA1217 | 11004 | ES | 1.27E-06 | 3.90E-05 | 0.846316883 | 0.08058 | 2.351643376 |
| CREM | 11243 | ES | 0.000209305 | 0.002774675 | 0.154322759 | 0.019425 | 2.072485348 |
| CREM | 11250 | ES | 4.33E-06 | 0.000111019 | 0.071143796 | 0.005775 | 2.511164863 |
| CREM | 11255 | ES | 8.18E-06 | 0.000193658 | 0.071533333 | 0.0073 | 2.282289193 |
| CREM | 11258 | ES | 5.73E-23 | 1.23E-20 | 0.128838211 | 6.00E-04 | 5.369383066 |
| CREM | 11273 | ES | 0.00372791 | 0.024590466 | 0.063656481 | 0.01136 | 1.723402737 |
| CREM | 11279 | ES | 5.89E-20 | 9.95E-18 | 0.118536441 | 0.00036 | 5.796871677 |
| PPP3CB | 12157 | ES | 0.007028345 | 0.038281563 | 0.001962092 | 0.00032 | 1.813445281 |
| FRA10AC1 | 12564 | ES | 0.000896604 | 0.008684698 | 0.008306623 | 0.00122 | 1.918202231 |
| SORBS1 | 12618 | ES | 0.000276094 | 0.003450143 | 0.013456863 | 0.00232 | 1.757922032 |
| ABLIM1 | 13212 | ES | 0.00486624 | 0.029907682 | 0.02122197 | 0.00464 | 1.520322585 |
| C11orf49 | 15636 | ES | 8.46E-05 | 0.001334568 | 0.003531613 | 0.00042 | 2.129255247 |
| BSCL2 | 16411 | ES | 3.74E-06 | 9.76E-05 | 0.003471895 | 0.00026 | 2.591774325 |
| CNIH2 | 17002 | ES | 1.56E-27 | 4.69E-25 | 0.086664935 | 0.01812 | 1.565033062 |
| INTS4 | 17998 | ES | 9.71E-06 | 0.000224337 | 0.084041667 | 0.01124 | 2.011833863 |
| ELMOD1 | 18580 | ES | 1.24E-28 | 4.25E-26 | 0.142943846 | 0.02182 | 1.879624888 |
| C11orf57 | 18729 | ES | 0.001045254 | 0.009786708 | 0.095961905 | 0.0192 | 1.609041008 |
| FXYD6 | 18943 | ES | 9.20E-07 | 2.98E-05 | 0.003402581 | 0.00042 | 2.092034725 |
| FXYD6 | 18945 | ES | 1.38E-06 | 4.20E-05 | 0.007619355 | 0.00092 | 2.114073308 |
| GABARAPL1 | 20394 | ES | 0.000338634 | 0.004082351 | 0.00096 | 2.00E-05 | 3.871201011 |
| GABARAPL1 | 20395 | ES | 2.68E-06 | 7.42E-05 | 0.003195484 | 0.00014 | 3.127851379 |
| GABARAPL1 | 20400 | ES | 0.000604306 | 0.006406578 | 0.000269677 | 2.00E-05 | 2.601494228 |
| GABARAPL1 | 20401 | ES | 2.16E-07 | 8.06E-06 | 0.001865806 | 0.00014 | 2.58980623 |
| GABARAPL1 | 20402 | ES | 1.28E-09 | 7.24E-08 | 0.001491613 | 8.00E-05 | 2.925586664 |
| GABARAPL1 | 20409 | ES | 0.001592582 | 0.013278582 | 0.000212903 | 2.00E-05 | 2.36510545 |
| GABARAPL1 | 20410 | ES | 0.000443588 | 0.005025214 | 0.000921935 | 6.00E-05 | 2.732130685 |
| CCDC91 | 20920 | ES | 5.59E-13 | 4.89E-11 | 0.152408696 | 0.03286 | 1.534309587 |
| YAF2 | 21138 | ES | 0.005486426 | 0.032664335 | 0.001317647 | 0.00022 | 1.789975347 |
| CACNB3 | 21480 | ES | 3.36E-05 | 0.000637456 | 0.132643802 | 0.02372 | 1.721348778 |
| TMBIM4 | 22909 | ES | 0.002069895 | 0.016033081 | 0.008156774 | 0.00122 | 1.899997913 |
| GLTP | 24362 | ES | 0.005878347 | 0.034053635 | 0.002815584 | 0.00052 | 1.689096315 |
| VPS29 | 24440 | ES | 2.02E-19 | 3.20E-17 | 0.003113636 | 0.00014 | 3.101904148 |
| VPS29 | 24442 | ES | 2.64E-19 | 4.13E-17 | 0.011425325 | 0.00024 | 3.86294871 |
| VPS29 | 24443 | ES | 1.33E-05 | 0.000294667 | 0.002133117 | 0.00044 | 1.578564788 |
| VPS29 | 24445 | ES | 2.61E-08 | 1.18E-06 | 0.012751299 | 0.00184 | 1.935867554 |
| VPS29 | 24446 | ES | 4.37E-17 | 5.71E-15 | 0.315673548 | 0.06728 | 1.545845589 |
| TCTN1 | 24482 | ES | 0.007704233 | 0.040709219 | 0.015776471 | 0.00294 | 1.680110045 |
| PITPNM2 | 25044 | ES | 7.08E-18 | 9.85E-16 | 0.2426432 | 0.040675 | 1.785978398 |
| SERP2 | 25780 | ES | 2.32E-09 | 1.25E-07 | 0.079694631 | 0.01726 | 1.529810531 |
| SERP2 | 25781 | ES | 4.40E-05 | 0.000788833 | 0.680705161 | 0.1387 | 1.590815935 |
| THTPA | 26764 | ES | 0.001318926 | 0.011540983 | 0.046708497 | 0.0086 | 1.692163888 |
| THTPA | 26766 | ES | 4.42E-05 | 0.000789629 | 0.030663399 | 0.00656 | 1.542079115 |
| RTN1 | 27758 | ES | 2.56E-08 | 1.16E-06 | 0.020033913 | 0.00328 | 1.809583067 |
| ACYP1 | 28478 | ES | 3.69E-24 | 8.95E-22 | 0.113368387 | 0.01616 | 1.948103526 |
| IFI27L1 | 29071 | ES | 2.31E-05 | 0.000464013 | 0.010449032 | 0.00102 | 2.32670674 |
| SERPINA1 | 29132 | ES | 0.00845443 | 0.043496048 | 0.03894898 | 0.005566667 | 1.945456143 |
| WDR20 | 29347 | ES | 4.01E-06 | 0.000103691 | 0.063071094 | 0.00762 | 2.113486193 |
| C14orf2 | 29536 | ES | 8.93E-08 | 3.62E-06 | 0.010591613 | 0.00228 | 1.535887009 |
| MYO5A | 30660 | ES | 1.01E-05 | 0.000230828 | 0.449272028 | 0.05134 | 2.169158382 |
| SLTM | 30921 | ES | 0.007109711 | 0.038557103 | 0.003871429 | 0.00072 | 1.682127646 |
| EDC3 | 31733 | ES | 3.54E-05 | 0.00066604 | 0.041729801 | 0.00812 | 1.636885379 |
| AP3S2 | 32455 | ES | 9.72E-07 | 3.12E-05 | 0.087774194 | 0.01926 | 1.516737128 |
| HN1L | 33123 | ES | 0.001993489 | 0.015559772 | 0.031493548 | 0.004075 | 2.044911965 |
| LITAF | 34020 | ES | 2.31E-05 | 0.000464013 | 0.013016774 | 0.0018 | 1.978452183 |
| C16orf45 | 34160 | ES | 3.43E-24 | 8.45E-22 | 0.006613548 | 0.00072 | 2.217624398 |
| LYRM1 | 34412 | ES | 0.00062914 | 0.006632444 | 0.077275 | 0.016075 | 1.570105218 |
| LYRM1 | 34416 | ES | 5.21E-05 | 0.000901977 | 0.025772667 | 0.00416 | 1.823799425 |
| INO80E | 36011 | ES | 7.39E-07 | 2.45E-05 | 0.01105 | 0.00068 | 2.788092909 |
| CPNE2 | 36524 | ES | 0.000358556 | 0.004277909 | 0.007072109 | 9.00E-04 | 2.061519232 |
| VPS53 | 38222 | ES | 1.75E-05 | 0.000368606 | 0.040687591 | 0.00628 | 1.868553182 |
| TOM1L2 | 39511 | ES | 6.55E-05 | 0.001079679 | 0.901165068 | 0.12224 | 1.997702121 |
| FLOT2 | 40004 | ES | 0.002494115 | 0.018415532 | 0.333346715 | 0.06542 | 1.628355113 |
| SMARCE1 | 40873 | ES | 4.36E-16 | 5.24E-14 | 0.109029268 | 0.006025 | 2.895698883 |
| MAPT | 41997 | ES | 5.74E-33 | 2.54E-30 | 0.290194 | 0.03874 | 2.013677007 |
| MAPT | 42008 | ES | 2.39E-12 | 1.92E-10 | 0.072567797 | 0.01034 | 1.948501381 |
| BCAS3 | 42880 | ES | 4.54E-07 | 1.58E-05 | 0.060902128 | 0.0099 | 1.816733354 |
| PRKAR1A | 43151 | ES | 9.47E-12 | 7.12E-10 | 0.042317647 | 0.00558 | 2.026015411 |
| C17orf62 | 44388 | ES | 0.002013773 | 0.015701786 | 0.055928105 | 0.00774 | 1.977665331 |
| MED16 | 46334 | ES | 0.00161504 | 0.013384176 | 0.008820645 | 0.00092 | 2.260476624 |
| INSR | 47099 | ES | 0.000862442 | 0.008457337 | 0.098493162 | 0.0208 | 1.555034142 |
| ZNF561 | 47368 | ES | 5.08E-20 | 8.78E-18 | 0.076106164 | 0.003575 | 3.058164089 |
| SUGP2 | 48549 | ES | 6.29E-36 | 3.26E-33 | 0.208183871 | 0.00662 | 3.448326314 |
| URI1 | 48866 | ES | 0.004258393 | 0.027217866 | 0.052961157 | 0.0074 | 1.968078758 |
| RTN2 | 50466 | ES | 1.00E-30 | 3.86E-28 | 0.347659211 | 0.07112 | 1.586854129 |
| NAPA | 50661 | ES | 0.000792135 | 0.007922927 | 0.000142857 | 2.00E-05 | 1.966112856 |
| NDUFA3 | 51782 | ES | 0.000425584 | 0.004865248 | 0.023843871 | 0.00514 | 1.534474122 |
| FKBP1B | 52804 | ES | 6.14E-09 | 3.06E-07 | 0.036421477 | 0.00248 | 2.686900054 |
| CGREF1 | 52940 | ES | 4.72E-08 | 2.02E-06 | 0.449387681 | 0.05048 | 2.186308729 |
| PREPL | 53440 | ES | 6.02E-16 | 7.18E-14 | 0.080555034 | 0.00422 | 2.949105469 |
| PREPL | 53442 | ES | 8.83E-12 | 6.71E-10 | 0.258018644 | 0.0201 | 2.552312031 |
| PREPL | 53447 | ES | 1.75E-07 | 6.69E-06 | 0.203887603 | 0.02076 | 2.28454082 |
| AHSA2 | 53691 | ES | 0.006415965 | 0.03601782 | 0.011194156 | 0.0021 | 1.673454498 |
| ERMN | 55639 | ES | 0.004254136 | 0.027202224 | 0.001977419 | 0.00024 | 2.108908994 |
| GAD1 | 55908 | ES | 1.71E-12 | 1.39E-10 | 0.078080189 | 0.01562 | 1.609184214 |
| GORASP2 | 55918 | ES | 0.000776787 | 0.007805779 | 0.008235065 | 0.00178 | 1.531787885 |
| MAP2 | 57224 | ES | 1.13E-44 | 1.21E-41 | 0.277011039 | 0.01476 | 2.932136538 |
| MAP2 | 57225 | ES | 2.25E-36 | 1.25E-33 | 0.326545455 | 0.03126 | 2.346229832 |
| BCS1L | 57530 | ES | 4.17E-07 | 1.47E-05 | 0.614493056 | 0.06064 | 2.315842887 |
| BCS1L | 57534 | ES | 0.000643199 | 0.006742829 | 0.071786441 | 0.01016 | 1.955237168 |
| BCS1L | 57538 | ES | 2.87E-20 | 5.01E-18 | 0.304206667 | 0.02034 | 2.705117904 |
| BCS1L | 57545 | ES | 2.58E-05 | 0.000508432 | 0.425555556 | 0.05456 | 2.054094491 |
| BCS1L | 57553 | ES | 2.61E-07 | 9.56E-06 | 0.135927857 | 0.01672 | 2.095518675 |
| PID1 | 57843 | ES | 0.000122825 | 0.001814216 | 0.017058278 | 0.00366 | 1.53917246 |
| HDLBP | 58349 | ES | 0.001616428 | 0.013388302 | 0.003578065 | 0.00056 | 1.854640512 |
| ATG4B | 58403 | ES | 1.24E-05 | 0.000277417 | 0.006549032 | 0.00092 | 1.962698901 |
| MANBAL | 59340 | ES | 3.15E-37 | 1.98E-34 | 0.378361438 | 0.06974 | 1.691075882 |
| MANBAL | 59341 | ES | 9.13E-11 | 6.04E-09 | 0.002593377 | 0.00012 | 3.07322461 |
| MANBAL | 59343 | ES | 2.57E-43 | 2.41E-40 | 0.439797368 | 0.06252 | 1.950827589 |
| ZHX3 | 59412 | ES | 6.16E-05 | 0.001030529 | 0.016962727 | 0.001775 | 2.257218001 |
| APP | 60286 | ES | 3.59E-05 | 0.000669753 | 0.769771613 | 0.1524 | 1.619585221 |
| APP | 60287 | ES | 1.13E-06 | 3.52E-05 | 0.035587742 | 0.00436 | 2.099529194 |
| HPS4 | 61507 | ES | 0.0009515 | 0.009070319 | 0.084106081 | 0.01594 | 1.663247199 |
| ATP2B2 | 63372 | ES | 4.19E-14 | 4.11E-12 | 0.827646087 | 0.05456 | 2.719284618 |
| IP6K2 | 64757 | ES | 6.88E-06 | 0.000165972 | 0.102314286 | 0.01774 | 1.752227332 |
| IP6K2 | 64760 | ES | 1.11E-06 | 3.46E-05 | 0.297330968 | 0.05426 | 1.701058581 |
| IP6K2 | 64762 | ES | 2.12E-08 | 9.77E-07 | 0.037936364 | 0.00426 | 2.186640955 |
| DAG1 | 64886 | ES | 8.54E-06 | 0.000200337 | 0.01352 | 0.00136 | 2.296685371 |
| ABHD6 | 65429 | ES | 0.000545335 | 0.005914875 | 0.005127211 | 0.00068 | 2.020224305 |
| ROBO2 | 65642 | ES | 1.52E-27 | 4.67E-25 | 0.245435664 | 0.01366 | 2.888562999 |
| ACPL2 | 67065 | ES | 5.58E-08 | 2.37E-06 | 0.053394444 | 0.0052 | 2.329048078 |
| EIF4A2 | 68054 | ES | 2.82E-06 | 7.70E-05 | 0.137898065 | 0.02794 | 1.596455395 |
| EIF4E | 70013 | ES | 1.57E-05 | 0.000337947 | 0.017943226 | 0.0027 | 1.893960878 |
| HADH | 70285 | ES | 5.63E-15 | 6.05E-13 | 0.013325161 | 0.00252 | 1.665395173 |
| NDUFC1 | 70621 | ES | 2.08E-08 | 9.65E-07 | 0.095341722 | 0.02 | 1.561735236 |
| SORBS2 | 71384 | ES | 4.01E-37 | 2.41E-34 | 0.360884091 | 0.0396 | 2.209727711 |
| BRD9 | 71464 | ES | 1.08E-06 | 3.41E-05 | 0.018537419 | 0.00192 | 2.267466171 |
| GPBP1 | 72130 | ES | 0.002569191 | 0.018812787 | 0.067303636 | 0.0104 | 1.867408461 |
| TRAPPC13 | 72245 | ES | 1.91E-06 | 5.49E-05 | 0.322305229 | 0.06472 | 1.605428739 |
| GNPDA1 | 73865 | ES | 5.62E-23 | 1.22E-20 | 0.0933375 | 0.00242 | 3.652454416 |
| DST | 76569 | ES | 5.54E-10 | 3.29E-08 | 0.727966207 | 0.1499 | 1.580286223 |
| EPB41L2 | 77555 | ES | 8.23E-18 | 1.13E-15 | 0.387069014 | 0.04132 | 2.237256364 |
| EPB41L2 | 77558 | ES | 3.93E-14 | 3.91E-12 | 0.200780986 | 0.02446 | 2.105175561 |
| EPB41L2 | 77562 | ES | 4.03E-19 | 6.24E-17 | 0.465421138 | 0.0348 | 2.593325283 |
| EPB41L2 | 77563 | ES | 0.000403265 | 0.004703095 | 0.614243624 | 0.12948 | 1.556865202 |
| EPB41L2 | 77565 | ES | 1.28E-21 | 2.57E-19 | 0.307271545 | 0.0188 | 2.793874997 |
| EPB41L2 | 77566 | ES | 3.26E-05 | 0.000620586 | 0.399686111 | 0.0771 | 1.645576236 |
| EPB41L2 | 77574 | ES | 1.58E-07 | 6.12E-06 | 0.543929524 | 0.0615 | 2.179782512 |
| EPB41L2 | 77577 | ES | 4.02E-10 | 2.43E-08 | 0.391644737 | 0.038075 | 2.330797247 |
| EPB41L2 | 77595 | ES | 3.69E-09 | 1.90E-07 | 0.107860265 | 0.0157 | 1.927175834 |
| WIPI2 | 78656 | ES | 2.71E-09 | 1.44E-07 | 0.264250968 | 0.03268 | 2.090136017 |
| ZDHHC4 | 78750 | ES | 5.44E-07 | 1.86E-05 | 0.154185714 | 0.02528 | 1.808144243 |
| GGCT | 79125 | ES | 5.68E-07 | 1.93E-05 | 0.010958065 | 0.002 | 1.70092849 |
| CAMK2B | 79489 | ES | 1.66E-17 | 2.23E-15 | 0.1491 | 0.00454 | 3.49169021 |
| UPP1 | 79640 | ES | 0.002385186 | 0.017847933 | 0.037200649 | 0.00808 | 1.526934344 |
| SUMF2 | 79799 | ES | 0.00829522 | 0.042911921 | 0.155535556 | 0.0255 | 1.808195906 |
| STX1A | 80018 | ES | 3.05E-25 | 8.19E-23 | 0.390475781 | 0.05372 | 1.983580576 |
| DTX2 | 80177 | ES | 2.34E-12 | 1.89E-10 | 0.131325191 | 0.01 | 2.575091527 |
| CCDC132 | 80466 | ES | 0.000551663 | 0.005974887 | 0.035058955 | 0.0073 | 1.569156731 |
| CCDC136 | 81718 | ES | 1.73E-14 | 1.79E-12 | 0.239916923 | 0.02626 | 2.212245841 |
| FAM86B1 | 82694 | ES | 0.001062823 | 0.009887017 | 0.801702679 | 0.13955 | 1.748314854 |
| AP3M2 | 83569 | ES | 4.54E-18 | 6.57E-16 | 0.11605461 | 0.01352 | 2.149890785 |
| C8orf59 | 84336 | ES | 1.38E-11 | 1.01E-09 | 0.155587097 | 0.0287 | 1.69030856 |
| C8orf59 | 84337 | ES | 6.31E-11 | 4.43E-09 | 0.029705161 | 0.00384 | 2.045848445 |
| ENPP2 | 85007 | ES | 4.72E-08 | 2.02E-06 | 0.034885496 | 0.00596 | 1.76700068 |
| NDRG1 | 85238 | ES | 2.83E-07 | 1.03E-05 | 0.334380172 | 0.05848 | 1.743593771 |
| ZNF707 | 85488 | ES | 0.008475335 | 0.043565851 | 0.144882727 | 0.02796 | 1.64514972 |
| PTPRD | 85850 | ES | 5.56E-06 | 0.000137421 | 0.843999281 | 0.12284 | 1.927268947 |
| ABCA2 | 88253 | ES | 0.006086166 | 0.034805329 | 0.01022844 | 0.00054 | 2.941358251 |
| KDM5C | 89207 | ES | 1.02E-12 | 8.75E-11 | 0.115549306 | 0.0189 | 1.810535405 |
| ARMCX5 | 89699 | ES | 5.75E-08 | 2.43E-06 | 0.36554966 | 0.0278 | 2.576366118 |
| MORF4L2 | 89771 | ES | 3.26E-06 | 8.70E-05 | 0.790727211 | 0.11802 | 1.902098941 |
| IDS | 90294 | ES | 3.36E-07 | 1.21E-05 | 0.05403871 | 0.00992 | 1.695147714 |
| PDZD4 | 90509 | ES | 0.000565132 | 0.006072644 | 0.880477778 | 0.18718 | 1.548393969 |
| GRIA2 | 96827 | ES | 0.000317848 | 0.003865864 | 0.029983088 | 0.00266 | 2.422307374 |
| MRPL55 | 117443 | ES | 2.10E-05 | 0.000429343 | 0.301932479 | 0.0668 | 1.508500331 |
| EPB41L2 | 150730 | ES | 7.90E-05 | 0.001264095 | 0.610511628 | 0.1052 | 1.75843404 |
| NLRP1 | 204008 | ES | 0.000181447 | 0.00247073 | 0.103985926 | 0.0231 | 1.504422945 |
| HNRNPA1 | 212643 | ES | 1.08E-19 | 1.76E-17 | 0.096224286 | 0.01306 | 1.997127652 |
| CNOT10 | 63822 | ME | 0.000116905 | 0.001117092 | 0.04886129 | 0.32516 | -1.895331895 |
| GPATCH2L | 120913 | ME | 0.001470194 | 0.00743745 | 0.032985246 | 0.300225 | -2.208471826 |
| NSMF | 193275 | ME | 0.000802957 | 0.004826979 | 0.064945161 | 0.73316 | -2.423820718 |
| PKM | 203434 | ME | 0.000898043 | 0.004826979 | 0.095635484 | 0.7624 | -2.075927431 |
| C2CD5 | 251535 | ME | 0.00045426 | 0.00312531 | 0.033117532 | 0.69852 | -3.048900987 |
| DLGAP4 | 393774 | ME | 0.000894591 | 0.004826979 | 0.01566129 | 0.54476 | -3.549153247 |
| DLG3 | 89383 | ME | 7.05E-10 | 1.73E-08 | 0.87131745 | 0.06742 | 2.559064666 |
| ZNF585B | 101324 | ME | 1.33E-17 | 5.71E-16 | 0.320368548 | 0.05278 | 1.803339716 |
| OPN3 | 204971 | ME | 0.013090311 | 0.044147715 | 0.008906452 | 0.0017 | 1.656147663 |
| LIPT1 | 211705 | ME | 0.000201066 | 0.001729165 | 0.06897027 | 0.01156 | 1.786124683 |
| EPHB2 | 1026 | RI | 0.000979708 | 0.009043755 | 0.005461333 | 0.23768 | -3.773232271 |
| RPLP2 | 13772 | RI | 0.00877218 | 0.041564708 | 0.000869032 | 0.00778 | -2.191931372 |
| RBM7 | 18823 | RI | 0.000119449 | 0.001857475 | 0.036096129 | 0.24458 | -1.913356823 |
| ANKLE2 | 25300 | RI | 8.00E-05 | 0.00142578 | 0.070288889 | 0.46126 | -1.881348142 |
| LRRC57 | 30183 | RI | 0.000411285 | 0.004671305 | 0.059488194 | 0.35526 | -1.787072036 |
| RPS15A | 34253 | RI | 0.000667904 | 0.006939615 | 0.001854194 | 0.01152 | -1.826634799 |
| CHCHD5 | 55023 | RI | 0.000106919 | 0.001769509 | 0.017935484 | 0.14736 | -2.106097483 |
| TM4SF1 | 67223 | RI | 0.004038809 | 0.023278406 | 0.00868 | 0.04474 | -1.639846427 |
| TTC14 | 67733 | RI | 0.000256922 | 0.003344312 | 0.071837908 | 0.3644 | -1.623839855 |
| MAPK13 | 75952 | RI | 2.58E-05 | 0.000557052 | 0.0315125 | 0.364125 | -2.447112923 |
| DYNLT1 | 78278 | RI | 0.001427743 | 0.011566716 | 0.022683871 | 0.10228 | -1.506060009 |
| YPEL4 | 15880 | RI | 8.83E-06 | 0.000249474 | 0.171877778 | 0.03384 | 1.625140095 |
| PRRT2 | 35953 | RI | 1.07E-27 | 4.95E-25 | 0.848507792 | 0.05892 | 2.667298677 |
| PRRT2 | 35954 | RI | 2.68E-39 | 6.22E-36 | 0.422034194 | 0.03064 | 2.622779993 |
| STX1B | 36214 | RI | 1.12E-30 | 8.68E-28 | 0.295928148 | 0.01014 | 3.373628684 |
| VAMP2 | 39105 | RI | 5.78E-15 | 1.34E-12 | 0.113180645 | 0.01314 | 2.153324159 |
| FDXR | 43317 | RI | 4.26E-07 | 2.10E-05 | 0.051020395 | 0.0041 | 2.521238476 |
| CCDC74A | 55388 | RI | 0.008638644 | 0.041100078 | 0.270681208 | 0.054933333 | 1.594821446 |
| DBN1 | 74773 | RI | 0.005735894 | 0.030332722 | 0.001093548 | 2.00E-04 | 1.698865722 |
| ABCB8 | 82296 | RI | 0.008973469 | 0.042062677 | 0.007824675 | 0.00154 | 1.625499827 |
| AP3M2 | 83565 | RI | 2.66E-07 | 1.43E-05 | 0.003113768 | 0.00012 | 3.256097142 |
| DCTN3 | 86186 | RI | 4.41E-06 | 0.000143987 | 0.031666667 | 0.0054 | 1.768865649 |
